# Elucidating the Complex Oxidation Behavior of Aqueous H_3_PO_3_ on Pt Electrodes via *In Situ* Tender X-ray Absorption Near-Edge Structure Spectroscopy at the P *K*-Edge

**DOI:** 10.1021/jacs.3c12381

**Published:** 2024-03-09

**Authors:** Romualdus Enggar Wibowo, Raul Garcia-Diez, Tomas Bystron, Marianne van der Merwe, Martin Prokop, Mauricio D. Arce, Anna Efimenko, Alexander Steigert, Milan Bernauer, Regan G. Wilks, Karel Bouzek, Marcus Bär

**Affiliations:** † Department of Interface Design, Helmholtz-Zentrum Berlin für Materialien und Energie GmbH (HZB), Albert-Einstein-Straße 15, 12489 Berlin, Germany; ‡ Department of Inorganic Technology, University of Chemistry and Technology Prague, Technicka 5, Prague 6 166 28, Czech Republic; § Departamento Caracterización de Materiales, INN-CNEA-CONICET, Centro Atómico Bariloche, Avenida Bustillo 9500, S. C. de Bariloche, Rio Negro 8400, Argentina; ∥ Energy Materials In-situ Laboratory Berlin (EMIL), Helmholtz-Zentrum Berlin für Materialien und Energie GmbH (HZB), Albert-Einstein Straße 15, 12489 Berlin, Germany; ⊥ Institute of Nanospectroscopy, Helmholtz-Zentrum Berlin für Materialien und Energie GmbH (HZB), Albert-Einstein-Straße 15, 12489 Berlin, Germany; # Department of Chemistry and Pharmacy, Friedrich-Alexander-Universität Erlangen-Nürnberg, Egerlandstraße 3, 91058 Erlangen, Germany; ∇ Department of X-ray Spectroscopy at Interfaces of Thin Films, Helmholtz Institute Erlangen-Nürnberg for Renewable Energy (HI ERN), Albert-Einstein-Straße 15, 12489 Berlin, Germany

## Abstract

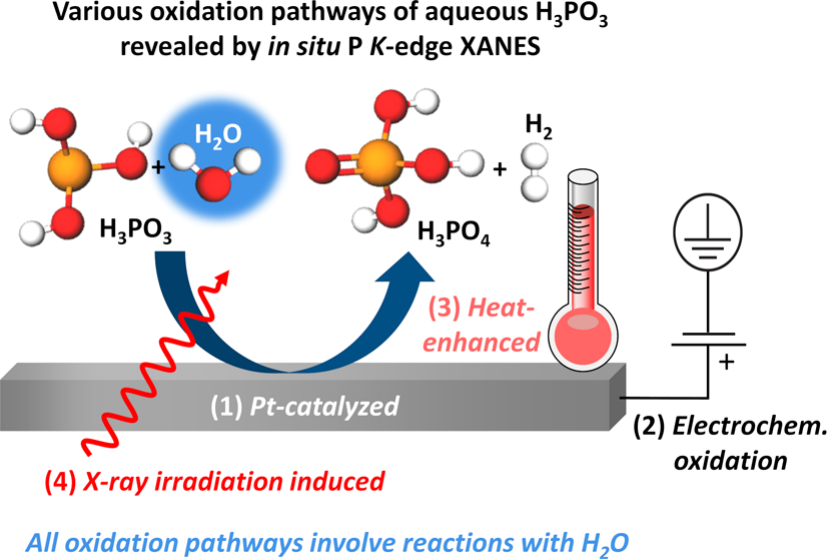

*In situ* tender X-ray absorption near-edge structure (XANES) spectroscopy at the P *K*-edge was utilized to investigate the oxidation mechanism of aqueous H_3_PO_3_ on Pt electrodes under various conditions relevant to high-temperature polymer electrolyte membrane fuel cell (HT-PEMFC) applications. XANES and electrochemical analysis were conducted under different tender X-ray irradiation doses, revealing that intense radiation induces the oxidation of aqueous H_3_PO_3_ via H_2_O yielding H_3_PO_4_ and H_2_. A broadly applicable experimental procedure was successfully developed to suppress these undesirable radiation-induced effects, enabling a more accurate determination of the aqueous H_3_PO_3_ oxidation mechanism. *In situ* XANES studies of aqueous 5 mol dm^–3^ H_3_PO_3_ on electrodes with varying Pt availability and surface roughness reveal that Pt catalyzes the oxidation of aqueous H_3_PO_3_ to H_3_PO_4_. This oxidation is enhanced upon applying a positive potential to the Pt electrode or raising the electrolyte temperature, the latter being corroborated by complementary ion-exchange chromatography measurements. Notably, all of these oxidation processes involve reactions with H_2_O, as further supported by XANES measurements of aqueous H_3_PO_3_ of different concentrations, showing a more pronounced oxidation in electrolytes with a higher H_2_O content. The significant role of water in the oxidation of H_3_PO_3_ to H_3_PO_4_ supports the reaction mechanisms proposed for various chemical processes observed in this work and provides valuable insights into potential strategies to mitigate Pt catalyst poisoning by H_3_PO_3_ during HT-PEMFC operation.

## 1 Introduction

High-temperature polymer electrolyte membrane fuel cells (HT-PEMFCs) represent attractive choices for a green stationary power source. By utilizing H_3_PO_4_-doped polybenzimidazole-based membranes as proton conductors, the HT-PEMFCs are operated at elevated temperatures around 120–180 °C and thus have several advantages compared to the lower-temperature counterparts (LT-PEMFCs) operating at the temperature around 65–85 °C.^1−3^ These advantages include operation with a lower H_2_ purity feedstock due to a higher resistance against CO poisoning,^4,5^ the simpler management of generated water,^1,2^ and the possibility of operation coupled with reformers.^6−8^ However, the use of H_3_PO_4_-doped membranes also has a drawback, such as H_3_PO_4_ leaching out of the membrane, which then results in Pt catalyst degradation^9,10^ and adsorption of H_3_PO_4_ and its anion, e.g., H_2_PO_4_^–^ at the Pt catalyst leading to the poisoning of Pt.^11−14^ Moreover, the high operating temperatures also lead to increased degradation rates and long startup times, as well as a larger ohmic loss due to membrane dehydrations.^1,2,15^

On top of these challenges, recent studies also suggest a possible reduction of H_3_PO_4_ to H_3_PO_3_ during operation conditions of HT-PEMFCs,^16−19^ which might negatively impact the performance of HT-PEMFCs.^16−19^ Specifically, the detrimental effect of H_3_PO_3_ on the oxygen reduction reaction (ORR) kinetics at the Pt electrode^20^ and the stronger adsorption strength of H_3_PO_3_ compared to H_3_PO_4_ on the Pt catalysts were shown.^21^ The strong adsorption of H_3_PO_3_ on Pt may cause Pt catalyst poisoning. Considering the possible transport of H_3_PO_3_ to the cathode during HT-PEMFC operation, Pt catalyst poisoning might hinder the ORR, resulting in a significant performance loss. However, our previous study revealed that at room temperature without external polarization (i.e., at open-circuit potential, OCP), Pt also catalyzes the chemical oxidation of aqueous H_3_PO_3_ to H_3_PO_4_ via reactions with H_2_O.^22^ This process illustrates the complexity of the interactions at the Pt|H_3_PO_*x*_ interface: while H_3_PO_3_ can cause Pt catalyst poisoning, Pt might also oxidize H_3_PO_3_ back to H_3_PO_4_, especially given the formation of H_2_O on the cathode of HT-PEMFCs during operation. Therefore, further investigation is necessary to elucidate the oxidation behavior of aqueous H_3_PO_3_ under conditions relevant to HT-PEMFC operations, e.g., at electrode potentials similar to those present at the cathode of HT-PEMFCs and/or at elevated temperatures, before proceeding to more complex (e.g., true *operando*) studies.

X-ray absorption near-edge structure (XANES) spectroscopy is a powerful tool allowing to shed light on the oxidation behavior of H_3_PO_3_. XANES is element-specific, sensitive to the oxidation state, and provides information on the unoccupied electronic states of materials.^23,24^ Furthermore, recent advances in the design and use of three-electrode flow cells that are compatible with the use of soft and tender X-rays now allow soft/tender XANES measurements of the solid-electrode|liquid-electrolyte interface during chemical reactions (i.e., enabling *in situ* or *operando* studies).^25−28^ Additionally, in our previous study, we demonstrated that tender XANES at the P *K-*edge can be used to differentiate between H_3_PO_3_ and H_3_PO_4_, as well as their mixture in aqueous solutions.^29^ Thus, it is an excellent technique for elucidating the H_3_PO_3_ oxidation behavior under conditions close to HT-PEMFC operation. However, the use of highly brilliant synchrotron radiation for XANES measurements may also induce undesired changes to the investigated system.^30,31^ Effects such as radiolysis or radiation damage (also referred to as beam damage)^32,33^ may occur due to the interactions of secondary electrons with the sample, causing ionic fragmentation during XANES measurements.^34,35^ Furthermore, numerous studies have been made on the radiolysis of water, revealing the formation of several radicals such as HO^•^, H^•^, and HO_2_^•^ upon the interaction of energetic particles, including photons, with water.^36−40^ Some works have also highlighted the effect of radiolysis on concentrated H_3_PO_4_ solutions, revealing the formation of phosphoric acid radicals during pulse radiolysis.^41,42^ These effects may influence the recorded XANES data, thereby corrupting the interpretation of the induced spectral changes. Despite these challenges, synchrotron-based light sources currently remain as the most suitable choice for *in situ* XANES investigations of the complex solid-electrode|liquid-electrolyte system. This is because probing such a system requires a high photon flux that is sufficient to acquire XANES spectra with an adequate signal-to-noise ratio within the time scales relevant to the experiment. Despite recent advances in laboratory-based XANES setups, the photon fluxes of laboratory-based X-ray sources are still orders of magnitude lower than those of synchrotron-based light sources,^43^ and their applicability for complex *in situ* studies of the solid-electrode|liquid-electrolyte system is yet to be proven. Hence, synchrotron-based *in situ* XANES studies of catalysts/acidic aqueous electrolytes have to be performed carefully to make sure that the observed spectral change indeed corresponds to the interaction of interest and not due to additional radiation-induced effects.

In this work, *in situ* P *K-*edge XANES was performed to investigate the oxidation behavior of aqueous H_3_PO_3_ in the presence of a Pt electrode, under different conditions relevant to the HT-PEMFC applications. Initial XANES measurements of aqueous H_3_PO_4_, H_3_PO_3_, and H_3_PO_2_ solutions were made under different irradiation doses. These measurements serve not only as a reference but are also used for the determination of radiation-induced effects on the investigated aqueous H_3_PO_*x*_ systems. In particular, experiments in aqueous H_3_PO_2_ provide further insights into the oxidation mechanism of H_3_PO_3_, given that both P-containing acids in the aqueous mixture are thermodynamically unstable.^44^ Based on the irradiation dose-dependent XANES measurements, a generally applicable experimental procedure was developed to suppress undesired radiation-induced effects, thus enabling an accurate determination of the H_3_PO_3_ oxidation mechanism. Subsequently, *in situ* P *K-*edge XANES measurements of the aqueous H_3_PO_3_|Pt electrode system were performed at several experimental conditions: varying Pt electrode availability and roughness, temperature, electrode potential, and electrolyte concentration of different H_2_O content. For further insights into the oxidation mechanism of H_3_PO_3_, complementary ion-exchange chromatography (IEC) measurements were carried out on an aqueous H_3_PO_3_ electrolyte that has been aged at elevated temperatures with and without the presence of Pt.

## 2 Experimental Section

### 2.1 Preparation of the Electrodes and Electrolytes

#### 2.1.1 Electrode Preparation and Characterization

To fabricate a planar Pt electrode, a 5 nm thick Ti adhesion layer was first sputtered onto a 12 μm Kapton membrane (Sigma-Aldrich). Subsequently, a 15 nm thick Pt layer was sputtered on top of the Ti layer. The sputtering process was carried out in DC magnetron mode (PREVAC, project 500) at a process pressure of 4 × 10^–3^ mbar (base pressure is 1 × 10^–8^ mbar), using a sputter deposition rate of approximately 5 nm min^–1^ at 50 W. Argon gas (99.999%, Air Liquide) was used as the working gas during the sputtering process.

In addition to the planar Pt electrode, a rougher Pt black electrode with a higher surface area was also prepared (hereafter referred to as “Pt black”). The Pt black electrode was fabricated by electrochemical deposition using the previously described planar Pt electrode as the substrate. The electrodeposition was conducted *in situ* using the electrochemical flow cell, immediately after *in situ* P *K*-edge XANES experiments with planar Pt. This approach ensured a similar sample environment between *in situ* XANES measurements of planar Pt and Pt black. To ensure that electrodeposition was carried out on a clean planar Pt substrate, 20 mL of Milli-Q water was flushed into the reactor chamber. Subsequently, around 20 mL of 0.5 mol dm^–3^ H_2_SO_4_ (prepared by diluting 95 wt % H_2_SO_4_ [Merck] with Milli-Q water) was flushed into the reactor. Using this 0.5 mol dm^–3^ H_2_SO_4_ solution, planar Pt was activated by electrochemical cycling with the scan rate of 50 mV s^–1^ within the potential range of +0.05 V_RHE_ to +1.0 V_RHE_, i.e., in the water stability window. After activation, 2 mol dm^–3^ HCl (prepared by diluting 37 wt % HCl [Carl Roth] with Milli-Q water) and 2 wt % H_2_PtCl_6_ (99.9%, Alfa Aesar) were injected into the reactor. Subsequently, the electrodeposition was conducted by drawing current densities of −8.8 mA cm^–2^ to the planar Pt working electrode for 53 s. This electrodeposition time ensured that deposited Pt black possesses a significantly higher roughness factor compared to the planar Pt electrode while being only ∼10 nm thicker than planar Pt. As a result, such prepared Pt electrodes still possess a high enough X-ray transmittance, regardless of the surface modification (87 and 92%, for Pt black and planar Pt, respectively). These transmission values were determined for the incoming X-ray with a photon energy of 2156.5 eV and an incidence angle perpendicular to the sample surface, using the Lawrence Berkeley Laboratory X-ray transmission database, which is based on the model published in ref (45). Details for the Pt black electrodeposition profile and thickness estimation are given in Section S1, Figure S1. The electrodeposition was carried out using a Pt wire (99.9%, Alfa Aesar) counter electrode and a reversible hydrogen reference electrode (Mini HydroFlex, Gaskatel). Following electrodeposition, a cleaning procedure akin to that employed for the planar Pt electrode was applied to the Pt black electrode, ensuring a clean Pt black surface for the subsequent *in situ* XANES experiments. This procedure includes flushing of the reactor chamber with 20 mL of Milli-Q water, followed by an injection of 20 mL of 0.5 mol dm^–3^ H_2_SO_4_ and electrochemical cycling with this electrolyte. Subsequently, the chamber was flushed with 20 mL of Milli-Q water, and then the aqueous electrolyte of interest was injected for the *in situ* XANES experiments.

To quantify the increase in the surface area after Pt black deposition, the electrochemically active surface area (ECSA) of both Pt electrodes was estimated through hydrogen underpotential deposition (H_UPD_). The comparison of the ECSA indicated that the Pt black electrode possesses an approximately 5 times larger ECSA than the planar Pt electrode. Scanning electron microscopy (SEM; ZEISS, MERLIN) and atomic force microscopy (AFM; Park System, XE-70) were also performed on both Pt electrodes to confirm the increased roughness of Pt black. SEM and AFM images of Pt black were obtained after the XANES experiments (i.e., at the end of the beamtime campaign) and compared to the SEM and AFM images of planar Pt taken before the start of the XANES experiment. The AFM-derived roughness factor confirmed that Pt black is roughly 5 times rougher than planar Pt, consistent with the ECSA comparison. Detailed experimental procedures and results for the ECSA determination via H_UPD_ and SEM images of these electrodes can be found in Section S2 and Figure S2.

In this experiment, rough Pt black serves as an electrode with a considerably higher roughness factor compared to the planar Pt electrode, albeit without carbon support, unlike commercial Pt/C catalysts. The use of unsupported Pt black was preferred over the commercial Pt/C catalysts typically used for fuel cell application to circumvent potential complexities associated with the use of carbon supports, such as carbon corrosion at high temperatures. Additionally, both the planar Pt and Pt black electrodes exhibit an analogous cyclic voltammogram response with catalyst ink prepared with the commercial Pt/C catalysts, as presented in Section S2.

#### 2.1.2 Electrolyte Preparation

The aqueous electrolytes used within this study were prepared by diluting either H_3_PO_4_ (99.99 wt %, Merck), H_3_PO_3_ (99 wt %, Merck), or H_3_PO_2_ (50 wt % in H_2_O, Merck) with Milli-Q water (conductivity ∼0.055 μS cm^–1^, Q-POD) until the concentration of interest was achieved. In this study, aqueous electrolytes of H_3_PO_4_, H_3_PO_3_, and H_3_PO_2_ were prepared with the concentrations of 5 mol dm^–3^. Additionally, aqueous H_3_PO_3_ electrolytes with concentrations of 0.1 and 1 mol dm^–3^ were also prepared. Prior to the *in situ* P *K-*edge XANES measurements, all electrolytes were thoroughly mixed and deaerated by purging with N_2_ (99.9999%, Linde) for approximately 30 min.

### 2.2 *In Situ* P *K*-Edge XANES Measurements and Related Electrochemical Characterizations

The spectroscopic and electrochemical characterizations were conducted using a three-electrode flow cell designed for and used at the OÆSE end-station, which is located at the two-color EMIL beamline at the BESSY II, operated by HZB. For the P *K-*edge XANES measurements, the hard X-ray branch of the EMIL beamlines, based on a CPMU17 undulator, was utilized in combination with a Si(111) double crystal monochromator (DCM) to monochromatize the incoming X-rays. The DCM was operated in channel-cut mode, i.e., the distance between crystals was fixed during the XANES measurements. The beam was focused on the sample position by two consecutive optical mirrors to a spot size of approximately 237 μm × 37 μm. The sample was probed with incoming X-ray at an angle of incidence nearly perpendicular to the sample surface. The fluorescence yield (FY) XANES signal was recorded in reflection geometry at an angle of 45° to the sample surface using a photodiode (ODD-AXU-010, Optodiode).

In the three-electrode flow cell, an X-ray transparent membrane (in this case, a 12 μm thick Kapton foil) was employed to separate the atmospheric pressure in the cell from the vacuum in the end-station and beamline (with a base pressure of <9 × 10^–8^ mbar in the OÆSE end-station). The excitation energy of the beamline was calibrated by aligning the absorption spectrum of a 5 mol dm^–3^ H_3_PO_4_ electrolyte behind a bare 12 μm Kapton membrane to the values reported in our previous investigation.^29^ Further details about the three-electrode flow cell and the OÆSE end-station can be found in Section S3, Figure S4.

#### 2.2.1 Electrochemical Characterization and Electrode Preconditioning

The working electrodes used for the electrochemical characterizations were either the prepared planar Pt electrode or the rough Pt black electrode. In both cases, the counter electrode and the reference electrode were a Pt wire (99.9%, Alfa Aesar) and a reversible hydrogen electrode (Mini HydroFlex, Gaskatel), respectively. The electrochemical experiments were conducted using a BioLogic SP300 double-channel potentiostat.

Before conducting the *in situ* P *K-*edge XANES measurements, the working electrode was activated by potential cycling in the water stability window, ranging from +0.05 V_RHE_ to +1.0 V_RHE_, with a scan rate of 50 mV s^–1^. The potential cycling continued until no observable changes were detected in the region corresponding to the hydrogen desorption, i.e., the region around +0.05 V_RHE_ to +0.4 V_RHE_ at the positive-going potential scans.

Subsequently, P *K-*edge XANES measurements were performed using aqueous H_3_PO_3_ electrolytes with the working electrode being at (i) the OCP where no current was drawn to the working electrode (between approximately +0.35 V_RHE_ to +0.1 V_RHE_) and (ii) at three distinct positive potentials where the electrochemical oxidation of H_3_PO_3_ is expected to occur: +0.8, V_RHE_, +0.9 V_RHE_, and +1.0 V_RHE_.

Before setting the electrode to those aforementioned potentials, the electrode was subjected to three cycles of cyclic voltammogram (CV) in the potential range between +0.05 V_RHE_ to +1.0 V_RHE_ and the scan rate of 50 mV s^–1^ to “restore” the electrode surface. This procedure ensured that the electrode surface was brought back to a comparable initial state for each XANES measurement, i.e., to reduce the “electrode history” effect.^46^ Before commencing any XANES measurements, the electrode was maintained at the desired potential for approximately 2 min (until a steady chronoamperometry (CA) current response was achieved), ensuring a steady-state surface coverage of the electrode before data acquisition.

#### 2.2.2 Determination and Mitigation of Radiation-Induced Effects on the Pt/Aqueous H_3_PO_*x*_ XANES Data

To identify any radiation-induced effects in the Pt|aqueous H_3_PO_*x*_ XANES spectra (H_3_PO_*x*_ being H_3_PO_4_, H_3_PO_3_, and H_3_PO_2_), sequential P *K-*edge XANES measurements with two different radiation doses were performed on aqueous 5 mol dm^–3^ H_3_PO_*x*_ samples with the planar Pt electrode and the “Pt free” Kapton substrate. Roughly, 2.3 × 10^5^ and 5.4 × 10^5^ kGy of radiation were absorbed by the electrolyte, for “low irradiation dose” and “high irradiation dose” measurements, respectively (hereafter referred to as “low dose” and “high dose”). Detailed information on irradiation dose estimation can be found in Section S11. For the high dose measurements, the X-ray beam continuously irradiated the sample while recording the XANES spectra. Low dose measurements were achieved by rapidly closing and opening a beam-blocking valve (located just before the measurement chamber) during the XANES acquisition. Specifically, the valve is closed during the deadtime of the XANES acquisition, i.e., the time in which no acquisition is made, such as during the time in which the monochromator is set to a new energy. Using this method, the irradiation dose can be reduced by approximately 43%. This approach was used to minimize the irradiation dose during experiments since the spectra recorded with this approach display a higher signal-to-noise ratio compared to the spectra that were recorded with a radiation-attenuating filter, even though both receive a comparable irradiation dose. A comparison between XANES spectra recorded with the rapid X-ray beam-blocking approach and XANES spectra recorded with the radiation-attenuating filter is provided in Section S4 and Figure S5.

For each experimental condition, three sequential XANES spectra were recorded on different sample positions and were then averaged to increase the signal-to-noise ratio. To prevent the local increase in the irradiation dose, each measurement position was separated (from the previous) by at least the size of the beam spot both in the vertical and horizontal directions. Considering the geometry of the three-electrode flow cell (with an X-ray inlet/outlet diameter of 4.7 mm) and the beam spot size (237 μm × 37 μm), it was possible to probe the sample at several different measurement positions by making use of the motorized setup of the OÆSE end-station. To ensure that each measurement position has a comparable spectral background, fluorescence grid maps over the area of the three-electrode flow cell window were made with the photon energy of ≈2152.5 eV, which roughly corresponds to the white line position of H_3_PO_4_. With this approach, the fluorescence map is sensitive to the presence of H_3_PO_4_ (i.e., the oxidation product of H_3_PO_3_), enabling the selection of measurement positions with a similar spectral background intensity (as highlighted in Figure S6). To minimize irradiation influence during the acquisition of the grid map, an irradiation-attenuating filter was used to reduce the intensity of the incoming beam to roughly 21.2% of the original intensity. The grid map was scanned at an interval of 50 μm in both vertical and horizontal directions, with each data point in the map recorded for approximately 1 s. After averaging the three subsequently recorded XANES spectra, linear backgrounds were fitted and subtracted from the averaged spectrum before the spectrum was normalized. The respective standard deviations in spectral shapes of the XANES data are represented by the shaded area around the averaged spectra. This standard deviation serves as an indicator for the experimental reproducibility as well as the sensitivity of the spectral change upon change of experimental conditions, such as an increase in temperature or application of electrode potential.

To minimize radiation exposure to the sample, the XANES measurements were limited to the region near the white line (2145–2156.5 eV), where the most significant spectral change occurs between aqueous H_3_PO_3_ and aqueous H_3_PO_4_ (see ref (29)). This limited energy window reduced the acquisition time of one XANES scan to around 265 s, thereby minimizing the radiation dose and suppressing undesired irradiation-induced effects. These experiments were performed with the energy step of 0.25 eV and the energy resolution of approximately 0.64 eV. Considerations for energy resolutions are provided in Section S6. Additionally, a constant electrolyte flow of 0.05 mL min^–1^ was maintained during measurements (via the syringe pump Legato110, KD Scientific) to flush away possible side-products generated by irradiation effects in the ∼0.75 mL-sized flow cell reactor chamber. This ensures that the electrolyte in the chamber is renewed after three sequential XANES scans. The significance of this experimental procedure in minimizing undesirable radiation-induced effects, including performing sequential XANES scans at different measurement positions and the use of a constant electrolyte flow throughout the experiments, is highlighted by the sequential P *K*-edge XANES of planar Pt|5 mol dm^–3^ H_3_PO_3_ recorded without an electrolyte flow at either the same measurement position or different measurement positions, as detailed in Section S7 and Figure S7.

Additional insight into the radiation-induced effects on aqueous H_3_PO_3_ electrolytes on Pt electrodes was obtained through electrochemical characterizations, such as OCP monitoring during irradiation with different doses.

#### 2.2.3 *In Situ* P *K*-Edge XANES Measurements of Pt/Aqueous H_3_PO_3_ under Different Experimental Conditions: Potential Bias, Elevated Temperature, and Varying Aqueous H_3_PO_3_ Concentrations

*In situ P K-*edge XANES measurements were recorded for aqueous H_3_PO_3_ using Pt electrodes at different experimental conditions: varying Pt availability/roughness (“Pt free” Kapton substrate, flat planar Pt, and rough Pt black), temperature (at 25 and 75 °C), electrode potentials (at the OCP and positive potentials: +0.8 V_RHE_, +0.9 V_RHE_, and +1.0 V_RHE_), and electrolyte concentrations (0.1, 1, and 5 mol dm^–3^).

The temperature was controlled at the inlet of the three-electrode flow cell via a heating wire (Ni wire, Heraeus Hanau) sealed with a thermal insulating tape (K*-*Flex ST). To maintain a stable temperature inside the reaction chamber, additional heating elements (a silicone rubber fiberglass flexible heater, Omega Engineering) were mounted at the back of the three-electrode flow cell and were set to the desired temperature. Furthermore, to avoid a temperature gradient between the reaction chamber and the outlet line and ensure stable temperature control, the outlet temperature was controlled via an insulated heating wire, similar to the inlet line.

For precise monitoring of temperature, PFA-coated thermocouples (CASS-IM15G-300-PFA, Omega Engineering) were inserted into the reaction chamber of the three-electrode flow cell and on different key locations of the system to record temperatures before, inside, and after the flow cell reactor. PFA coating on thermocouples was used to prevent corrosion induced by the harsh experimental conditions, thereby preventing contamination of the electrolyte solutions. Detailed information on the heating scheme and temperature monitoring can be found in Section S8, Figure S8.

### 2.3 IEC Measurements of the Aqueous H_3_PO_3_ Electrolyte at Relevant Temperatures

Two aqueous H_3_PO_3_ electrolytes with a concentration of 10 mmol dm^–3^ H_3_PO_3_ are prepared by diluting crystalline H_3_PO_3_ (98 wt %, extra pure, Acros Organics) with deionized water from a DIWA purifier (conductivity <0.5 μS m^–1^, WATEK). For the first solution, 15 cm^3^ of the electrolyte was deaerated with N_2_ (99.995 vol %, SIAD) for 30 min and then kept in a sealed glass container and aged at 70 °C (i.e., a comparable temperature to the XANES measurements at an elevated temperature). For the second solution, 50 mg of the Pt/C catalyst HiSPEC 4000 (40 wt % Pt, Johnson Matthey) was added to 15 cm^3^ of the deaerated solution at 70 °C. Stirring was maintained throughout the experiment at 1000 rpm using a PTFE-sealed magnetic stirrer.

At specific time intervals during both experiments, 1 cm^3^ of the solution was withdrawn using a syringe equipped with a membrane filter (Chromafil O-20/15MS, Macherey-Nagel). The extracted sample and the experimental solution were then deaerated for 30 s. These processes were carried out at room temperature.

The 1 cm^3^ deaerated samples were subjected to analysis using a Dionex Integrion HPIC system. An ion-exchange precolumn Dionex IonPac AS19-4μm (2 × 50 mm^2^) and an analytical column Dionex IonPac AS19-4μm (2 × 250 mm^2^) were used in conjunction with an anion dynamic self-regulating suppressor ADRS 600, an auto sampler AS-AP, and a conductivity detector CR-ATC 600 for the analysis of inorganic anions (Thermo Scientific). The injected sample volume was 0.025 cm^3^. The mobile phase employed was 20 mmol dm^–3^ KOH solution, flowing at a rate of 0.25 cm^3^ min^–1^, and a suppressor current of 13 mA. The eluent was generated automatically by mixing KOH solution from a cartridge (EGC 500 KOH) with demineralized water. To quantify the concentration of H_3_PO_3_ and H_3_PO_4_ in the samples, calibration curves were derived using standard solutions prepared with deionized water, H_3_PO_3_, and H_3_PO_4_ (85 wt %, extra pure, Acros Organics).

For further insights into the effect of temperature, a similar IEC experiment was conducted using 10 mmol dm^–3^ H_3_PO_3_ with Pt/C dispersion that has been aged for the same duration, but at the temperature of 25 °C. This experiment was performed with a mobile phase of 15 mmol dm^–3^ KOH solution, flowing at a rate of 0.25 cm^3^ min^–1^, and a suppressor current of 10 mA. Note that for this experiment, the eluent concentration was adjusted to 15 mmol dm^–3^ KOH to enhance the peak separation between H_3_PO_3_ and H_3_PO_4_ species (i.e., to improve the separation of the observed retention times between H_3_PO_3_ and H_3_PO_4_).

## 3 Results and Discussion

### 3.1 Effect of Platinum on the Oxidation of Aqueous H_3_PO_3_ as Derived by P *K-*Edge XANES

**Figure 1:**
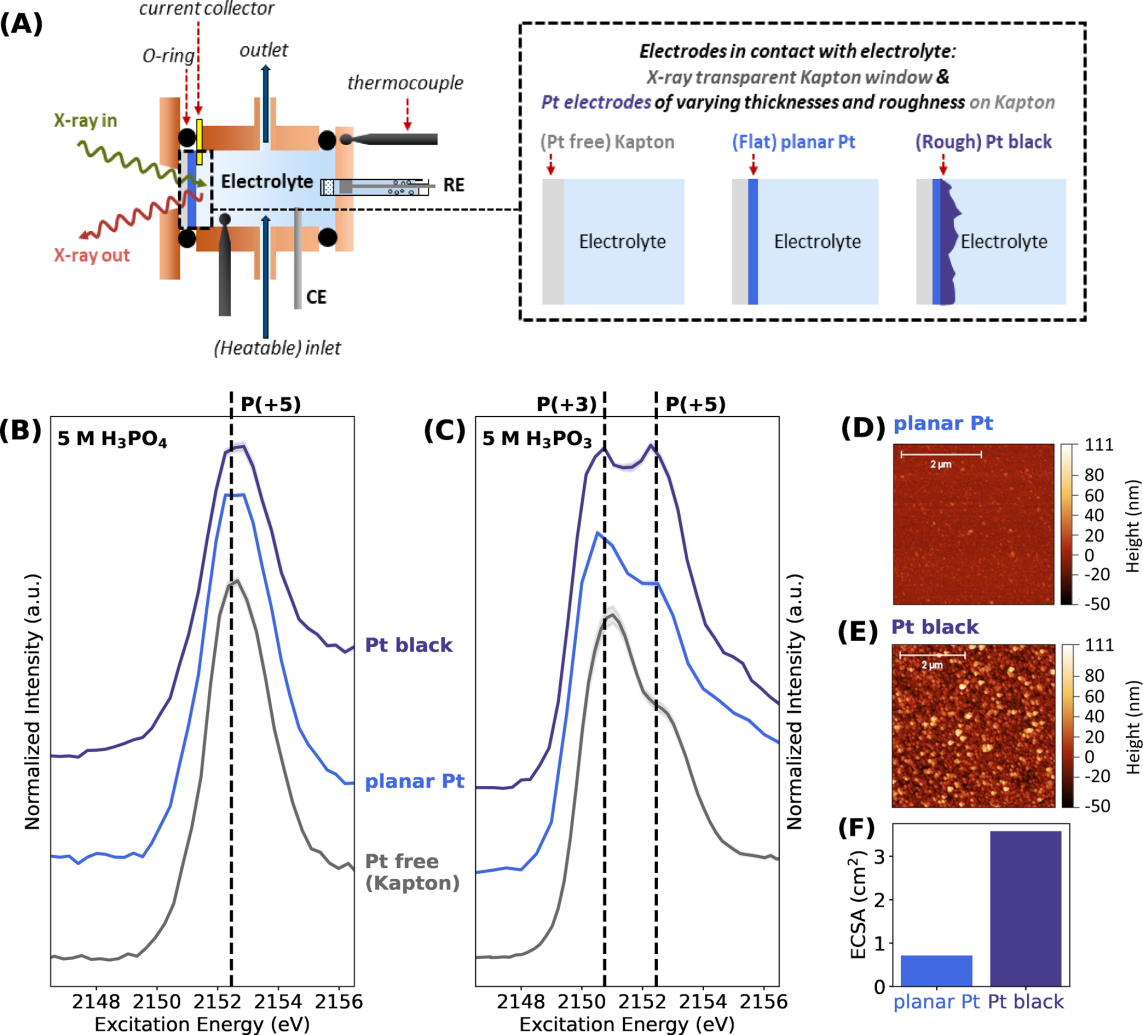
(A) Schematic presentation of the three-electrode flow cell as used at the OÆSE end-station and the different electrodes studied by *in situ* P *K*-edge XANES experiments. The length shown in the illustration is not up to scale. P *K-*edge XANES spectra of bare Kapton (“Pt free”), a flat planar Pt electrode, and a rougher Pt black electrode in contact with the 5 mol dm^–3^ (5 M) electrolyte of: (B) H_3_PO_4_ and (C) H_3_PO_3_. Measurements with Pt electrodes were performed at the OCP. The solid curves represent the average spectra of three measurements at different sample positions, with the shaded regions corresponding to the respective standard deviations of the individual measurements from the mean. The vertical dashed lines represent the characteristic white line positions of P-containing compounds with oxidation states of (+3) or (+5). XANES of 5 M H_3_PO_4_ with different electrode surfaces show negligible spectral change. In contrast, XANES of 5 M H_3_PO_3_ on the Pt black electrode reveals an increase in the spectral weight corresponding to P compounds with an oxidation state of +5 compared to the spectra recorded on the planar Pt electrode and bare Kapton. AFM images of (D) the planar Pt electrode and (E) the Pt black electrode illustrating the rougher surface topography of the Pt black electrode compared to the planar Pt electrode. (F) Electrochemically active surface area (ECSA) of the planar Pt electrode in comparison to the Pt black electrode. The Pt black electrode possesses ∼5 times the ECSA of the planar Pt electrode, in agreement with the roughness factor derived from AFM. For details on ECSA determination and surface roughness from AFM, please refer to Section S2.

In our previous work,^22^ it was demonstrated that Pt catalyzes the H_2_O-induced oxidation of H_3_PO_3_ yielding H_3_PO_4_ and H_2_, leading to a higher yield of H_3_PO_3_ oxidation on Pt surfaces with a higher surface area. To further investigate the Pt influence on the oxidation behavior of aqueous H_3_PO_3_, P *K*-edge XANES measurements of 5 mol dm^–3^ H_3_PO_3_ and H_3_PO_4_ were conducted on three different substrates: bare Kapton (a “Pt free” substrate), a 15 nm thick flat planar Pt with a low surface area (deposited on Kapton), and a rough ∼10 nm thick Pt black with a higher surface area (deposited on the 15 nm thick planar Pt electrode). The deliberate increase of Pt availability, surface area, and thickness enables a systematic analysis of the Pt influence on the chemical properties of the aqueous electrolyte. In these experiments, the potential of both of the Pt electrodes (planar Pt and Pt black) was allowed to settle at the OCP. The recorded XANES spectra and the schematic illustration of the experimental setup for these measurements are presented in Figure 1.

As depicted in Figure 1B, the XANES spectra of 5 mol dm^–3^ H_3_PO_4_ in contact with different substrates do not show any significant spectral change, indicating the stability of H_3_PO_4_ in contact with the Pt surface, in line with the result of our previous study.^22^ However, in the case of 5 mol dm^–3^ H_3_PO_3_ (Figure 1C), noticeable changes in the XANES spectra were observed for the different substrates. All of the spectra are dominated by two main features at excitation energies of ≈2150.7 and 2152.5 eV, attributed to the white line of P compounds with an oxidation state of (+3) and (+5), respectively. Yet, the spectrum recorded on Pt black exhibits a particularly high spectral weight at an energy that corresponds to the white line of P compounds with an oxidation state of (+5), i.e., H_3_PO_4_-like compounds (hereafter referred to as P(+5) compounds for simplicity). This observation corroborates the results of our previous study,^22^ where a more pronounced oxidation of H_3_PO_3_ to H_3_PO_4_ was observed when in contact with a rough Pt black surface. The rougher surface of Pt black compared to the planar Pt electrode is confirmed by AFM images (Figure 1D,E). AFM revealed an estimated surface roughness of (0.9 ± 0.1) nm for planar Pt and (4.6 ± 0.3) nm for Pt black, showing that Pt black is around 5 times rougher than planar Pt. Additional H_UPD_ of both electrodes also shows that Pt black possesses around a 5 times higher ECSA than planar Pt (Figure 1F, details on H_UPD_ can be found in Section S2), in agreement with the AFM results.

Furthermore, close inspection of the XANES data, as shown in Figure 1C, reveals that the ratio of the intensity related to the features of the P(+5) white line and the P(+3) white line decreases in the order of Pt black, planar Pt, and “Pt free” Kapton. This decrease can generally be rationalized by two factors: (i) the decreasing availability of Pt (due to the reduced roughness or the deliberate omission of Pt) and (ii) the decrease of the probed electrode-surface-to-electrolyte-volume ratio. The decrease of this ratio is a result of the diminishing surface roughness and decreasing thickness of the electrodes in the order of Pt black, planar Pt, and Kapton. Particularly, the decrease of Pt thickness results in an increase of the effective detection depth in the electrolyte. Detailed considerations for the effective detection depth in the electrolyte and probed electrode-surface-to-electrolyte-volume ratio for this study are provided in Sections S9 and S10.

Moreover, the XANES spectra of 5 mol dm^–3^ H_3_PO_3_ recorded on “Pt free” Kapton intriguingly reveals a spectral fingerprint at an energy corresponding to the white line position of P(+5) compounds, even though no oxidation due to Pt is anticipated in this system. This spectral feature could be attributed either to (i) the intrinsic spectral fingerprint of H_3_PO_3_ itself, as pure H_3_PO_3_ (i.e., solid crystalline H_3_PO_3_) displays a XANES spectral feature at an energy very close to the P(+5) compounds’ white line position at 25 °C, likely arising from multiple scattering resonance (as detailed in ref (29)); (ii) the radiation-induced oxidation of aqueous H_3_PO_3_ to H_3_PO_4_ (discussed in detail in the next section); or (iii) the convolution of both effects. Additionally, both the 5 mol dm^–3^ XANES spectra recorded on planar Pt and Pt black seem to exhibit a shoulder at ≈2155 eV, although the origin of this spectral feature remains unclear.

Nevertheless, the progressive increase in the spectral weight of the 5 mol dm^–3^ H_3_PO_3_ XANES data at the excitation energy corresponding to P(+5) compounds in the order of “Pt free” Kapton, planar Pt, and Pt black clearly indicates the Pt-catalyzed oxidation of aqueous H_3_PO_3_ to H_3_PO_4_-like compounds. This result is further supported by complementary gas chromatography (GC) experiments recorded during the mixing of Pt catalysts with aqueous H_3_PO_3_. These measurements confirm the formation of H_2_ upon addition of Pt/C catalysts into aqueous H_3_PO_3_ solution, which further confirms that Pt indeed catalyzes the chemical reaction of H_3_PO_3_ + H_2_O ⇄ H_3_PO_4_ + H_2_. Details of the GC experiments are provided in Section S11, Figures S13 and S14.

### 3.2 Impact of Irradiation on the P *K-*Edge XANES of the Aqueous H_3_PO_3_ Electrolyte

**Figure 2:**
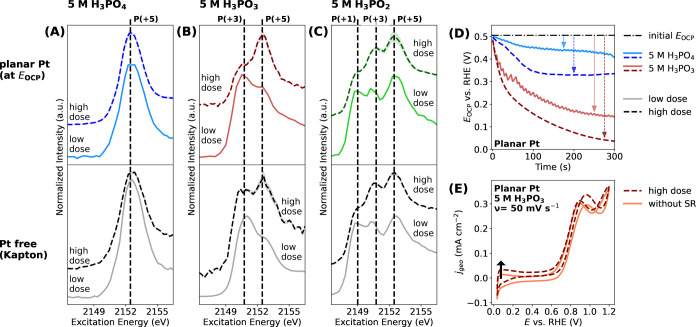
P *K-*edge XANES spectra recorded with different irradiation doses (labeled “low dose” and “high dose”) of the 5 mol dm^–3^ (5 M) electrolyte of: (A) H_3_PO_4_, (B) H_3_PO_3_, and (C) H_3_PO_2_ in contact with either (top) the planar Pt electrode or (bottom) the “Pt free” Kapton substrate. The XANES data with the planar Pt electrode are recorded at the open-circuit potential (*E*_OCP_). The average spectra of three measurements at different sample positions are represented by either solid lines (for low dose measurement) or dashed lines (for high dose measurement), with the shaded regions corresponding to the respective standard deviations of the individual measurements from the mean. The vertical dashed lines correspond to the characteristic white line positions of P-containing compounds with oxidation states of (+1), (+3), or (+5). Negligible irradiation dose-dependent spectral changes are observed in the XANES data recorded on 5 M H_3_PO_4_. However, the XANES data recorded on 5 M H_3_PO_3_ under the high dose show a pronounced increase in the spectral weight corresponding to P(+5) and a decrease corresponding to P(+3), compared to measurements with the low dose. Similarly, XANES data of 5 M H_3_PO_2_ exhibit a pronounced irradiation dose-dependent spectral shape change with increasing P(+3)- and P(+5)-related intensities, a reduction of spectral intensity ascribed to P(+1). (D) Electrode open-circuit potential (*E*_OCP_) time scans of 5 M H_3_PO_4_ and 5 M H_3_PO_3_ on the planar Pt electrode at different irradiation doses. (E) Cyclic voltammograms (CVs) for planar Pt|(5 M) H_3_PO_3_, collected without irradiation and with a high radiation dose, using a starting potential of +0.05 V_RHE_ and a scan speed of 50 mV s^–1^. Under high irradiation doses, there is a drop in the *E*_OCP_ and a shift in the CV toward higher current densities.

To ensure the reliability of the XANES results and to assess the impact of highly brilliant synchrotron radiation on the aqueous H_3_PO_3_ system, P *K*-edge XANES and electrochemical measurements (OCP and CV) were performed on 5 mol dm^–3^ H_3_PO_*x*_ (H_3_PO_4_, H_3_PO_3_, and H_3_PO_2_) electrolytes under different irradiation conditions with the planar Pt electrode and the “Pt free” Kapton substrate, as depicted in Figure 2. The use of planar Pt as the working electrode allowed for simultaneous electrochemical characterizations during irradiation while minimizing the Pt effect to facilitate a clear observation of the irradiation effect since electrochemical characterizations cannot be conducted with the nonconductive “Pt free” Kapton substrate. XANES measurements with “Pt free” Kapton serves as a comparison to the XANES data recorded on planar Pt for clearer insights into the irradiation influence without the presence of Pt. Detailed information regarding radiation dose estimation is provided in Section S12, Table S2.

As depicted in Figure 2A, the XANES spectra of 5 mol dm^–3^ H_3_PO_4_ recorded on both “Pt free” Kapton (bottom panel) and planar Pt (top panel) did not show any significant spectral change, regardless of the irradiation dose applied. This indicates that H_3_PO_4_ is stable even under intense radiation. However, in the case of 5 mol dm^–3^ H_3_PO_3_ on both “Pt free” Kapton and planar Pt (Figure 2B), the XANES spectra recorded under a high dose exhibited a substantial increase in spectral weight corresponding to P(+5) compounds, compared to the spectra obtained with a low dose. Additional XANES experiments of aqueous H_3_PO_3_ performed under several different doses confirm the same trends (see Section S13, Figure S17). This phenomenon suggests that the highly brilliant irradiation induces the oxidation of H_3_PO_3_ to form H_3_PO_4_-like compounds.

Furthermore, the irradiation also has a notable impact on the electrochemical behavior of 5 mol dm^–3^ H_3_PO_3_, as evidenced from the *E*_OCP_ time scans (Figure 2D) and CVs (Figure 2E). Upon exposure to irradiation, there was a noticeable drop in *E*_OCP_ values, and the magnitude of this drop increased with increasing irradiation dose. Additional *E*_OCP_ recordings made under different radiation doses confirm the trend of *E*_OCP_ value drops (see Section S14, Figure S18). Similarly, the CVs recorded under irradiation were shifted to more positive current densities compared to the CVs measured without irradiation. The observed *E*_OCP_ and CV responses suggest the presence of H_2_ induced by the irradiation in the proximity of the Pt electrode. If H_2_ is present in the vicinity of the Pt surface, the equilibrium reaction of 2 H^+^+ 2 e^–^ ⇆ H_2_ will take place and lower the *E*_OCP_ to less positive potentials, closer to the potential of the reversible hydrogen electrode (i.e., *E*_RHE_ = 0 V). After a prolonged time of irradiation, the concentration of H_2_ in the electrode vicinity achieves a steady value. This is a result of a steady state between the rate of H_2_ generation and the rate of its diffusion from the electrode surface. A theoretical estimation based on the *E*_OCP_ recording shows that the H_2_ concentration in 5 mol dm^–3^ H_3_PO_3_ under the high irradiation dose is indeed considerably larger than under the low irradiation dose (by approximately a factor of 10^3^). Details for the estimated H_2_ concentration generated in these experiments can be found in Section S15, Table S3. This interpretation is further supported by the oscillations in the *E*_OCP_ signal during experiments with low irradiation doses. The rapid opening and closing of the beam-blocking valve during low irradiation dose measurements result in an intermittent H_2_ generation when the sample is irradiated (i.e., when the valve is opened) and cessation of H_2_ formation when the radiation is blocked (i.e., when the valve is closed). Consequently, there is a periodic decrease of *E*_OCP_ when H_2_ is generated and a periodic increase in *E*_OCP_ when H_2_ generation ceases, as the H_2_ previously generated during irradiation diffuses away from the Pt electrode. In the high dose measurement, such oscillations of the *E*_OCP_ signal are not observed since in this case, the sample is continuously irradiated. Additional *E*_OCP_ recordings over an extended period of irradiation and without irradiation confirm the same trend (detailed in Section S14, Figure S19). Furthermore, the presence of easily oxidizable species (such as H_2_) is also apparent in the CV, where it causes a shift of currents to more positive values.

The combined observations from XANES and electrochemical analysis suggest that under intense irradiation, a pronounced oxidation of H_3_PO_3_ takes place, resulting in the generation of H_3_PO_4_-like compounds and H_2_. Since aqueous electrolytes were used in the study, two plausible mechanisms arise for the radiation-induced oxidation of H_3_PO_3_ by H_2_O. The first possibility is that the radiation induces the radiolysis of H_2_O, leading to the formation of hydrogen radicals (H^•^) and hydroxyl radicals (HO^•^). These hydroxyl radicals are strong oxidizing agents capable of oxidizing H_3_PO_3_ to H_3_PO_4_, resulting in the subsequent formation of additional hydrogen radicals. The remaining H^•^ can then recombine to form H_2_, as shown in eq 1. The generation of H^•^ and HO^•^ due to the radiolysis of water has been extensively documented in previous studies.^38,39^ In particular, soft X-ray synchrotron irradiation with photon energies between 200 eV and 2 keV has been shown to induce H_2_O radiolysis, resulting in products such as hydroxyl radicals.^39^ Another possible mechanism is that the radiation may excite H_3_PO_3_ (denoted as H_3_PO_3_* in the following discussion), making it more susceptible to undergo tautomerization to “active” pyramidal H_3_PO_3_. The pyramidal H_3_PO_3_ tautomer is more prone to react with H_2_O to form H_3_PO_4_ and H_2_, as shown in eqs 2A and 2B. Moreover, both suggested mechanisms can occur in parallel, or even in a cooperative manner, for instance HO^•^ can easily react with H_3_PO_3_* to form H_3_PO_4_ and H_2_ in a reaction similar to eq 1. These interpretations are further supported by XANES measurements of 5 mol dm^–3^ H_3_PO_3_ on “Pt free” bare Kapton under different irradiation doses (the bottom panel of Figure 2B), which exhibit similar trends to the XANES measurements recorded on planar Pt (the top panel of Figure 2B). This indicates that radiation-induced oxidation occurs even in the absence of Pt, reinforcing the notion that the process likely proceeds via interactions with H_2_O, as previously discussed.
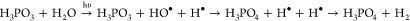
1
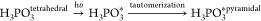
2A
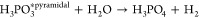
2B

To investigate whether the H_2_ formation is solely due to the radiation-induced oxidation of H_3_PO_3_, as discussed earlier, or if other processes were contributing to the generation of H_2_, the XANES and *E*_OCP_ recording of planar Pt in contact with 5 mol dm^–3^ H_3_PO_4_ under different irradiation doses can be examined. As previously discussed, the P *K-*edge XANES spectra of aqueous H_3_PO_4_ recorded using different radiation doses barely show any spectral change, suggesting its high stability under intense irradiation (Figure 2A). However, the *E*_OCP_ of aqueous H_3_PO_4_ also exhibited a drop under irradiation, and this effect increased with an increasing radiation dose (Figure 2D). This indicates that other irradiation-induced processes are taking place during the measurement of aqueous H_3_PO_4_, leading to the formation of reducing species such as H_2_. The irradiation likely induces the radiolysis of H_2_O, resulting in the direct generation of H_2_, as well as HO^•^ and H^•^ radicals.^38,39^ These H^•^ radicals can react with one another, resulting in H_2_ formation. Additionally, the radiolysis of H_3_PO_4_ may also lead to the formation of phosphoric acid radicals (H_2_PO_4_^•^), protons (H^+^), and electrons (e^–^), as previously reported by ref (41). The H^+^ and e^–^ might recombine, leading to the formation of the hydrogen atom (H). Subsequently, the hydrogen atom might react with H^•^, resulting in H_2_ generation. Therefore, the decrease in *E*_OCP_ in the case of XANES probing of Pt|aqueous H_3_PO_4_ may be correlated to the formation of H_2_ resulting from the radiolysis of H_2_O and H_3_PO_4_.

However, it is important to note that the *E*_OCP_ drop for the Pt|(5 mol dm^–3^)H_3_PO_4_ sample is substantially lower compared to the drop observed for the Pt|(5 mol dm^–3^)H_3_PO_3_ system. Assuming that the *E*_OCP_ drop is solely caused by H_2_ presence, this implies that the H_2_ generation in aqueous H_3_PO_3_ is much more intense than in the aqueous H_3_PO_4_. It is reasonable to assume that the magnitude of the H_2_O radiolysis-induced *E*_OCP_ drop is the same for 5 mol dm^–3^ H_3_PO_3_ and 5 mol dm^–3^ H_3_PO_4_, given that the H_2_O concentration is similar in both solutions. Therefore, the irradiation-induced *E*_OCP_ drop for Pt|5 mol dm^–3^ H_3_PO_3_ was caused by at least two processes: (i) the formation of H_2_ by H_2_O radiolysis previously discussed and (ii) the generation of H_2_ via the radiation-induced oxidation of H_3_PO_3_ (as shown in eq 1 and/or eq 2A, 2B).

Additional P *K-*edge XANES measurements of 5 mol dm^–3^ H_3_PO_2_, a P-containing acid with a nominal phosphorus oxidation state of (+1), were performed with both planar Pt and “Pt free” Kapton. For both electrodes, under an intense radiation dose, there is an increase in the spectral weight corresponding to P compounds with oxidation states of (+3) and (+5) compared to the respective spectra measured with a low radiation dose (see Figure 2C). Similar to aqueous H_3_PO_3_, the aqueous solution of H_3_PO_2_ in H_2_O is considered thermodynamically unstable^44^ and it is likely that the intense radiation also induces the oxidation of H_3_PO_2_ by H_2_O, as described in eqs 3 and 4A, 4B. Moreover, the formed H_3_PO_3_ may undergo further oxidation to H_3_PO_4_ through reactions given in eqs 1 and 2A, 2B.
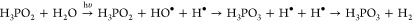
3
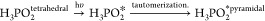
4A
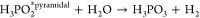
4B

Complementary IEC experiments made on aqueous H_3_PO_2_ confirm that in the presence of Pt, H_3_PO_2_ converts to H_3_PO_4_ faster than H_3_PO_3_ (see Figure S21 and Table S4). The absence of any possible radiation-induced oxidation in the IEC measurements seems to indicate that alternative mechanisms/pathways of H_3_PO_2_ oxidation to H_3_PO_4_ via H_2_O exist, obviating the formation of tetrahedral H_3_PO_3_. This could explain the increased spectral weight of P(+3) and P(+5) signals at high-dose XANES as well as the pronounced conversion of aqueous H_3_PO_2_ to H_3_PO_4_ observed by IEC. Further experiments beyond the scope of the present study are required to elucidate the mechanism of aqueous H_3_PO_2_ oxidation, although these findings already show that both aqueous P-containing acids (H_3_PO_2_ and H_3_PO_3_) are thermodynamically unstable and tend to form the more stable H_3_PO_4_ in the presence of Pt and/or upon intense irradiation.

Finally, it is crucial to highlight that the P *K-*edge XANES data of planar Pt|(5 mol dm^–3^) H_3_PO_3_ recorded with a low radiation dose exhibit a small standard deviation over multiple measurements, as demonstrated in Figure 2B (see shaded regions corresponding to the respective standard deviations of the individual measurements from the mean). This observation suggests that our approach of minimizing the radiation dose is effective in sufficiently suppressing undesirable radiation-induced effects. Therefore, for the remainder of this work, all presented P *K-*edge XANES spectra are collected with the low irradiation dose using the experimental protocol to minimize radiation-induced effects, as described in detail in Section 2.2.2. This ensures that any changes in spectral features primarily occur due to deliberate variations of targeted experimental conditions, such as Pt surface roughness, potential bias, or temperatures, and not as a result of undesired radiation-induced phenomena. In fact, the P *K-*edge XANES measurements presented in Figure 1 were recorded using low irradiation doses. Similarly, our previous studies using XANES^29^ and AP-HAXPES^22^ were performed at beamlines with comparatively lower irradiation doses (i.e., lower photon fluxes) compared to the current measurement, as detailed in Section S12.

### 3.3 Electrode Potential-Dependent Oxidation of Aqueous H_3_PO_3_ on Pt

**Figure 3:**
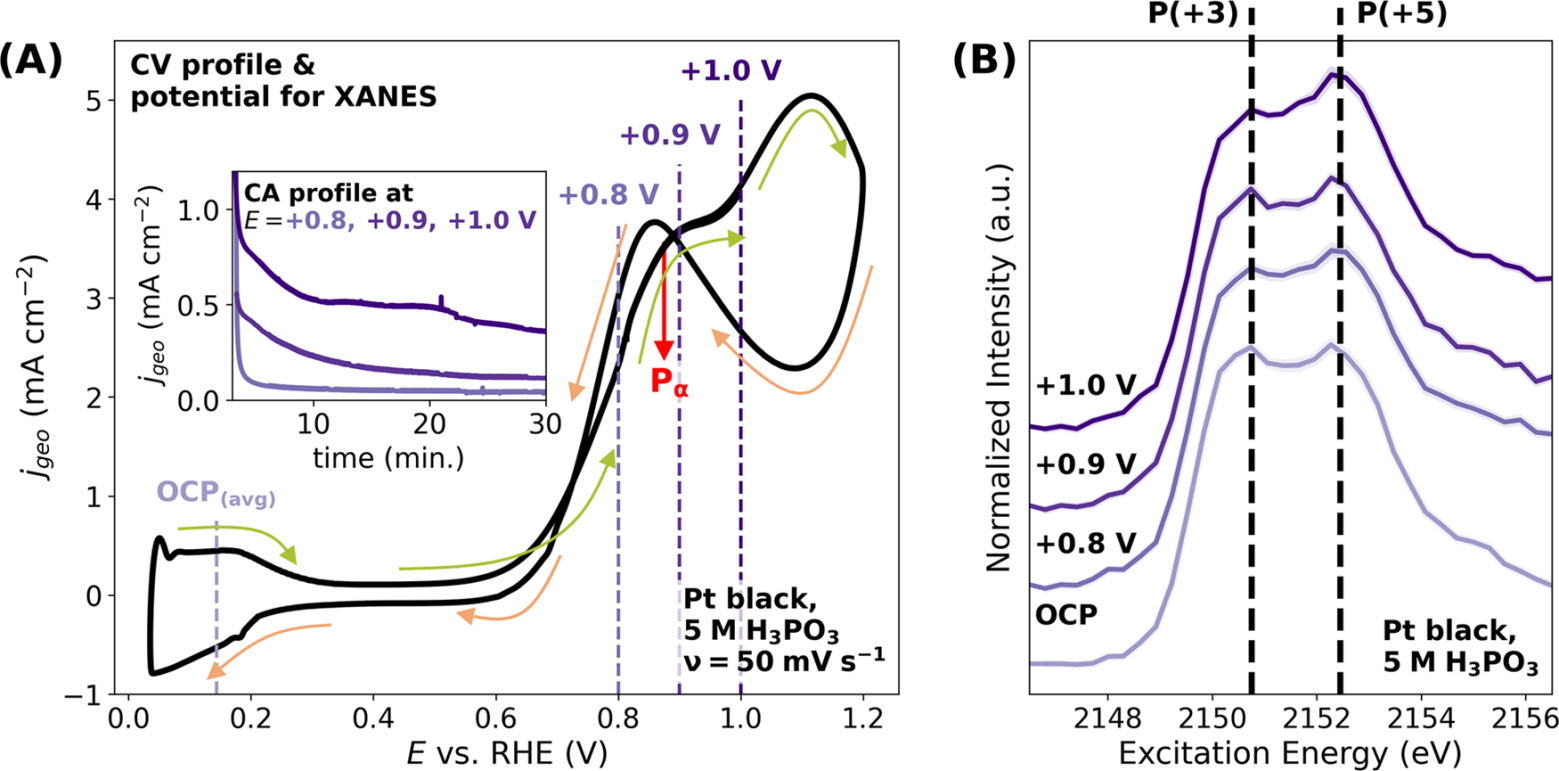
(A) CV obtained during the *in situ* experiment with the Pt black electrode at 25 °C using the 5 mol dm^–3^ (5 M) H_3_PO_3_ electrolyte. The dashed lines indicate the selected potentials at which *in situ* P *K*-edge XANES spectra were recorded. Pα denotes the maximum current density of the electrochemical oxidation of H_3_PO_3_ to H_3_PO_4_ via eq 5. The green arrows illustrate the current density response during the positive-going potential sweep, while the orange arrows show the current density response during the negative-going potential sweep. The CV recording started at the potential of +0.05 V_RHE_ with the positive-going potential sweep, using a scan rate of 50 mV s^–1^. The inset graph shows the chronoamperometry (CA) current density profiles during the potential bias at +0.8 V_RHE_, +0.9 V_RHE_, and +1.0 V_RHE_. (B) *In situ* P *K*-edge XANES spectra of Pt black electrodes with 5 mol dm^–3^ (5 M) H_3_PO_3_ at the OCP and at positive potentials (at 25 °C). The solid curves represent the average spectra of three measurements at different sample positions, with the shaded regions corresponding to the respective standard deviations of the individual measurements from the mean. A slight increase in the spectral weight corresponding to P compounds with an oxidation state of (+5) is observed upon the application of a more positive potential bias.

To investigate the electrode potential-induced anodic oxidation of aqueous H_3_PO_3_, *in situ* P *K-*edge XANES spectra were acquired at *E*_OCP_ and at more positive potentials, where H_3_PO_3_ is expected to undergo electrochemical oxidation. These measurements were performed using a Pt black electrode and 5 mol dm^–3^ H_3_PO_3_. The use of the Pt black electrode was preferred due to (i) its larger electrochemically active surface area compared to the planar Pt electrode, which leads to a higher yield of reaction products during the application of potential bias and (ii) a higher ratio of probed electrode-surface-to-electrolyte-volume compared to the planar Pt electrode. The following Figure 3 displays the CV, P *K*-edge XANES spectra measured at various electrode potentials, and the corresponding CA current profiles.

As shown in Figure 3A, the CV of the Pt black electrode in 5 mol dm^–3^ H_3_PO_3_ is presented, along with the electrode potential used for the *in situ* P *K-*edge XANES measurements. The increase in current densities observed during the positive-going potential sweep (around +0.7 V_RHE_) corresponds to the anodic oxidation of H_3_PO_3_ (eq 5), with the maximum current density from this reaction observed at the peak Pα. Further details that this peak likely corresponds to the electrochemical oxidation of H_3_PO_3_ to H_3_PO_4_ via eq 5 can be found in refs (18,19,22)
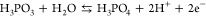
5

The respective *in situ* XANES spectra recorded at the potentials of +0.8 V_RHE_ and higher display a slight increase of the intensity ratio related to the feature at the P(+5) white line to the feature at the P(+3) white line upon increasing potentials compared to the spectra recorded at the *E*_OCP_ (see Figure 3B). This increase in the intensity ratio corresponds to the formation of H_3_PO_3_ oxidation products, as evidenced by the increase in the current density drawn by the Pt black electrode with the increasing potential (see the inset graph of Figure 3A). Theoretical estimations further support that the amount of generated oxidation products indeed increases with the increasing potential, as detailed in Section S17, Figure S22. However, the *in situ* XANES spectral changes are small, mainly because the P *K-*edge XANES data contain a convolution of signals arising from both the Pt|H_3_PO_3_ interface and (mainly) the bulk electrolyte. Consequently, a similar experiment performed with a planar Pt electrode shows a negligible spectral change upon increasing the potential, as there is even less Pt surface available for the electrochemical oxidation process, resulting in a significantly smaller yield of oxidation products and a considerably smaller probed electrode-surface-to-electrolyte-volume ratio (as detailed in Sections S18 and S10, Figure S23).

### 3.4 Thermal Stability of Aqueous H_3_PO_3_

**Figure 4:**
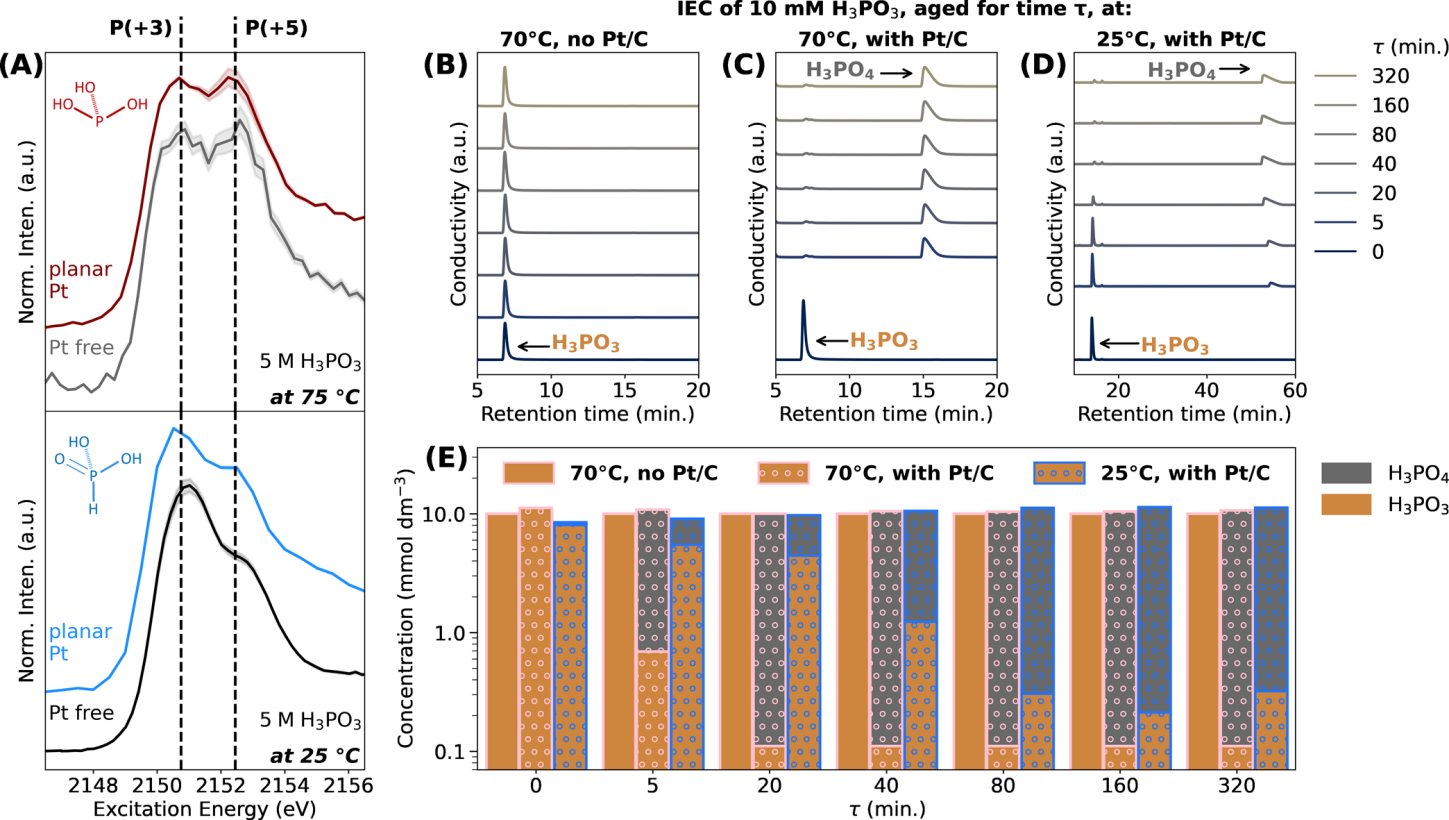
(A) *In situ* P *K*-edge XANES spectra of “Pt free” bare Kapton and planar Pt in contact with 5 mol dm^–3^ (5 M) H_3_PO_3_ at (top) 75 °C and (bottom) 25 °C, both measured at the OCP. The 2D chemical structures in the graph correspond to the two tautomeric forms of H_3_PO_3_: the pyramidal form (at 75 °C) and the tetrahedral form (at 25 °C). The solid curves represent the average spectra of three measurements at different sample positions, with the shaded regions corresponding to the respective standard deviations of the individual measurements from the mean. An increase in the spectral weight corresponding to P compounds with the oxidation state of (+5) is observed in the XANES recorded at higher temperatures (both “Pt free” Kapton and planar Pt) compared to the lower temperature. At the higher temperature, negligible spectral change is observed between Kapton and planar Pt. (B–D) IEC performed on a 10 mmol dm^–3^ (10 mM) H_3_PO_3_ electrolyte that has been aged for a specific duration (τ) at 70 °C [for panels (B) and (C)] or 25 °C [for panel (D)]. In panel (B), no Pt/C catalysts were dispersed in the electrolyte before aging, while in panels (C) and (D), 50 mg of Pt/C catalysts (40 wt % Pt) were dispersed before aging. Note that the eluent solution in panels (B) and (C) was 20 mmol dm^–3^ KOH, while the eluent in panel (D) was 15 mmol dm^–3^ KOH; therefore, a different retention time is observed for H_3_PO_3_/H_3_PO_4_ species in panels (B) and (C) compared to panel (D). (E) The concentration of H_3_PO_3_ and H_3_PO_4_, derived from IEC (panels (B–D)). Without Pt/C dispersion (the bar plot without dots), the aqueous H_3_PO_3_ electrolyte remains stable, but upon dispersion of Pt/C catalysts (the dotted bar plot), H_3_PO_3_ immediately undergoes oxidation to H_3_PO_4_. A higher conversion rate from H_3_PO_3_ to H_3_PO_4_ is observed at a higher temperature.

Figure 4A displays the *in situ* P *K-*edge XANES spectra of the 5 mol dm^–3^ H_3_PO_3_ electrolyte recorded at two different temperatures: 25 and 75 °C, using “Pt free” bare Kapton and a planar Pt electrode. The use of planar electrodes minimizes the catalytic contribution of the Pt surface to the spectra, enabling a clearer observation of the temperature effect. “Pt free” bare Kapton serves as a comparison for the XANES measurements with planar Pt.

As shown in Figure 4A, the P *K-*edge XANES spectra of both Kapton and planar Pt recorded at 75 °C exhibit a higher spectral weight corresponding to P(+5) compounds (i.e., H_3_PO_4_-like compounds) compared to measurements performed at 25 °C. This indicates that a higher temperature facilitates the oxidation of H_3_PO_3_ to H_3_PO_4_-like compounds. To further investigate the nature of this oxidation process at a high temperature, ion-exchange chromatography (IEC) experiments were conducted on a 10 mM aqueous H_3_PO_3_ solution that has been aged at 75 °C, both with and without the presence of Pt/C.

The IEC analysis of the “Pt free” aqueous H_3_PO_3_ solution aged at 70 °C revealed only the presence of H_3_PO_3_ (see Figure 4B), indicating that at an elevated temperature without Pt, the solution remains stable during the time frame of the experiment. On the other hand, the IEC analysis of the aqueous H_3_PO_3_ solution with Pt/C dispersion showed a conversion of a majority of H_3_PO_3_ to H_3_PO_4_ after only ∼5 min of aging at 70 °C (see Figure 4C). Furthermore, the oxidation rate of H_3_PO_3_ to H_3_PO_4_ in the presence of Pt is notably faster at 75 °C compared to 25 °C (see Figure 4D,E), indicating that the elevated temperature indeed enhances the oxidation rate of H_3_PO_3_ to H_3_PO_4_ in the presence of Pt.

However, it might seem intriguing that the XANES measurement of “Pt free” aqueous H_3_PO_3_ on bare Kapton at a high temperature also displays an increase in the spectral weight corresponding to P(+5) compounds, compared to XANES measured at 25 °C (see Figure 4A). This seems counterintuitive, given that the IEC analysis of “Pt free” aqueous H_3_PO_3_ suggests stability under these conditions. This observation may be explained by the combined effects of incoming radiation and elevated temperature, influencing the tautomeric equilibrium between tetrahedral (more stable and less active) and pyramidal (less stable and more reactive) H_3_PO_3_. As previously suggested, irradiation exposure might excite H_3_PO_3_, making it more prone to undergo tautomerization to the “active” pyramidal form, as depicted in eqs 2A and 2B. However, our approach of minimizing irradiation doses has effectively suppressed this phenomenon so that there is minimal influence from this phenomenon to the spectral change. Yet, at elevated temperatures, heat provides additional energy to H_3_PO_3_ and makes the tautomerization more energetically favorable. Therefore, at elevated temperatures during irradiation, H_3_PO_3_ is more inclined to undergo tautomerization from the less active tetrahedral to the more active pyramidal form. As a result, at 75 °C, a larger proportion of H_3_PO_3_ exists in the otherwise less thermodynamically favored yet “active” pyramidal form, as compared to 25 °C. The shift in the tautomeric equilibrium at higher temperatures has also been suggested in previous works^47,48^ (albeit without irradiation). Since the “active” pyramidal form is more prone to react with H_2_O in the solution, H_3_PO_3_ undergoes oxidation to H_3_PO_4_. Additionally, the H_3_PO_3_ oxidation reaction proposed in eq 1 might be more pronounced at elevated temperatures since the probability for HO^•^ and H^•^ to recombine before reacting with H_3_PO_3_ is lower at elevated temperatures compared to room temperature (due to the enhanced diffusion rate at a higher temperature). Thus, the combined effect of heat and radiation induces the oxidation of aqueous H_3_PO_3_ to H_3_PO_4_, as indicated in eq 6.
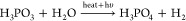
6

Furthermore, the combined heat and radiation effect is bigger than the catalytic effect of the planar Pt surface. Therefore, no significant spectral change is observed between XANES of planar Pt and Kapton at this elevated temperature (see Figure 4A). Additional XANES measurement of planar Pt under a positive potential bias at this elevated temperature further supports this observation: the recorded spectra display negligible change upon applications of positive potentials, even though a high current density was drawn to the working electrode at this temperature (please see the discussion in Section S19, Figure S24).

The convolution of different effects, including temperature, radiation, and Pt influences, illustrates the complexity of *in situ* XANES under these experimental conditions. To fully elucidate the individual effects arising from each experimental parameter, complementary experimental techniques beyond XANES are required.

### 3.5 Impact of H_2_O Concentration on the Oxidation of Aqueous H_3_PO_3_

In the previous section, various oxidation mechanisms of aqueous H_3_PO_3_ to H_3_PO_4_ were discussed, including Pt-catalyzed oxidation, radiation-induced oxidation, electrochemical oxidation under a positive potential bias, and the combined heat and radiation effect. In addition, IEC reveals that in the presence of Pt, an increased temperature enhances the oxidation of aqueous H_3_PO_3_ to H_3_PO_4_. All of these oxidation processes involve H_2_O, which acts as a reactant (a source of oxygen atoms), as documented in eqs 1, 2A, 2B, 5, and 6. Given the significant role of H_2_O in different oxidation mechanisms of H_3_PO_3_, additional P *K-*edge XANES measurements of different aqueous H_3_PO_3_ concentrations, ergo different concentrations of H_2_O, were performed to gain further insight into its influence on H_3_PO_3_ oxidation.

**Figure 5:**
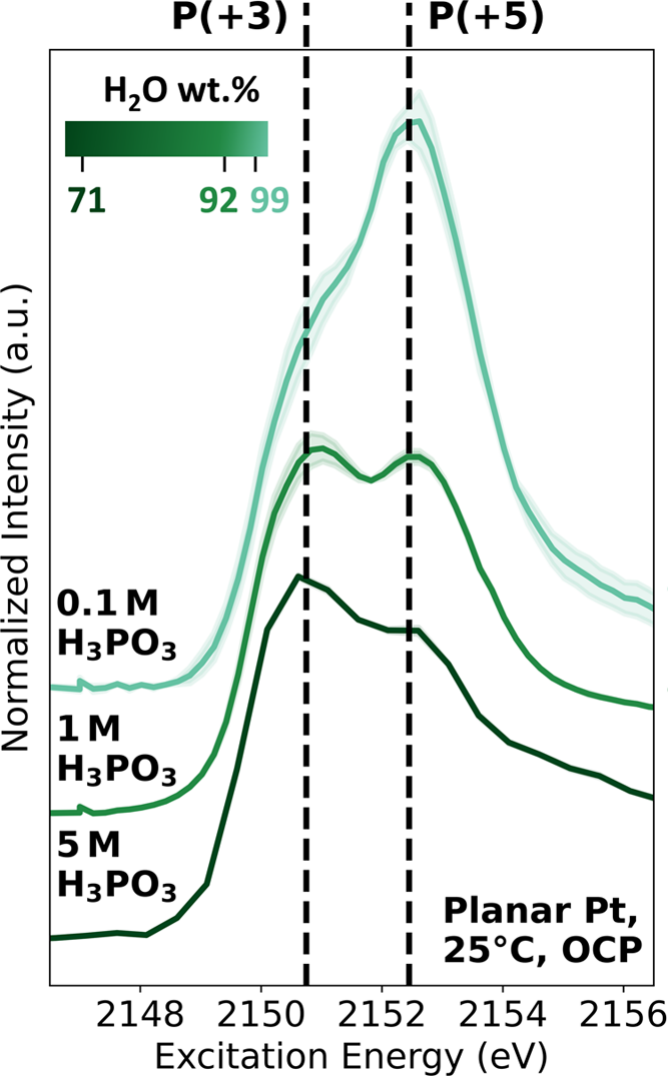
P *K*-edge XANES of the planar Pt/aqueous H_3_PO_3_ electrolyte with different concentrations: 0.1 mol dm^–3^ (0.1 M), 1 mol dm^–3^ (1 M), and 5 mol dm^–3^ (5 M). The solid curves represent the average spectra of three measurements at different sample positions, with the shaded regions corresponding to the respective standard deviations of the individual measurements from the mean. A higher spectral weight in the region corresponding to P(+5) compounds is observed for electrolytes with a high content of H_2_O (i.e., H_2_O wt %).

Figure 5 clearly demonstrates that the P *K-*edge XANES spectra of aqueous H_3_PO_3_ electrolytes with a lower H_3_PO_3_ molar concentration exhibit a stronger spectral weight corresponding to P(+5)-compounds. This confirms that more pronounced H_3_PO_3_ oxidation occurs in an electrolyte with a higher content of H_2_O (i.e., higher H_2_O wt %). The same trend is also observed in XANES measurements performed with a high irradiation dose, as shown in Figure S25. Additionally, the difference in the white line intensity relative to the edge jump (i.e., at 2156.5 eV) for electrolytes of different concentrations is likely caused by the self-absorption effect occurring in FY-XANES measurements. A similar observation was reported in ref (29), wherein a solution with a higher concentration exhibited a lower white line intensity relative to the edge jump compared to a solution of a lower concentration. Nevertheless, this effect does not influence other trends observed in this measurement, such as the increase of the spectral weight corresponding to P(+5) compounds in electrolytes with a higher H_2_O content.

This finding validates the overall reaction equation formulated for the different oxidation mechanisms of H_3_PO_3_ in the presence and absence of Pt electrodes. This highlights the significant impact of H_2_O as a reactant on the oxidation of aqueous H_3_PO_3_, as it influences the reaction rate of H_3_PO_3_ oxidation.

## 4 Summary and Conclusions

Expanding on previous findings revealing that aqueous H_3_PO_3_ oxidizes in contact with Pt, we have investigated the various complex oxidation mechanisms of aqueous H_3_PO_3_ to H_3_PO_4_ in different experimental conditions by *in situ* tender XANES at the P *K*-edge. This technique provides further evidence that Pt catalyzes the oxidation of aqueous H_3_PO_3_ to H_3_PO_4_. We have also shown that a more pronounced oxidation of aqueous H_3_PO_3_ occurs in electrolytes with a higher content of H_2_O. With the aim of probing the H_3_PO_3_ oxidation behavior at conditions more relevant for HT-PEMFC operation, *in situ* XANES measurements during the application of a positive potential bias of +0.8 V_RHE_ and higher suggest the electrochemical oxidation of aqueous H_3_PO_3_ to H_3_PO_4_. Furthermore, the combination of *in situ* XANES and complementary IEC at elevated temperatures reveals that heat enhances the oxidation of aqueous H_3_PO_3_. Additionally, *in situ* XANES and electrochemical characterizations performed under different irradiation doses indicate that the intense radiation-induced oxidation of H_3_PO_3_ via H_2_O results in the formation of H_3_PO_4_ and H_2_. A broadly applicable experimental procedure was implemented to minimize the undesirable effects of radiation. This effect shows the need for careful consideration of X-ray irradiation-induced effects for future *operando* investigations of HT-PEMFCs with tender X-rays.

This work sheds light on the complex oxidation mechanism of aqueous H_3_PO_3_ to H_3_PO_4_. It highlights the significant role of H_2_O in oxidizing H_3_PO_3_ to the more stable H_3_PO_4_. This finding should be considered in the investigation of electrified Pt|H_3_PO_4_ interfaces (e.g., *operando* HT-PEMFC studies), where H_3_PO_3_ was indicated to be generated. Moreover, our results also provide insights into possible adjustments on the HT-PEMFC operation condition, such that the detrimental effect of H_3_PO_3_ formation could be mitigated, e.g., through control of humidification to oxidize the formed H_3_PO_3_ back to H_3_PO_4_ and/or by avoiding conditions where H_3_PO_4_ dries out excessively (e.g., conditions in which a low amount of H_2_O is generated, which may prevent H_3_PO_3_ to be oxidized back to H_3_PO_4_), such as (i) operation at low current loads close to the open-circuit voltage and (ii) high gas flow rates. Further electrochemical investigations of HT-PEMFC operation with humidification control are required to assess the feasibility and impact of this approach on the HT-PEMFC performance, but they lie outside the scope of this work.

For further insights into the Pt–H_3_PO_*x*_ interaction, similar *in situ* experiments using more surface-sensitive techniques are needed, such as near-edge X-ray absorption fine structure spectroscopy (NEXAFS) at the P *L*_2,3_-edge, which would increase the surface sensitivity by approximately 2 orders of magnitude. However, this lies beyond the scope of the current study and will be explored prospectively in the future.

## Data Availability

The data presented in this work are available at the following link: 10.5281/zenodo.10636986.

## Supporting Information Available

Current and potential profile for the electrodeposition of Pt black and estimation of Pt black maximum thickness; SEM images and electrochemically active surface area (ECSA) determination of the planar Pt electrode and the Pt black electrode and comparison with commercial Pt/C catalysts; setup of the three-electrode flow cell for the *in situ* P *K*-edge XANES investigation; comparison between XANES spectra of 5 mol dm^–3^ H_3_PO_3_ recorded with the rapid beam-blocking method and with a radiation-attenuating filter; fluorescence grid map of 5 mol dm^–3^ H_3_PO_3_ on Pt electrodes and sequential XANES measurement positions; considerations for the energy resolution of the *in situ* P *K*-edge XANES measurements; sequential P *K*-edge XANES of planar Pt|5 mol dm^–3^ H_3_PO_3_ without an electrolyte flow at different measurement positions; temperature control and monitoring during *in situ* XANES measurement; considerations for effective detection depth in the electrolyte for the *in situ* P *K*-edge XANES experiments; theoretical estimation of the probed electrode-surface-to-electrolyte-volume ratio for the P *K*-edge XANES experiments; detection of H_2_ upon the oxidation of aqueous H_3_PO_3_ to H_3_PO_4_; estimation of the irradiation dose absorbed by the electrolyte during the XANES experiments; additional P *K*-edge XANES of aqueous H_3_PO_3_ with different incoming photon fluxes; additional *E*_OCP_ recording of planar Pt|(5 mol dm^–3^) H_3_PO_3_ under synchrotron irradiation with varying incoming photon fluxes and *E*_OCP_ under repeated application of irradiation and without irradiation; *E*_OCP_ drop and H_2_ partial pressure estimation from *E*_OCP_ recordings of planar Pt|(5 mol dm^–3^) H_3_PO_3_ and planar Pt|(5 mol dm^–3^) H_3_PO_4_ under different irradiation doses; stability assessment of aqueous H_3_PO_2_ with and without the presence of Pt through ion-exchange chromatography; theoretical estimation of H_3_PO_4_ generation in 5 mol dm^–3^ H_3_PO_3_ during positive potential application on the Pt black electrodes; P *K*-edge XANES of 5 mol dm^–3^ H_3_PO_3_ on the planar Pt electrode, alongside the CV and CA profiles during the XANES measurements; P *K*-edge XANES of 5 mol dm^–3^ H_3_PO_3_ on the planar Pt electrode at 75 °C, under application of a positive potential bias; and P *K*-edge XANES of aqueous H_3_PO_3_ solutions of different concentrations on planar Pt under high radiation doses (PDF)
