# Supplementary material for: Elucidating the Complex Oxidation Behavior of Aqueous H3PO3 on Pt Electrodes via In Situ Tender X-ray Absorption Near-Edge Structure Spectroscopy at the P K-Edge
Source: J Am Chem Soc. 2024 Mar 9;146(11):7386–99. doi: 10.1021/jacs.3c12381 (PMC10958492; doi:10.1021/jacs.3c12381)
Supplement: Supplementary file 1 — ja3c12381_si_001.pdf [file ja3c12381_si_001.pdf]

**Supporting Information**

# Elucidating the Complex Oxidation Behavior of Aqueous $H_3PO_3$ on Pt Electrodes via *In Situ* Tender X-ray Absorption Near Edge Structure Spectroscopy at the P *K*-edge

Romualdus Enggar Wibowo*[1], Raul Garcia-Diez[1], Tomas Bystron[2], Marianne van der Merwe[1], Martin Prokop[2], Mauricio D. Arce[1,3], Anna Efimenko[1,4], Alexander Steigert[5], Milan Bernauer[2], Regan G. Wilks[1,4], Karel Bouzek[2], Marcus Bär*[1,4,6,7]

[1]*Department of Interface Design, Helmholtz-Zentrum Berlin für Materialien und Energie GmbH (HZB), Albert-Einstein-Str. 15, 12489. Berlin, Germany.*

[2]*Department of Inorganic Technology, University of Chemistry and Technology Prague. Technicka 5, Prague 6, 166 28, Czech Republic*

[3]*Departamento Caracterización de Materiales, INN-CNEA-CONICET, Centro Atómico Bariloche, Av. Bustillo 9500, S. C. de Bariloche, Rio Negro, 8400, Argentina*

[4]*Energy Materials In-situ Laboratory Berlin (EMIL), HZB, Albert-Einstein Str. 15, 12489. Berlin, Germany*

[5]*Institute of Nanospectroscopy, Helmholtz-Zentrum Berlin für Materialien und Energie GmbH (HZB), Albert-Einstein-Str. 15, 12489. Berlin, Germany*

[6]*Department of Chemistry and Pharmacy, Friedrich-Alexander-Universität Erlangen-Nürnberg. Egerlandstr. 3, 91058. Erlangen, Germany*

[7]*Department of X-ray Spectroscopy at Interfaces of Thin Films, Helmholtz Institute Erlangen-Nürnberg for Renewable Energy (HI ERN), Albert-Einstein-Str. 15, 12489 Berlin, Germany*

Email: enggar.wibowo@helmholtz-berlin.de; marcus.baer@helmholtz-berlin.de

**Table of Contents**

1. Current and potential profile for the electrodeposition of Pt black, estimation of Pt black maximum thickness ........ S3

2. SEM images and electrochemically active surface area (ECSA) determination of planar Pt electrode and Pt black electrode, comparison with commercial Pt/C catalysts ........ S4

3. Setup of three-electrode flow cell for the *in situ* P *K*-edge XANES investigation........ S6

4. Comparison between XANES spectra of 5 mol dm$^{-3}$ $H_3PO_3$ recorded with rapid beam blocking method and with a radiation-attenuating filter ........ S7

5. Fluorescence grid map of 5 mol dm$^{-3}$ $H_3PO_3$ on Pt electrodes and sequential XANES measurement positions ........ S8

6. Considerations for the energy resolution of the *in situ* P *K*-edge XANES measurements........ S9

7. Sequential P *K*-edge XANES of planar Pt|5 mol dm$^{-3}$ $H_3PO_3$ without electrolyte flow at different measurement positions ........ S10

8. Temperature control and monitoring during *in situ* XANES measurement........ S12

9. Considerations for effective detection depth in the electrolyte for the *in situ* P *K*-edge XANES experiments ........ S13

10. Theoretical estimation of the probed electrode-surface-to-electrolyte-volume ratio for the P *K*-edge XANES experiments........ S17

11. Detection of $H_2$ upon the oxidation of aqueous $H_3PO_3$ to $H_3PO_4$........ S22

12. Estimation of irradiation dose absorbed by the electrolyte during the XANES experiments ..... S24

13. Additional P *K*-edge XANES of aqueous $H_3PO_3$ with different incoming photon fluxes ........ S31

14. Additional $E_{OCP}$ recording of planar Pt|(5 mol dm$^{-3}$) $H_3PO_3$ under synchrotron irradiation with varying incoming photon fluxes and $E_{OCP}$ under repeated application of irradiation and without irradiation ........ S32

15. $E_{OCP}$ drop and $H_2$ partial pressure estimation from $E_{OCP}$ recordings of planar Pt|(5 mol dm$^{-3}$) $H_3PO_3$ and planar Pt|(5 mol dm$^{-3}$) $H_3PO_4$ under different irradiation doses ........ S34

16. Stability assessment of aqueous $H_3PO_2$ with and without the presence of Pt through ion-exchange chromatography........ S36

17. Theoretical estimation of $H_3PO_4$ generation in 5 mol dm$^{-3}$ $H_3PO_3$ during positive potentials application on the Pt black electrodes ........ S38

18. P *K*-edge XANES of 5 mol dm$^{-3}$ $H_3PO_3$ on planar Pt electrode, alongside the CV and CA profile during the XANES measurements........ S39

19. P *K*-edge XANES of 5 mol dm$^{-3}$ $H_3PO_3$ on planar Pt electrode at 75 °C, under application of positive potential bias........ S41

20. P *K*-edge XANES of aqueous $H_3PO_3$ solutions of different concentrations on planar Pt under high radiation doses........ S42

21. References ........ S43

## 1. Current and potential profile for the electrodeposition of Pt black, estimation of Pt black maximum thickness

The current and potential profile for the electrodeposition of Pt black is shown in **Figure S1**.

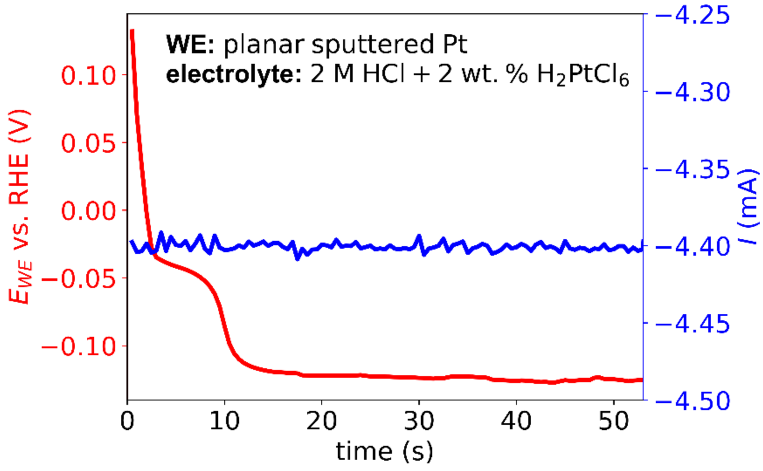


**Figure S1.** Current and potential profiles during the electrodeposition of Pt black. The electrodeposition was performed at room temperature using a solution containing 2 mol dm$^{-3}$ HCl + 2 wt% $H_2PtCl_6$.

The estimation of the electrodeposited Pt black thickness was performed using Faraday's law for electrolysis, as shown in Eq. S1:

$$d = \frac{I\, M_{\mathrm{Pt}}\, \Delta t}{\rho_{\mathrm{Pt}}\, A_{\mathrm{geo}}\, F\, z} \qquad \text{Eq. S1}$$

$I$ is the current drawn to the working electrode ($I$ ~ -4.40 mA), $M_{\mathrm{Pt}}$ is the molar mass of Pt (195 g mol$^{-1}$), $\Delta t$ is the electrodeposition time (53 s), $\rho_{\mathrm{Pt}}$ corresponds to the density of Pt (21.45 g cm$^{-3}$), $A_{\mathrm{geo}}$ is the geometrical area of the electrode in contact with the electrolyte (0.502 cm$^2$), $F$ is Faraday constant ($F$= 9.648 × 10$^4$ C mol$^{-1}$), and z is the number of electrons transferred per Pt atom in the electrodeposition process, and is equal to z = 4, for the following reaction of Pt deposition given in Eq. S2.

$$6\ H^+_{(aq)} + [PtCl_6]^{2-}_{(aq)} + 4\ e^- \leftrightarrows Pt_{(s)} + 6\ HCl_{(aq)} \qquad \text{Eq. S2}$$

From Eq. S1, it was estimated that the maximum thickness of the electrodeposited Pt black is 10 nm. It is important to note that the presented value is slightly overestimated due to the assumption of 100 % efficiency for the electrodeposition. In reality, a part of the current was used for the hydrogen evolution reaction (occurring in $E_{\mathrm{WE}} \leq 0$ V vs. RHE). The production of hydrogen on the Pt electrode surface during the electrodeposition (i.e., bubble formation on the electrode surface) facilitates a rougher formation of the electrodeposited Pt black.

## 2. SEM images and electrochemically active surface area (ECSA) determination of planar Pt electrode and Pt black electrode, comparison with commercial Pt/C catalysts

To confirm the increased surface roughness of the electrodeposited Pt black scanning electron microscopy (SEM), and hydrogen underpotential deposition ($H_{UPD}$) measurements were conducted on both the planar Pt and Pt black electrodes, as illustrated in **Figure S2**. The $H_{UPD}$ technique was employed to determine the electrochemically active surface area (ECSA) of these electrodes.

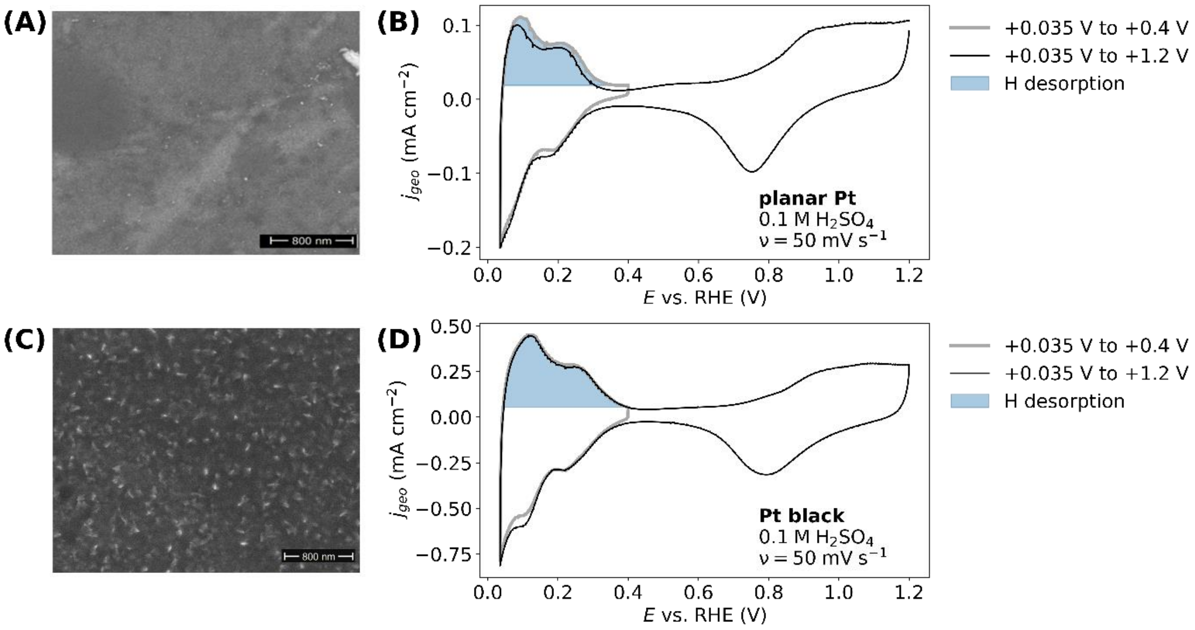


**Figure S2. (A)** SEM image and **(B)** CV of planar Pt electrode. **(C)** SEM image and **(D)** CV of Pt black electrode. SEM image on planar Pt (panel A) was taken before the *in situ* P *K*-edge XANES experiments, while the image on Pt black (panel C) was taken post-mortem after all of the *in situ* XANES experiments were performed (after the beamtime campaign). CVs were recorded for the determination of ECSA via $H_{UPD}$. CVs are recorded with the starting potential of +0.035 $V_{RHE}$ in the positive-going potential sweep in $N_2$ purged 0.1 mol $dm^{-3}$ (0.1 M) $H_2SO_4$ using the scan rate of 50 mV $s^{-1}$, with two different upper limit potentials.

SEM image of Pt black indicates an increase in surface roughness compared to planar Pt. For ECSA determination, the total charge of the underpotentially deposited hydrogen was first determined by using Eq. S3 in the following:

$$Q_{\mathrm{UPD}} = \frac{1}{\upsilon}\int_{E_{min}}^{E_{max}} I dE = \frac{A_{geo}}{\upsilon}\int_{E_{min}}^{E_{max}} j_{geo} dE \qquad \text{Eq. S3}$$

$Q_{\mathrm{UPD}}$ is the charge of the underpotentially deposited hydrogen monolayer (in C), $\upsilon$ corresponds to the scan rate (50 mV $s^{-1}$), $A_{\mathrm{geo}}$ is the geometrical area of the working electrode (0.502 $cm^2$), and the term inside the integral corresponds to the area of the shaded region in **Figure S2.B** and **S2.D**.

Subsequently, the total charge was normalized by the specific charge of the underpotentially deposited hydrogen monolayer on Pt ($\theta_{Pt}$= 210 µC cm$^{-2}$, see Ref. [1,2]). The total charge of hydrogen monolayer was found to be 149.36 µC and 750.50 µC, for planar Pt and Pt black, respectively. Using this method, the ECSA of planar Pt and Pt black was estimated to be 0.71 cm$^2$ and 3.57 cm$^2$, respectively. Subsequently, the roughness factor was determined by normalizing the ECSA with the geometrical surface area ($A_{geo}$= 0.502 cm$^2$ for both planar Pt and Pt black electrodes), yielding a roughness factor of 1.41 for planar Pt and 7.10 for Pt black. In this comparison, it is shown that Pt black exhibited approximately 5 times larger surface area than planar Pt.

To further confirm the increase in the surface roughness of the Pt electrode, AFM was performed on both electrodes (see AFM images in **Figure 1.D** and **1.E** in the main text). AFM revealed an estimated surface roughness of (0.9 ± 0.1) nm for planar Pt and (4.6 ± 0.3) nm for Pt black. The AFM-derived surface roughness supports the ECSA observation that the Pt black possesses approximately 5 times larger surface area than planar Pt.

To assess whether the Pt electrodes employed in this study exhibit similar electrochemical behavior to a commercial Pt/C catalyst commonly used in fuel cell applications, cyclic voltammetry (CV) was conducted using a catalyst ink prepared with the commercial Pt/C catalyst. For this purpose, a Pt/C catalyst ink was prepared by mixing 2.5 mg of Pt/C catalysts (HiSPEC® 4000, 40 wt.% Pt, Johnson-Matthey) with 30 µl of an isopropanol solution containing 5 wt.% nafion (LIQUion(™) Solution LQ-1005, 1000 EW @ 5% weight). The solution was stirred and sonicated for approximately 20 minutes, following the procedure outlined in Ref. [3]. Then 15 µl of the Pt/C ink was dropcasted onto Au substrate ($A_{geo}$ = 0.75 cm$^2$) and was dried for roughly 30 minutes. SEM images were then captured, and CV experiments were performed, as illustrated in **Figure S3**.

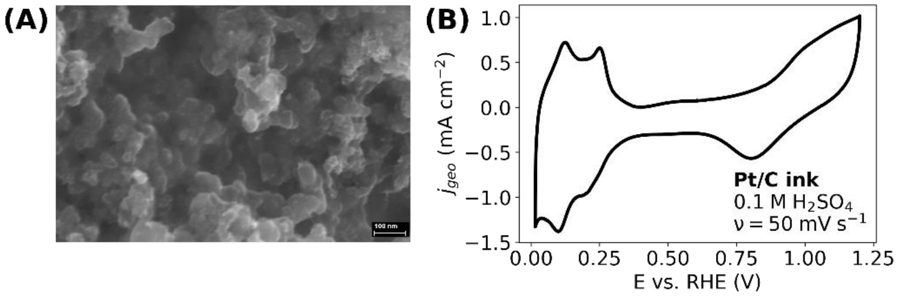


**Figure S3. (A)** SEM image and **(B)** CV of a catalysts ink prepared from commercial Pt/C catalysts. CVs were recorded with the starting potential of +0.0 $V_{RHE}$ in the positive-going potential sweep in $N_2$ purged 0.1 mol dm$^{-3}$ (0.1 M) $H_2SO_4$ using a scan rate of 50 mV s$^{-1}$.

As depicted in **Figure S3.B**, a comparable CV response is observed between the catalysts ink prepared from commercial Pt/C catalysts, and the planar Pt and Pt black electrode (**Figure S2.B and Figure S2.D**).

## 3. Setup of three-electrode flow cell for the *in situ* P *K*-edge XANES investigation

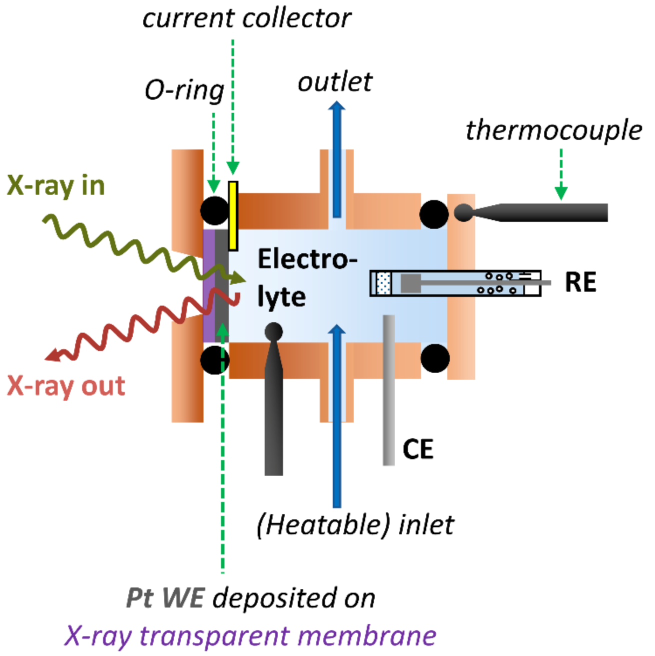


**Figure S4.** Schematic presentation of the three-electrode flow cell used for the *in situ* P *K*-edge XANES experiments at the OÆSE end-station.

The three-electrode flow cell, as well as the tubings for the inlet and outlet connections of the electrolyte, are constructed using PEEK material, which offers excellent chemical resistance against the acids used in this study. The cell comprises a reactor chamber with an approximate volume of 0.750 ml to contain the electrolyte. Inlet and outlet tubings with a diameter of 1.58 mm (equivalent to 1/16") are utilized for fluid flow. Viton® O-rings are used to effectively seal the PEEK cell lid with the X-ray transparent membrane|Pt electrode and the reactor. The cell lid is designed with a funnel-like circular opening, measuring 4.7 mm in diameter, to avoid incoming/outgoing X-rays being blocked. Further information regarding the temperature control and monitoring is provided in Section S8.

## 4. Comparison between XANES spectra of 5 mol dm$^{-3}$ $H_3PO_3$ recorded with rapid beam blocking method and with a radiation-attenuating filter

Two different approaches of minimizing irradiation doses were used for the XANES experiments: (i) rapid blocking of incoming X-rays when no signal is recorded, and (ii) by utilizing an irradiation attenuating filter. The procedure for the first approach is detailed in the main text. For the second approach, a 36 μm thick Kapton® membrane served as a filter to attenuate the incoming X-rays to ≈ 21.2% of the original intensity. The filter was positioned after the last optical elements of the EMIL beamline. To precisely measure the incoming X-ray photon flux ($I_0$), a photodiode (ODD-AXU-010, Optodiode) was placed in the trajectory of the incoming X-rays near the probed Pt|aqueous electrolyte interface. **Figure S5** illustrate a comparison of the P *K*-edge XANES of planar Pt|5 mol dm$^{-3}$ $H_3PO_3$ obtained with these different methods.

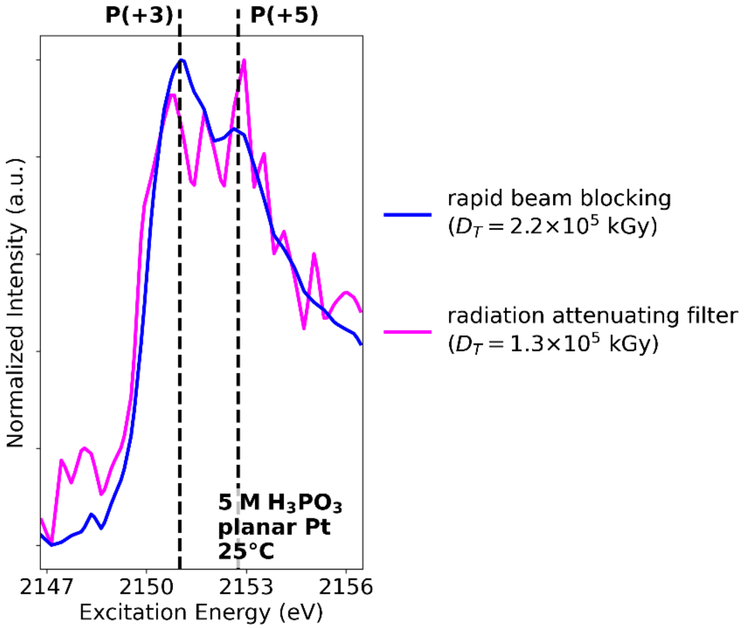


**Figure S5**. Comparison of 5 mol dm$^{-3}$ $H_3PO_3$ XANES spectra recorded with the rapid beam blocking methods and with a radiation-attenuating filter. Irradiation dose was estimated by using Eq. S8, as detailed in section S12. The XANES experiment with rapid beam blocking was conducted with the incoming photon flux of ~ 7.7 × 10$^{11}$ photons s$^{-1}$, while the XANES experiment with radiation attenuating filter was performed with the incoming photon flux of ~1.6 × 10$^{11}$ photons s$^{-1}$. The radiation exposure time to the sample were: 163 seconds and 265 seconds, for the XANES experiment exploiting the rapid beam blocking and the radiation attenuation filter, respectively.

**Figure S5** demonstrates a higher signal-to-noise ratio for the XANES measurement recorded with rapid beam blocking compared to that recorded with an irradiation attenuating filter. Therefore, for the bulk of the study, measurements were conducted using the rapid beam blocking method.

## 5. Fluorescence grid map of 5 mol dm$^{-3}$ $H_3PO_3$ on Pt electrodes and sequential XANES measurement positions

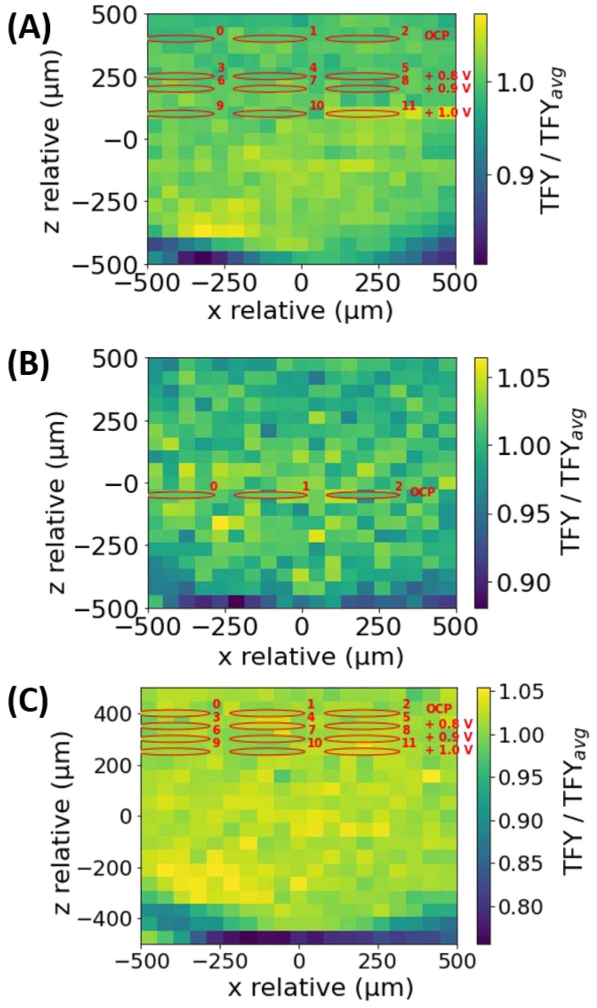


**Figure S6.** Fluorescence grid map of the 5 mol dm$^{-3}$ $H_3PO_3$ on Pt electrodes for *in situ* P *K*-edge XANES investigations under different experimental conditions: **(A)** using a planar Pt electrode at 25 °C, **(B)** employing a planar Pt electrode at 75 °C, and **(C)** utilizing a rough Pt black electrode at 25 °C. Elliptically marked regions illustrate the XANES measurement positions. Three sequential XANES spectra are recorded for each experimental condition, e.g. the potential applied to the working electrode, as indicated by the red labeled text (such as: “OCP”; +0.8 V, etc). The red colored number next to the ellipse represents the measurement number (measurement numbers start from 0). The size of the ellipse corresponds approximately to the size of the beam spot used in this measurement (~237 µm × 37 µm). Each measurement is separated by a distance larger than the beam spot. The fluorescence maps were obtained with an incoming X-ray energy of 2152.5 eV, corresponding to the white line energy position of $H_3PO_4$, ensuring high sensitivity to the generated $H_3PO_4$ resulting from the oxidation of $H_3PO_3$.

## 6. Considerations for the energy resolution of the *in situ* P *K*-edge XANES measurements

In this study, the experimental energy resolutions were approximated by the square root summation of: **(i)** the energy resolution of the beamline, **(ii)** the natural width of the probed transition, and **(iii)** the experimental energy steps, as presented in the SI of Ref. [4], i.e., by the following equation:

$$\sigma_{\text{exp. res.}} = \sqrt{\sigma_{\text{BL. res.}}{}^2 + \sigma_{\text{nat. width}}{}^2 + \sigma_{\text{en. step}}{}^2} \qquad \text{Eq. S4}$$

The beamline energy resolution is ≈ 0.25 eV, for the excitation energy of 2.14 keV. The natural width of the P *K*-edge transition is 0.53 eV [5]. The experimental energy step is 0.25 eV. This translates to the energy resolution of 0.64 eV for the *in situ* P *K*-edge XANES experiments.

## 7. Sequential P *K*-edge XANES of planar Pt|5 mol dm$^{-3}$ $H_3PO_3$ without electrolyte flow at different measurement positions

To investigate the influence of irradiation to the P *K*-edge XANES of 5 mol dm$^{-3}$ $H_3PO_3$, sequential P *K*-edge XANES measurements on planar Pt|5 mol dm$^{-3}$ $H_3PO_3$ were performed using low irradiation dose without electrolyte flow on two different positions, illustrated in **Figure S7**.

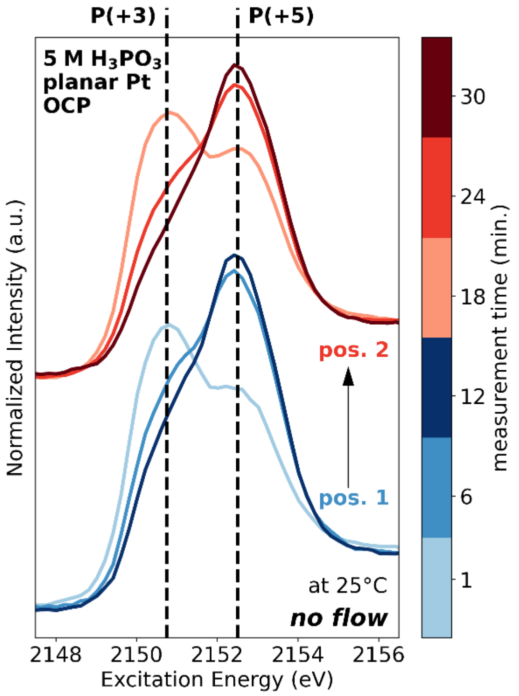


**Figure S7.** Sequential P *K*-edge XANES measurements of planar Pt|5 mol dm$^{-3}$ $H_3PO_3$ recorded as a function of time at two different measurement spots separated by a distance larger than the beam spots. The measurements were conducted without electrolyte flow at room temperature (≈ 25 °C), at the open circuit potential (OCP).

For measurements performed at the same measurement position, there is a progressive increase of spectral weight corresponding to P compounds with the oxidation state of (+5) (e.g., $H_3PO_4$- like compounds), likely indicating the formation of $H_3PO_4$ over time due to the radiation-induced oxidation of aqueous $H_3PO_3$ to $H_3PO_4$. Details on radiation-induced oxidation of aqueous $H_3PO_3$ is discussed in section 3.2. of the main text.

Subsequently, when the measurement is conducted at a different spot (position 1 → position 2), there is a significant decrease in spectral weight corresponding to the P (+5) compound compared to the last XANES recorded at position 1 (see dark blue curve and light red curve at **Figure S7**). Yet, the first XANES spectrum at position 2 (light red curve, measured at t = 18 minutes) seems to display a slightly higher spectral weight corresponding to the P (+5) compound compared to the first XANES spectrum recorded at position 1 (light blue curve, measured at t = 1 minute). This might potentially correspond to the diffusion of $H_3PO_4$ formed from the previous XANES experiment at position 1.

Therefore, to avoid a local increase of irradiation dose and to flush away possible products generated by irradiation (e.g., $H_3PO_4$), for the remainder of this study the sequential XANES was conducted on three different positions at a distance larger than the beam spot with constant electrolyte flow of 0.05 ml min$^{-1}$ throughout the experiments (by the use of syringe pump Legato110, KD Scientific). This ensures that the electrolyte in the ~0.75 ml reactor chamber is renewed after three sequential XANES scans. The sequential XANES measurements recorded with this approach (shown in **Figure 2.B** in the main text, for XANES of planar Pt|5 mol dm$^{-3}$ $H_3PO_3$) exhibit a very small standard deviations over multiple measurements, which suggest that this approach of minimizing the radiation dose is effective in sufficiently suppressing undesirable radiation-induced effects.

## 8. Temperature control and monitoring during *in situ* XANES measurement

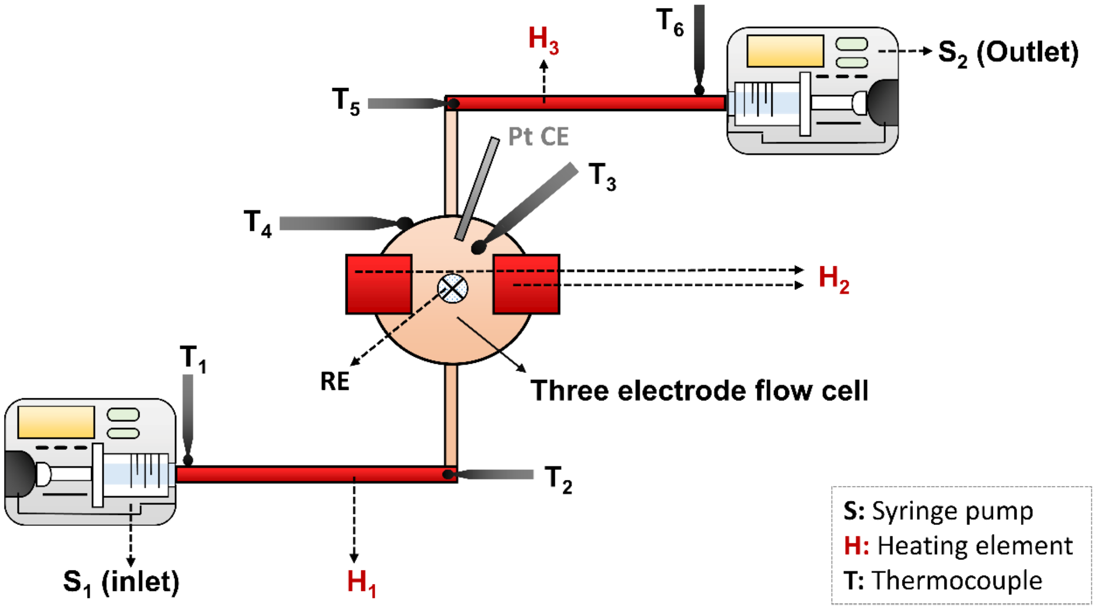


**Figure S8**. Illustration of the heating and temperature monitoring system for the *in situ* P *K*-edge XANES investigation. The components labeled S, T, and H represent the syringe pumps, thermocouples, and heating elements, respectively.

Two syringe pumps (Legato110, KD Scientific) were utilized in a push-pull mode with an electrolyte flow rate of 0.05 ml min$^{-1}$ to prevent exposure of the electrolytes to the outer environment, thereby avoiding potential contamination from air dissolution ($O_2$, $CO_2$, etc.). The chosen flow rate served two purposes: (1) to ensure complete regeneration of the electrolyte volume after three sets of XANES measurements, as a minimum of three XANES scans were taken for each experimental condition, and (2) to prevent excessive pressure on the X-ray transparent membrane, which could lead to membrane breakage during measurements at higher flow rates.

For temperature control, heating wires (Ni-wire, Heraeus Hanau) were applied to raise the temperature of the inlet electrolyte ($H_1$) and outlet electrolyte ($H_3$), and are sealed with thermal insulating tape (K-Flex ST). Additional heating elements were placed on the cell body ($H_2$) to maintain a stable temperature during measurements.

For temperature monitoring, PFA-coated thermocouples were employed (CASS-IM15G-300-PFA, OmegaEngineering), to prevent corrosion of thermocouples due to harsh experimental conditions. Temperature sensors $T_1$ and $T_6$ monitored the heating cable's temperatures. $T_2$ and $T_5$ were designated for monitoring the inlet and outlet electrolyte temperatures, respectively. $T_3$ provided temperature readings of the electrolyte within the reactor chamber (where the electrode is in contact with the electrolyte). Lastly, $T_4$ was used to monitor the temperature of the three-electrode flow cell body.

**9. Considerations for effective detection depth in the electrolyte for the *in situ* P *K*-edge XANES experiments**

To estimate the effective detection depth in the electrolyte for the *in situ* P *K*-edge XANES experiments, the electrode|electrolyte interface irradiated by the X-rays was modelled as depicted in **Figure S9**.

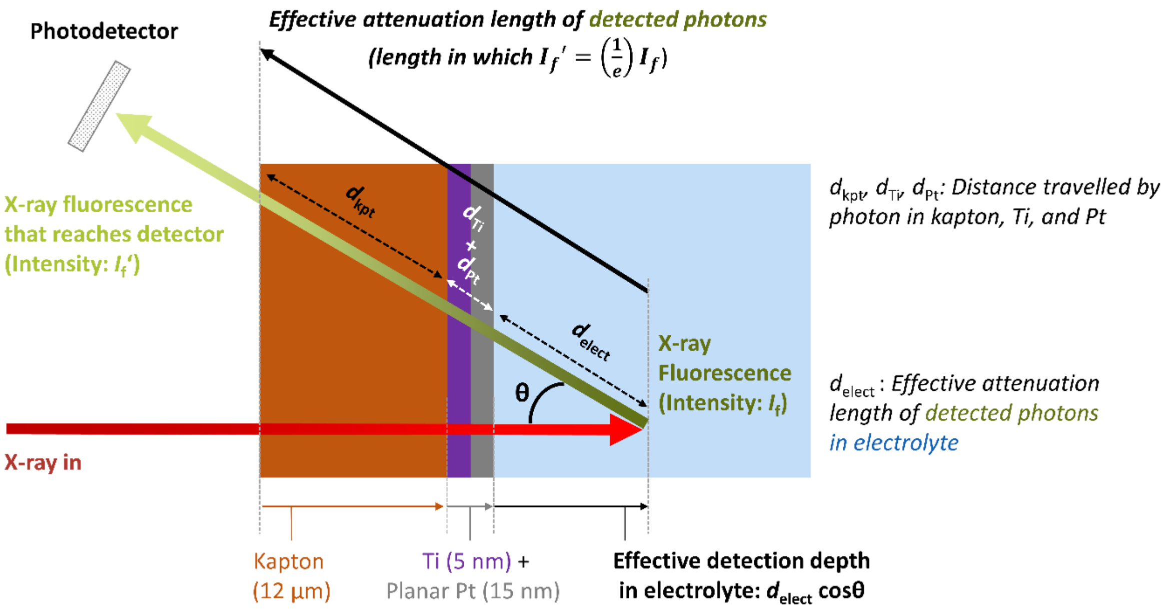


**Figure S9.** Illustration of the experimental setup depicting the electrode|electrolyte interface probed by the X-rays. The incoming X-rays probed the sample at angle close to 90°, while the X-ray fluorescence are detected at an angle of θ = 45° relative to the sample surface. Note that the length shown in the illustration is not up to scale. Further details regarding the estimation of the 'effective attenuation length of probing photon's and the ‘effective detection depth in electrolyte’ are provided in the following text.

In our setup, the incoming X-ray probes the electrode|electrolyte interface at an angle nearly perpendicular to the sample surface, while the fluorescence photons are recorded at an angle of θ = 45° relative to the sample surface. Thus, the incoming X-rays probe farther into the sample than the depth from which X-ray fluorescence photons can be detected. This is because the detected X-ray fluorescence photons needs to travel a longer path in the material (as illustrated in **Figure S9**). As a result, the detected X-ray fluorescence will likely experience higher attenuation compared to the incoming X-ray. For this reason, given that the XANES measurement is conducted in FY-mode (i.e., by recording the X-ray fluorescence from the sample), considerations for effective detection depth in this investigation is based on the effective attenuation length of detected photons.

In this study, the ‘effective attenuation length of detected photons’ is defined as the length at which the intensity of the X-ray fluorescence has been attenuated to (1/e = 0.367) of their initial intensity ($I_0$) as it probes through the electrolyte, Pt electrode, Ti adhesion layer, and Kapton window, as shown in **Figure S9**. The attenuation of photon intensity through the different layers can be described by Lambert-Beer’s Law $\left(I(x) = I_0 e^{-\sum(\mu_i(\rho,E)\Delta x_i)} = I_0 \sum T_i(\rho, E, x)\right)$ (see Ref. [6]). By using Lambert-Beer's Law,

the thickness of the electrolyte that the fluorescence X-rays need to pass through until the intensity is attenuated to (1/e) of its initial value can be determined by Eq. S5.

$$I_{at\,att.\,len.} = I_0\frac{1}{e} = I_0 e^{-(\mu_{\mathrm{kpt}} d_{\mathrm{kpt}} + \mu_{\mathrm{Ti}} d_{\mathrm{Ti}} + \mu_{\mathrm{Pt}} d_{\mathrm{Pt}} + \mu_{\mathrm{elect.}} d_{\mathrm{elec.}})} = I_0\, T_{\mathrm{kpt}}\, T_{\mathrm{Ti}}\, T_{\mathrm{Pt}}\, T_{\mathrm{elec.}} \qquad \text{Eq. S5}$$

Here, μ, *d*, and *T* represent the absorption coefficient, distance travelled by the photons, and transmittance of each material probed by the X-rays, respectively. *Kpt, Ti, Pt,* and *elec.* stands for the Kapton membrane, Ti adhesion layer, Pt electrode, and electrolyte, respectively. $d_{\mathrm{Kpt}}$, $d_{\mathrm{Ti}}$, and $d_{\mathrm{Pt}}$ represents the distance that the fluorescence photons need to travel through these materials before the photons reach the detectors. Approximately, this distance is given by $d_{material} = \frac{thickness\ of\ material}{\cos\theta}$, which for θ = 45° corresponds to 16.9 µm, 7.01 nm, and 21.2 nm for $d_{\mathrm{Kpt}}$, $d_{\mathrm{Ti}}$, and $d_{\mathrm{Pt}}$, respectively. The transmittances for 16.9 µm Kapton, 7.01 nm Ti, and 21.2 nm Pt are 0.46, 0.97, and 0.88 at the X-ray energy of 2139 eV (the energy of P $Kß_1$ X-ray fluorescence, as detailed in Ref. [7]), respectively, as determined from LBL (Lawrence Berkeley Laboratory) X-ray filter transmission database [8], which is based on Ref [9]. Here considerations were conducted with P $Kß_1$ fluorescence, since this X-ray fluorescence line has higher energy compared to P $K\alpha_1$ line or P $K\alpha_2$ (~2014 eV or 2013 eV [7]), therefore the P $Kß_1$ fluorescence determined the 'maximum' detection limit among all detected the X-ray fluorescence photons. By incorporating these transmittance values into Eq. S5, it can be shown that the electrolyte needs to possess a transmittance of 0.40 to attenuate the X-ray fluorescence intensity to (1/e) of its initial intensity. Given that $H_3PO_3$ in 5 mol dm$^{-3}$ $H_3PO_3$ electrolyte possesses the density of: $\rho_{5MH3PO3}$= c × $M_{H3PO3}$ = 5 mol dm$^{-3}$ × 81.99 g mol$^{-1}$= 409.95 g dm$^{-3}$, using the same X-ray transmission database, this transmittance corresponds to approximately 5.5 µm of the electrolyte layer.

Hereafter, the electrolyte thickness required to attenuate the X-ray fluorescence to this value will be referred to as the 'effective attenuation length of *detected photons*'. Beyond the attenuation length of detected photons, the generation of X-ray fluorescence still occurs as the incoming X-ray still probes deeper into the electrolyte. However, the intensity of X-ray fluorescence that reaches the detector beyond this point is very small compared to the total intensity of X-ray fluorescence that is recorded, and therefore it is not considered. Hence, in this study, the length at which the probing photons (i.e., the incoming X-rays) need to travel in the electrolyte, until they reach the point corresponding to the attenuation length of detected photons, is considered as the '*effective detection depth in the electrolyte*'. The effective detection depth in the electrolyte can be estimated by the multiplication of the 'effective attenuation length of detected photons' with the cosines of the angle between the detector and the sample (here θ = 45°). This corresponds to an effective detection depth of ~3.8 µm for the planar Pt electrode (deposited on Kapton|Ti layer). Please note however, that this value is overestimated, since the estimation was performed by approximating the transmittance of electrolyte layer with the transmittance of $H_3PO_3$ in 5 mol dm$^{-3}$ $H_3PO_3$ electrolyte only. In reality, the 5 mol dm$^{-3}$ $H_3PO_3$ electrolyte also consist of $H_2O$ (71 wt.% $H_2O$), and as a result, more X-rays are absorbed by the $H_2O$, leading to a smaller

effective attenuation length. For instance, when the same considerations are made using the transmittance of $H_2O$ ($\rho_{H2O} \approx 1000$ g cm$^{-3}$), this corresponds to an effective probing depth in the electrolyte of ~1.41 µm. In this study, considerations are made with the transmittance of $H_3PO_3$ in the 5 mol dm$^{-3}$ aqueous electrolyte, to provide a value of 'maximum' detection depth in the electrolyte.

Using similar procedures, the effective detection depth in the electrolyte can be approximated for XANES measurements using ~10 nm Pt black electrode (deposited on Kapton|Ti|planar Pt). This estimation leads to the effective detection depth in the electrolyte of approximately 0.71 µm for Pt black electrode. It is important to note that this approximation was made with the assumption of a flat layer, and therefore, it might not be highly accurate for the Pt black electrode with high surface roughness. However, despite its limitations, this estimation provides an approximate value for the different effective detection depths between the electrolyte, as the observability of the effects from Pt surface significantly depends on the effective detection depth in the electrolyte.

For 12 µm thick 'Pt free' Kapton substrate (without Ti|planar Pt layer), more careful considerations are needed. Using a similar procedure for estimation leads to an effective detection depth of ~15 µm. However, in the case of the 12 µm thick Kapton and the experimental geometry described earlier, the 'effective attenuation length of *probing photons*' is approximately 8.8 µm, which is lower than the 'effective attenuation length of detected photons' (Considerations for the 'effective attenuation length of *probing photons*' are detailed in the section S12). This is not the case for the planar Pt electrode and Pt black electrode, where the effective length of probing photons is much higher than the detected photons (approximately 7 µm and 6 µm for planar Pt and Pt black, respectively, as detailed in section S12). Therefore, for the 'Pt free' Kapton case, the effective detection depth in the electrolyte is determined by the 'effective attenuation length of probing photons' (~8.8 µm), as the intensity of X-ray fluorescence generated beyond this length is very small compared to the total intensity of recorded photons.

In this estimation of effective detection depth, the bending angle of Kapton|Ti|planar Pt membrane, due to the pressure difference between the reactor chamber (at approximately $10^3$ mbar) and the UHV condition in the beamline (at the pressure of ~9 × $10^{-8}$ mbar or less), is not considered, as the bending angle under these experimental conditions is very small (< 1°). The bending angle can be estimated using the following approximation.

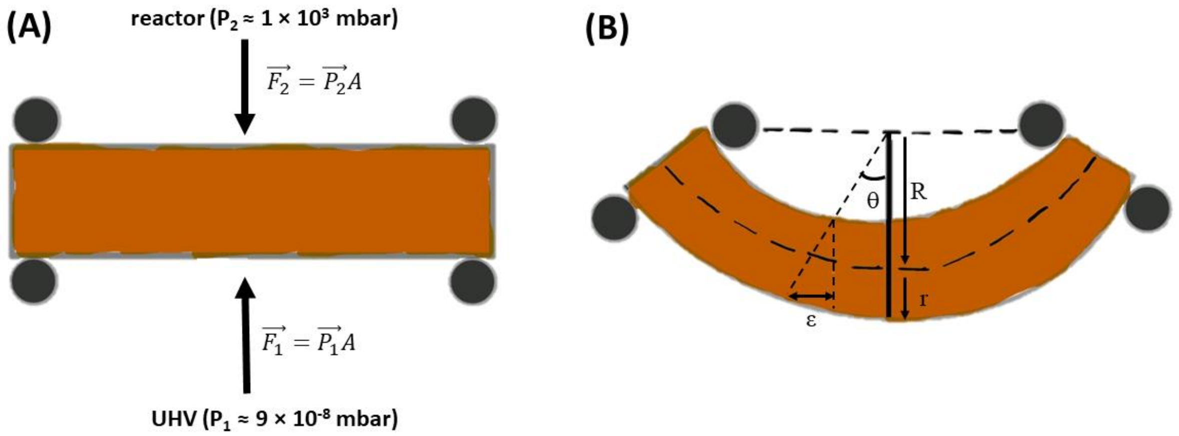


**Figure S10.** Schematic illustration of **(A)** the pressure difference between the reactor chamber and UHV condition in the beamline and **(B)** the bending of Kapton/electrodes due to pressure difference between the pressure difference. Here ε represents the elongation due to the pressure difference and θ corresponds to the bending angle. Note that the bending illustration is exaggerated for visualization.

At **Figure S10**, ε is the elongation of the sample due to the pressure difference, r is estimated as half of the thickness of the material, and σ is the shear stress experienced by the sample. In this estimation, the bending angle can be approximated by $\theta = \frac{1}{R} = \frac{\varepsilon}{r}$. Since shear stress and the elongation of the materials are correlated by the respective material's Young's modulus (i.e., by $Y = \frac{shear\ stress}{shear\ strain} = \frac{\sigma}{\varepsilon/r}$ ), the bending angle of material under applied stress can be estimated from the stress and the material's Young's modulus by the following equation: $\theta = \frac{\sigma}{Y}$.

The shear stress of the sample can be expressed by the resultant of pressure experienced by the sample: $\sum \sigma \approx \left(\frac{F_1}{A}\right) - \left(\frac{F_2}{A}\right) = P_1 - P_2$. In this experiment, $\sum \sigma \approx 10^3$ mbar. Since Kapton layer is thicker (12 µm) than the Pt layer (15 nm) and the Ti layer (5 nm), the bending is mostly determined by the Kapton. Given that Kapton® possess a Young Modulus of 2.5 GPa [10], the bending angle is ≈ $4 \times 10^{-5}$ °. Given this small bending angle, the effect of the Kapton|Ti|planar Pt membrane bending on the angle of the detector for the approximation of effective detection depth is not considered. Additionally, the effect of electrolyte flow to the bending of the membrane is also not considered, since low flow rate is used (0.05 ml $min^{-1}$) and the electrolyte flow vertically from the bottom of the reactor to the top of the reactor, i.e., parallel to the Kapton|Ti|Pt electrode membrane.

10. **Theoretical estimation of the probed electrode-surface-to-electrolyte-volume ratio for the P *K*-edge XANES experiments**

To evaluate the relative contribution of the XANES signal arising from the probed Pt electrode surface compared to the overall XANES signal originating from the entire electrolyte volume, a theoretical estimation is conducted. This estimation was made by comparing the number of probed $H_3PO_3$ molecules on the electrode surface to the total number of $H_3PO_3$ molecules in the entire electrolyte volume probed by XANES.

In this estimation, it is assumed that the probed electrode surface is composed of metallic Pt, for metallic Pt possesses a high catalytic activity towards the oxidation of aqueous $H_3PO_3$, as observed in the previous study [11]. Consideration is made for a static system, in which molecules do not move in the electrolytes. Furthermore, this estimation was made with the assumption that the $H_3PO_3$ in contact with the electrode surface will undergo an oxidation process, thereby contributing to a change in the XANES spectra. Thus, this estimation provides a lower limit of observed $H_3PO_4$ due to the catalyzed oxidation of $H_3PO_3$ by Pt.

The number of Pt atoms in the monolayer surface area probed by XANES can be estimated using Eq. S6.

$$number\ of\ Pt\ atoms\ covering\ monolayer\ surface = \left(\frac{\theta_{Pt}\, N_A}{F}\right) \times \left(A_{probed}\ rf_{WE}\right) \qquad \text{Eq. S6}$$

$\theta_{Pt}$ corresponds to the specific surface charge of a Pt monolayer (210 µC $cm^{-2}$, according to ref. [1,2]), F represents the Faraday constant (F = $9.648 \times 10^4$ C $mol^{-1}$), and $N_A$ is the Avogadro's constant ($N_A$ = $6.022 \times 10^{23}$ $mol^{-1}$). $rf_{WE}$ corresponds to the roughness factor of the working electrode. $rf_{WE}$ ~1.41 for planar Pt electrode and ~7.1 Pt black electrode, respectively, as detailed in Section S2. $A_{probed}$ represents the surface area of the probed electrode, corresponding to the area of the beam spot: $A_{probed} = \pi \times 237$ µm × 37 µm= $2.75 \times 10^4$ $µm^2$.

The total number of $H_3PO_3$ molecules in the electrolyte volume probed by XANES can be estimated using Eq. S7.

$$Number\ of\ probed\ H_3PO_3\ molecules = \left(V_{probed} \times c\right) \mathrm{N_A} \qquad \text{Eq. S7}$$

$c$ is the concentration of the probed $H_3PO_3$ electrolyte ($c_{H3PO3}$= 5 mol $dm^{-3}$) and $V_{probed}$ corresponds to the electrolyte volume probed by the solution (in $dm^{-3}$).

The ratio of the XANES signal arising from $H_3PO_3$ on the electrode surface to the total signal originating from $H_3PO_3$ in the entire probed electrolyte can be estimated by dividing the number of Pt atoms covering the monolayer surface (determined using Eq. S6, assuming that one Pt atom is in contact with one $H_3PO_3$ molecule) to the number of probed $H_3PO_3$ molecules (approximated using Eq. S7).

For 5 mol $dm^{-3}$ $H_3PO_3$ electrolyte, the probed-surface-to-volume ratio is approximately 4.4% for Pt black and about 0.2% for planar Pt. In the assumption of a static condition (i.e., assuming no diffusion)

and that one Pt atom is oxidizing one $H_3PO_3$ molecule in contact with it, at the OCP, it is within reason to assume that the number of $H_3PO_4$ formed by Pt-catalyzed oxidation of aqueous $H_3PO_3$ is similar to the probed-electrode-surface-to-electrolyte-volume-ratio. This supports the significant increase of spectral weight corresponding to P compounds with oxidation state of (+5), such as $H_3PO_4$-like compounds, observed in the XANES of 5 mol dm$^{-3}$ $H_3PO_3$ on Pt black (see **Figure 1.C** of the main text).

Additionally, it is important to note that while a significant portion of the XANES signal arises from the X-ray fluorescence generated from the bulk electrolyte volume irradiated with the X-rays, the recorded intensity of X-ray fluorescence generated from the electrolyte near the surface is expected to be higher than the recorded intensity of X-ray fluorescence from the bulk solution, even though the intensity from this near-surface layer only makes up a small portion of the total recorded intensity.

This phenomenon is illustrated in **Figure S11**, modelling the X-ray absorption and fluorescence by layers of electrolyte as the incoming X-rays penetrate deeper into the electrolyte layer. Initially, incoming X-rays with the initial intensity of $I_0$ are attenuated by the Kapton, Ti, and Pt layer by a factor of: $I_0 e^{-(\mu_{\mathrm{kpt}} d_{\mathrm{kpt}} + \mu_{\mathrm{Ti}} d_{\mathrm{Ti}} + \mu_{\mathrm{Pt}} d_{\mathrm{Pt}})} = I_0 T_{\mathrm{kpt}} T_{\mathrm{Ti}} T_{\mathrm{Pt}}$, as they reach the electrolyte. Here $\mu_i$, $d_i$, and $T_i$ represent the absorption coefficient, thickness, and transmittance of the attenuating layer I, respectively. Upon reaching the first electrolyte layer next to the electrode surface, absorption occurs, leading to the fluorescence of the electrolyte layer. The intensity of X-ray fluorescence ($I_f$) is proportional to the incoming X-ray intensity ($I_i$) reaching the sample, following the relationship $\mu \propto \frac{I_f}{I_i}$ (see Ref.[6]). Thus, the fluorescence intensity is: $I_f = C_f I_i = C_f I_0 (T_{\mathrm{kpt}} T_{\mathrm{Ti}} T_{\mathrm{Pt}})$, where $C_f$ represents a proportionality constant correlating the generated fluorescence to the incoming intensity (note that $C_f < 1$). The fluorescence X-rays then travel back through the Kapton, Ti, and Pt layers where they are attenuated, and upon reaching the detector, they possess the intensity of: $I_f' = I_f e^{-\left(\mu_{\mathrm{kpt}} \frac{d_{\mathrm{kpt}}}{\cos\theta} + \mu_{\mathrm{Ti}} \frac{d_{\mathrm{Ti}}}{\cos\theta} + \mu_{\mathrm{Pt}} \frac{d_{\mathrm{Pt}}}{\cos\theta}\right)} = I_f\, T_{\mathrm{kpt}}'\, T_{\mathrm{Ti}}'\, T_{\mathrm{Pt}}{}' = I_0 C_f (T_{\mathrm{kpt}}\, T_{\mathrm{Ti}}\, T_{\mathrm{Pt}})(\, T_{\mathrm{kpt}}'\, T_{\mathrm{Ti}}'\, T_{\mathrm{Pt}}')$. Where θ is the angle between the sample and the photon detector and $T_i$' corresponds to the transmission of material *i* for the thickness of ($d_i$/cos θ).

Now, consider that most of the intensity of the incoming X-rays is not absorbed by the first layer of the electrolyte but rather travels through a thickness of the electrolyte layer ($\Delta x$) before some portion of the incoming X-rays is absorbed by the electrolyte and induces fluorescence. The incoming intensity at this point is $I = I_0 (T_{kpt} T_{Ti} T_{Pt}) (T_{\Delta x})$, where $T_{\Delta x}$ is the transmittance of the electrolyte layer with the thickness of $\Delta x$. This process leads to fluorescence with the intensity of $I_f = I_0 C_f (T_{\mathrm{kpt}} T_{\mathrm{Ti}} T_{\mathrm{Pt}})(T_{\Delta \mathrm{x}})$. Then the fluorescence will pass through the electrolyte layer of thickness ($\Delta x$/cos θ), layers of Pt, Ti, and Kapton before reaching the detector, where the intensity becomes $I_f' = I_0 C_f (T_{\mathrm{kpt}} T_{\mathrm{Ti}} T_{\mathrm{Pt}}) (T_{\mathrm{kpt}}' T_{\mathrm{Ti}}' T_{\mathrm{Pt}}') (T_{\Delta \mathrm{x}} T_{\Delta \mathrm{x}}')$. Here, $T_{\Delta \mathrm{x}}$' corresponds to the transmission of the electrolyte layer for the thickness of ($\Delta x$/cos θ).

This process occurs many times (*n* times) for each part of the electrolyte layer (with a thickness of $\Delta x$) until the X-rays reach the effective detection depth in the electrolyte. After the effective detection depth,

this process still occurs, but the intensity of X-ray fluorescence that reaches the detectors is very small compared to the intensity of X-ray fluorescence that is generated within the effective detection depth and therefore is not considered. The incoming X-rays that reach the last layer of electrolyte (at the effective detection depth in the electrolyte) will have the intensity of $I_n = I_0\,(T_{kpt}\,T_{Ti}\,T_{Pt})\,(T_{\Delta x})^{n-1}$. This induces fluorescence with an intensity of $I_f = I_0\,C_f\,(T_{kpt}\,T_{Ti}\,T_{Pt})\,(T_{\Delta x})^{n-1}$, which needs to travel through $n$ layers of electrolyte, Pt, Ti, and Kapton before reaching the detector. At this stage, the fluorescence X-rays have an intensity of $I_f' = I_0\,C_f\,(T_{kpt}\,T_{Ti}\,T_{Pt})\,(T_{kpt}'\,T_{Ti}'\,T_{Pt}')\,(T_{\Delta x}\,T_{\Delta x}')^{n-1}$. This process is illustrated in **Figure S11**.

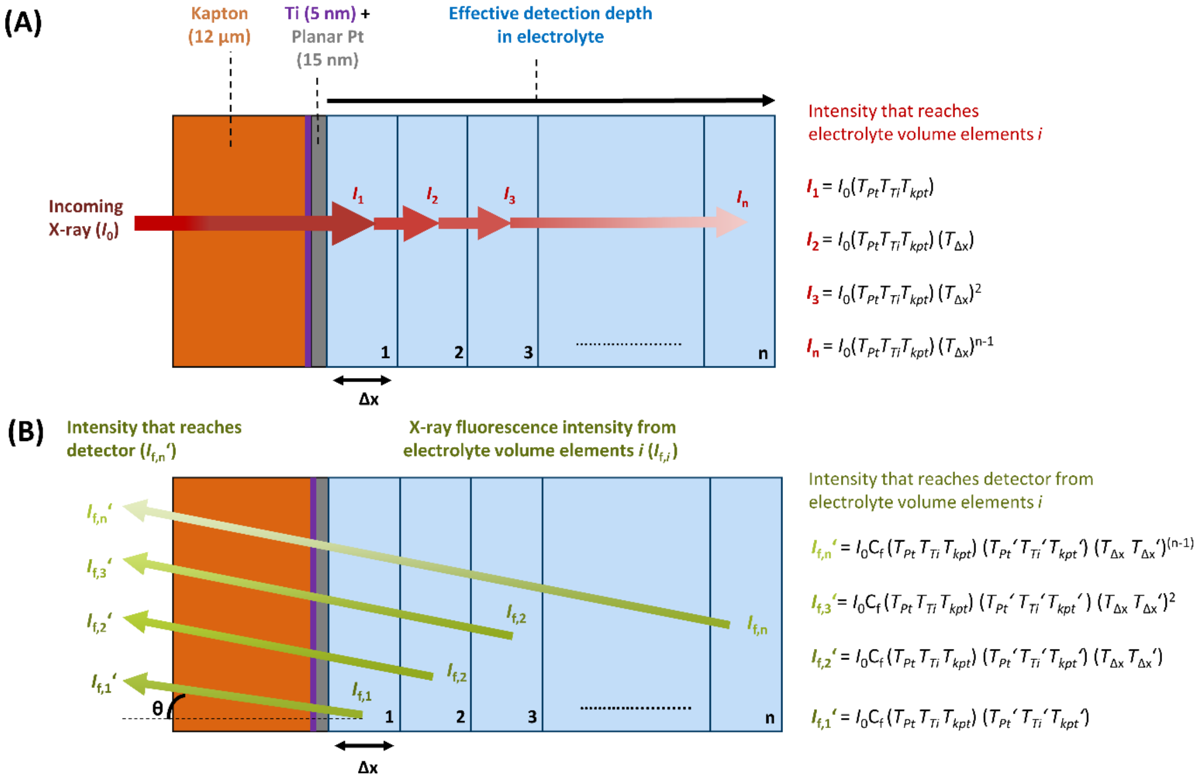


**Figure S11.** Model illustrating the X-ray absorption and X-ray fluorescence by a layer of electrolyte as the incoming X-rays penetrate deeper into the electrolyte layer. In this model, each individual layer of electrolyte possesses a thickness of $\Delta x$. Panel **(A)** illustrates the absorption of the incoming X-ray by each layer of electrolytes, until the $n^{th}$ layer of electrolyte at a distance corresponding to the effective detection depth in the electrolyte. Panel **(B)** illustrates the generation of X-ray fluorescence by each electrolyte layer, following the X-ray absorption that occurred in panel (A). $I_{f,n}$ represents the intensity of X-ray fluorescence generated from volume element $n$, which is dependent on the absorbed X-ray intensity. $\theta$ corresponds to the angle between the detector and the sample. As the fluorescence travels back through the electrolyte and passes through layers of Pt, Ti, and Kapton before reaching the detector, its intensity becomes $I_{f,n'}$, representing the fluorescence intensity detected by the XANES setup in fluorescence mode.

Using this model, it is possible to estimate the ratio of X-ray fluorescence generated from different layers of the electrolyte to the total fluorescence intensity. For example, in the P *K*-edge XANES of 5 mol dm$^{-3}$ $H_3PO_3$ with the detector angle of θ = 45°, by considering an electrolyte layer thickness of Δ*x* = 50 nm, where each electrolyte element has a transmittance of $T_{\Delta x}$ = 99.7% (the transmittance of $H_3PO_3$ given the density of [$\rho_{5MH3PO3}$= c × $M_{H3PO3}$ = 5 mol dm$^{-3}$ × 81.99 g mol$^{-1}$= 409.95 g dm$^{-3}$] at 2156.5 eV) and $T_{\Delta x}$'= 99.8% (the transmittance of the $H_3PO_3$ in 5 mol dm$^{-3}$ solution at 2139 eV for the thickness of Δ*x*/cos θ according to the LBL X-ray transmission database[8]), the ratio of fluorescence intensity between originating from each electrolyte layer to the total intensity of fluorescence from each all electrolyte layers within the effective detection depth in electrolyte can be made. **Figure S12** illustrates such ratio for all the different electrodes in this study. This example is made by considering each 50 nm thick electrolyte layer as a single element, thus each data point in the figure only represents the ratio from a *convolution* of signal in the whole 50 nm thick layer vs the total considered fluorescence signal (from the electrode surface to the estimated effective detection depth in the electrolyte). Despite this fact, the example was made with this electrolyte layer thickness to ensure reasonable approximation accuracy, since with a thinner electrolyte layer, the transmittance of the layer is very close to 100% (as per Lambert-Beer's Law), and thus estimation is less accurate. For instance, if estimations were made for the 5 mol dm$^{-3}$ $H_3PO_3$ electrolyte layer thickness of 10 nm, this result in (i) the transmittance of $T_{\Delta x}$ = 99.9% for the incoming photons at 2156.5 eV, and (ii) the transmittance of $T_{\Delta x}$'= 99.9% for the X-ray fluorescence at 2139 eV (given the thickness of 10 nm /cos (45°) ≈ 14 nm, as according to the same X-ray transmission database [8]).

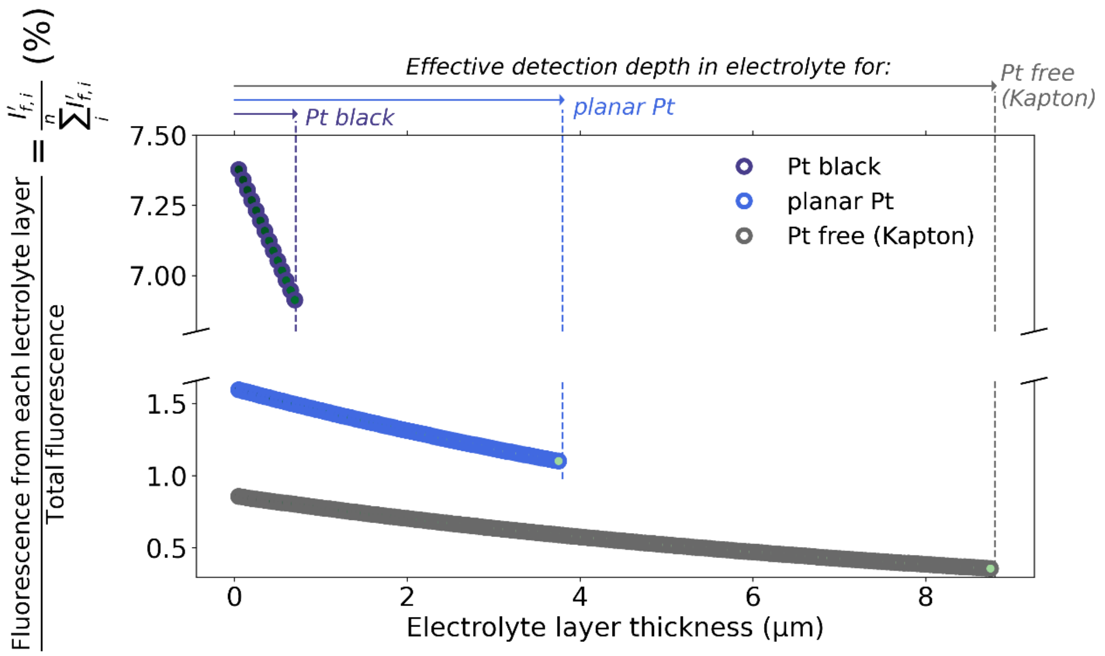


**Figure S12**. Theoretically estimated ratio of recorded fluorescence intensity from each electrolyte layer, *i* ($I_{f,i'}$), compared to the total fluorescence originating from the entire electrolyte layer within the effective detection depth in the electrolyte ($\sum_i^n I'_{f,i}$). The estimations were performed using the model presented

in **Figure S11**, with an electrolyte-volume-elements with a thickness of 0.05 µm (each element possesses a transmittance of $T_{\Delta x}$ = 99.7% and $T_{\Delta x}$'= 99.8% for X-ray energy of 2156.5 eV and 2139 eV, respectively). The x-axis represents the position along the electrolyte layer, where "0" corresponds to the region in direct contact with the electrode (i.e., closer to the incoming X-rays from the beamline), while the maximum distance represents the effective detection depth in electrolyte (approximately 0.71 µm, 3.88 µm, and 8.8 µm for Pt black, planar Pt, and Pt free Kapton, respectively, as detailed in section S8).

As illustrated from **Figure S12**, the intensity of X-ray fluorescence originating from the first 50 nm layer of the electrolyte, contributes approximately 7.4 %, 1.6 %, and 0.9 % of the total recorded intensity for Pt black, planar Pt, and Kapton, respectively. Notably this Figure shows that, Pt black exhibits a significantly higher contribution of X-ray fluorescence from the electrolyte layer near the surface compared to the other electrodes. Furthermore, this graph emphasizes that although the intensity contribution from the layer closest to the electrode is relatively higher, for the XANES recorded on planar Pt and Pt free Kapton, it still constitutes only a small portion of the overall recorded intensity. For this approximation, however, it is noteworthy that the estimation made on the rough Pt black might be less accurate than for the other electrodes. Such is the case, since the approximation of the X-ray fluorescence ratio is made until the effective detection depth of the electrolyte. As discussed in section S9, the consideration for effective detection depth in the electrolyte is made with the assumption of a flat layer. As a result, the estimation of effective detection depth in the electrolyte is less accurate for the rough Pt black than for the other electrodes. Consequently, the approximation or X-ray fluorescence ratio is also less accurate for the Pt black electrode.

Nevertheless, with this approximation, it can be shown that the intensity of X-ray fluorescence originating from the electrolyte layer close to the Pt black electrode is considerably higher for Pt black than planar Pt. Planar Pt also displays a slightly higher ratio compared to 'Pt free' Kapton. This difference in ratio aligns well with the XANES measurement results presented in **Figure 1.C** in the main text, which show an increase in white line intensity ratio related to the features of the P (+5) white line and to the P (+3) white line in the order of 'Pt free' Kapton, planar Pt, and (strong increase) with Pt black.

## 11. Detection of $H_2$ upon the oxidation of aqueous $H_3PO_3$ to $H_3PO_4$

To confirm that upon the oxidation of aqueous $H_3PO_3$ there is a formation of $H_2$ alongside $H_3PO_4$, complementary gas chromatography measurements were conducted on aqueous $H_3PO_3$ solution before and upon dispersion of Pt/C catalysts into the solution.

In this experiment 125 ml of a 0.03 mol $dm^{-3}$ aqueous $H_3PO_3$ solution was placed in a magnetically stirred three-necked round-bottom flask and maintained at room temperature (~25°C). The solution is continuously purged with Ar (flow rate: 25 ml $min^{-1}$), which also serves as a GC carrier and reference gas. The outlet gas from the flask passed through a molecular sieve (3 Å, Alfa Aesar) drier and was sampled to a GC (Focus GC, Thermo Fisher) equipped with a HP-PLOT Molsieve (19095P-MS6) column connected to a Thermal Conductivity Detector (TCD), for characterization of the outlet gas' composition.

Prior to the addition of Pt/C, no measurable amount of $H_2$ gas was generated in the reaction mixture; only the presence of $N_2$ and $O_2$, originating from contamination from air, was visible on the chromatogram (see **Figure S13**). Subsequently, upon the addition of 48 mg of Pt/C catalyst (Hispec4000, Johnson-Matthey, UK) into the solution, a new peak appeared on the chromatogram at the retention time of ~1.65 min., corresponding to that of $H_2$ during calibration (as illustrated in **Figure S13**). This confirms the formation of $H_2$ due to oxidation of aqueous $H_3PO_3$ to $H_3PO_4$, as discussed in the main text. The production of $H_2$ in the system was followed for around 3 hours (180 min.) during which the $H_3PO_3$ concentration decreased by about 17%. As illustrated in **Figure S14**, the rate of $H_2$ production during the experiment decreased slightly from about 200 to 160 μmol $min^{-1}$ $g_{Pt}^{-1}$ and seems to be limited by the amount of the catalyst (Pt).

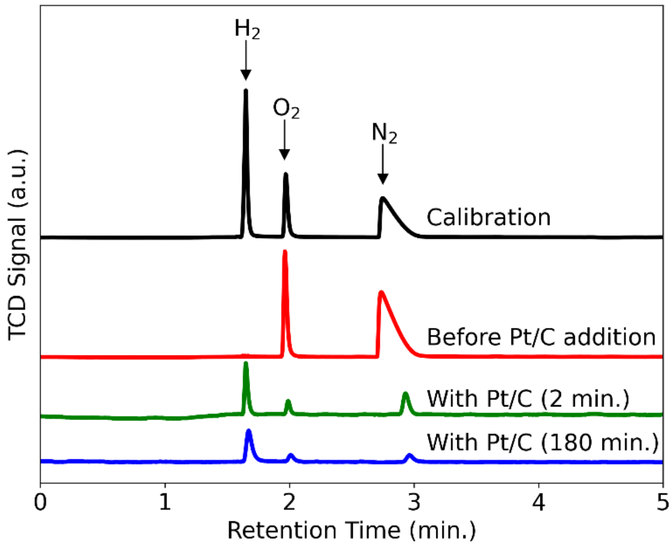


**Figure S13**. Gas chromatograms of: (black) mixture of $H_2$, $O_2$, and $N_2$ in Ar used during calibration; Ar with gas phase above the reaction mixture, (red) before the addition of Pt/C, and (green, blue) at different reaction times following the addition of Pt/C catalysts.

Additionally, it is important to point out that rather long reaction time is observed in this experiment, compared to reaction time of 15 $cm^3$ of 10 mmol $dm^{-3}$ $H_3PO_3$ upon dispersion of 50 mg Pt/C during the ion exchange chromatography (IEC) experiments at 25 °C (illustrated in **Figure 4.D** of the main text). This difference occurs since in the GC experiment, a significantly higher amount of $H_3PO_3$ $\left(\frac{\text{amount of } H_3PO_3 \text{ (GC)}}{\text{amount of } H_3PO_3 \text{ (IEC)}} = \frac{\text{Vol (GC)} \times \text{c(GC)}}{\text{Vol (IEC)} \times \text{c(IEC)}} = \frac{125 \text{ ml} \times 30 \text{ mM}}{10 \text{ ml} \times 10 \text{ mM}} \approx 37 \text{ times}\right)$ and solution volume (around 12 times) was used, while amount of Pt/C catalyst was almost similar in both experiments.

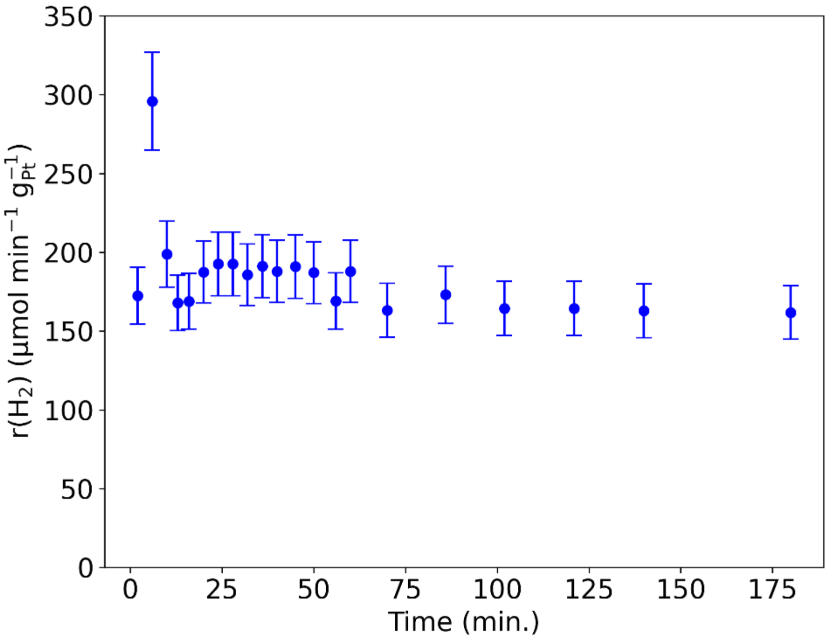


**Figure S14.** Rate of $H_2$ production during first 3 hours following the dispersion of Pt/C catalysts in 125 ml of 0.03 mol $dm^{-3}$ aqueous $H_3PO_3$, due to the Pt-catalyzed reaction between the $H_3PO_3$ and $H_2O$.

## 12. Estimation of irradiation dose absorbed by the electrolyte during the XANES experiments

The total radiation dose absorbed by the sample during the XANES experiment was estimated by Eq. S8

$$D_T = D_r \Delta t = \frac{E N_0}{m} \Delta t \qquad \text{Eq. S8}$$

Here, $D_T$ represents the total radiation dose to the sample (in Gy), $D_r$ corresponds to the radiation rate (in Gy $s^{-1}$), as given by Ref. [12]. $\Delta t$ is the exposure time (in s), $E$ represents the incoming X-ray energy (in J). $N_0$ is the number of photons per unit time at the solution interface (in $s^{-1}$), and $m$ corresponds to the mass of the electrolyte solution absorbing the synchrotron radiation (in kg). $N_0$ was determined just before XANES measurement, by using a photodiode (ODD-AXU-010, Optodiode) placed in the incoming X-rays trajectory, in the vicinity of probed Pt|aqueous electrolyte interface.

To estimate the mass of the electrolyte absorbing the synchrotron irradiation, the probed electrode|electrolyte interface irradiated by the X-rays was modelled. The model is depicted in **Figure S15**.

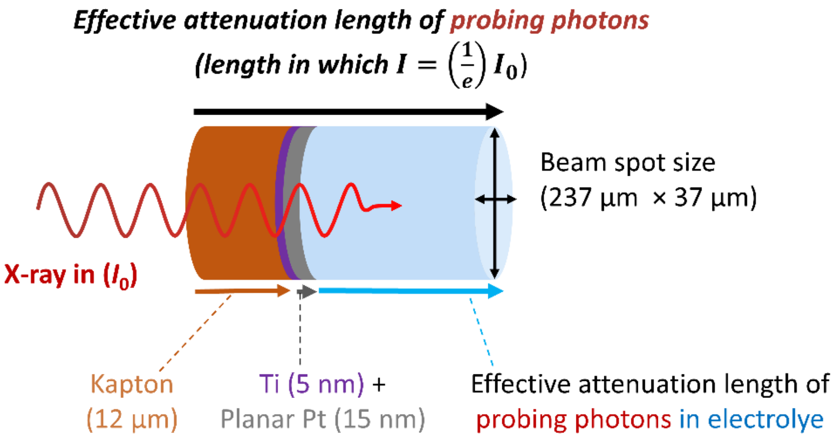


**Figure S15.** Illustration of the model depicting the electrode|electrolyte interface probed by the X-rays. Note that the length shown in the illustration is not up to scale. Further details regarding the estimation of the 'effective attenuation length of probing photon' are provided in the following text.

In this model, the incoming X-ray probes the sample at angle nearly perpendicular to the sample as a representation of the experimental geometry. The volume of the sample probed by the X-rays was modeled as an elliptical cylinder. The base of the cylinder corresponds to the approximate size of the beam spot (237 µm × 37 µm, as determined before the experiment), and the height represents the 'effective attenuation length of probing photons'. In this model, the 'effective attenuation length of probing photons' is defined as the distance at which the incoming photon intensity has been attenuated to (1/e = 0.367) of the initial incoming intensity ($I_0$) as it probes through the Kapton, Ti adhesion layer, Pt electrode, and electrolyte. It is important to note that the estimation of the irradiation dose absorbed by the sample is made by considering the 'effective length of *probing photons*' and not the 'effective *detection depth* of the sample' (which is approximated in detail in section S8). This is because the

irradiation dose corresponds to the mass of the sample that absorbed the radiation. Thus, the estimation of irradiation dose absorbed by the electrolyte is based on the attenuation length of probing X-ray and not the detected photons.

By using Lambert-Beer's Law, the thickness of the electrolyte that X-rays need to pass through until the intensity is attenuated to (1/e) of its initial value can be determined by using Eq.S5. The transmittances for 12 µm kapton, 5 nm Ti, and 15 nm Pt are 0.58, 0.99, and 0.91 at the X-ray energy of 2156.5 eV (the maximum energy of the XANES scans), respectively, as determined from LBL (Lawrence Berkeley Laboratory) X-ray filter transmission database [8]. By incorporating these transmittance values into Eq. S5, it can be shown that the electrolyte needs to possess a transmittance of 0.69 to attenuate the incoming X-ray intensity to (1/e) of its initial intensity. Considering the $H_3PO_3$ density of ($\rho_{5MH3PO3}$= c × M = 5 mol dm$^{-3}$ × 81.99 g mol$^{-1}$= 409.95 g dm$^{-3}$) for 5 mol dm$^{-3}$ $H_3PO_3$ electrolyte, this transmittance corresponds to approximately 7.0 µm of the electrolyte layer.

Hereafter, the electrolyte thickness required to attenuate the X-rays to this value will be referred to as the "effective attenuation length of *probing photons*". Please note that this value is slightly overestimated since it approximates the transmittance of the electrolyte layer only with the transmittance of $H_3PO_3$ for 5 mol dm$^{-3}$ $H_3PO_3$ electrolyte. In reality, the electrolyte consists of a mixture of $H_3PO_3$ and $H_2O$, and as a result, more X-rays are absorbed by the $H_2O$. This leads to a smaller effective attenuation length. Please also note that the estimation was performed for a 15 nm planar Pt electrode. For the Pt black electrode, which is approximately 10 nm thicker than planar Pt, the effective attenuation length in the electrolyte is ~6.0 µm, slightly lower than the effective attenuation length with planar Pt. Similarly, for XANES recorded on the Pt free Kapton (without planar Pt layer and Ti layer), the effective attenuation length of the probing photon is ~8.8 µm. Note that the estimation is made under assumption of flat Pt layer, and thus the estimation is less accurate for Pt black with high surface roughness. It is also worth noting, that for 5 mol dm$^{-3}$ $H_3PO_4$ or 5 mol dm$^{-3}$ $H_3PO_2$, the effective detection depth is slightly different than the $H_3PO_3$ (by around ± 0.2 µm), given that the density and the transmittance of both solutions differs slightly from $H_3PO_3$. Furthermore, it is important to emphasize that the effective attenuation length of probing photons is different from the ‘effective attenuation length of *detected photons*’, or effective *detection depth* in electrolyte. Considerations for effective detection depth in the electrolyte is discussed in section S9.

Using the model illustrated in **Figure S15**, the volume of $H_3PO_3$ ($m_{H3PO3}$) in the probed 5 mol dm$^{-3}$ $H_3PO_3$ electrolyte can be estimated by multiplying the probed electrolyte volume (π × (237/2) µm × (37/2) µm) with the effective attenuation length of probing photons in electrolyte. Subsequently mass of probed $H_3PO_3$ can be determined by multiplying the probed volume with the density of 5 mol dm$^{-3}$ $H_3PO_3$ in the electrolyte ($\rho_{5MH3PO3}$= c × M = 5 mol dm$^{-3}$ × 81.99 g mol$^{-1}$= 409.95 g dm$^{-3}$). Using a similar method, the mass of water in the electrolyte can be determined ($m_{H2O} = V_{electrolyte} \times \rho_{H2O}$). In total, the mas of electrolyte absorbing the irradiation are approximated by

$m_{H2O}$ + $m_{5MH3PO3}$. Similarly, for XANES measurement with 5 mol dm$^{-3}$ $H_3PO_2$ or 5 mol dm$^{-3}$ $H_3PO_4$, the mass of the probed $H_3PO_2$ or $H_3PO_4$, in electrolyte differs slightly from $H_3PO_3$ electrolyte: by a factor of $M(H_3PO_2)/M(H_3PO_3)$ = 66/82 = 0.8 for $H_3PO_2$, or $M(H_3PO_2)/M(H_3PO_3)$ = 98/82 = 1.2, for $H_3PO_4$.

**Table S1** illustrates the mass of probed electrolyte for each electrode and electrolyte solutions, that is shown in **Figure 1.A, 1.B, 2.A, 2.B., and 2.C** in the main text.

**Table S1.** Estimated electrolyte mass that absorbs irradiation during *in situ* P *K*-edge XANES scans shown in **Figure 1.B, 1.C, 2.A, 2.B, and 2.C.** in the main text.

| Electrode | Electrolyte | Estimated effective attenuation length of probing photons in the electrolyte (μm) | Probed electrolyte volume ($10^4$ μm$^3$) | m $H_2O$ ($10^{-8}$ g) | m $H_3PO_x$ [x = 2, 3, or 4] ($10^{-8}$ g) | m $H_2O$+ m $H_3PO_x$ ($10^{-8}$ g) |
|---|---|---|---|---|---|---|
| ‘Pt free' Kapton | $H_3PO_4$ | 8.8 | 6.0 | 6.0 | 2.9 | 8.9 |
| | $H_3PO_3$ | | | | 2.4 | 8.5 |
| | $H_3PO_2$ | | | | 1.9 | 8.0 |
| planar Pt | $H_3PO_4$ | 7.0 | 4.8 | 4.8 | 2.4 | 7.1 |
| | $H_3PO_3$ | | | | 2.0 | 6.7 |
| | $H_3PO_2$ | | | | 1.6 | 6.4 |
| Pt black | $H_3PO_4$ | 6.0 | 4.1 | 4.1 | 2.1 | 6.1 |
| | $H_3PO_3$ | | | | 1.7 | 5.8 |

Using Eq. S8 and probed electrolyte mass given in **Table S1**, the radiation dose absorbed by the 5 mol dm$^{-3}$ $H_3PO_x$ in XANES experiment (x = 2, 3, or 4) for each different electrode that is shown in **Figure 1.A, 1.B, 2.A, 2.B., and 2.C** in the main text, are approximated and shown in **Table S2**.

**Table S2.** Estimated radiation dose absorbed by electrolyte for each *in situ* P *K*-edge XANES shown in **Figure 1.B, 1.C, 2.A, 2.B, and 2.C.** in the main text. Estimations were conducted with Eq. S8, with the incoming photon energy of 2156.5 eV (maximum energy of the XANES scan). Note that for XANES of 'Pt free' Kapton|(5 mol dm$^{-3}$) $H_3PO_3$ with low dose, the incoming photon fluxes are different, since the measurement was performed in a different beamtime campaign with lower photon fluxes. Yet, due to the longer exposure time for that experiment, the radiation dose remains comparable to other measurements with lower radiation doses (e.g., XANES of planar Pt|(5 mol dm$^{-3}$) $H_3PO_3$).

| **Electrolyte (concentration: 5 mol dm$^{-3}$)** | **Electrode / subtrate** | **Irradiation dose** | **Incoming photon fluxes from beamline (photons s$^{-1}$)*** | **Transmittance of attenuating layer**** | **Exposure time, Δt (s)** | **Irradiation dose absorbed by the electrolyte, $D_T$ ($10^5$ kGy)** | **Ratio of irradiation dose relative to the high irradiation dose#** |
|---|---|---|---|---|---|---|---|
| $H_3PO_3$ | 'Pt free' Kapton | low dose | $1.9 \times 10^{11}$ | 0.58 | 402 | 1.8 | 0.38 |
| | | high dose | $7.7 \times 10^{11}$ | | 265 | 4.7 | 1 |
| | planar Pt | low dose | | 0.52 | 113 | 2.3 | 0.43 |
| | | high dose | | | 265 | 5.4 | 1 |
| | Pt black | low dose | | 0.49 | 113 | 2.5 | 0.43 |
| | | high dose | | | 265 | 6.0 | 1 |
| $H_3PO_4$ | 'Pt free' Kapton | low dose | | 0.58 | 113 | 1.9 | 0.43 |
| | | high dose | | | 265 | 4.4 | 1 |
| | planar Pt | low dose | | 0.52 | 113 | 2.2 | 0.43 |
| | | high dose | | | 265 | 5.1 | 1 |
| | Pt black | low dose | | 0.49 | 113 | 2.4 | 0.43 |
| | | high dose | | | 265 | 5.6 | 1 |
| $H_3PO_2$ | 'Pt free' Kapton | low dose | | 0.58 | 113 | 2.1 | 0.43 |
| | | high dose | | | 265 | 4.9 | 1 |
| | planar Pt | low dose | | 0.52 | 113 | 2.4 | 0.43 |
| | | high dose | | | 265 | 5.7 | 1 |

*Represents the incoming photon fluxes from the beamline as measured by the photodiode. Please note that the photon fluxes needed to calculate the radiation dose absorbed by the electrolyte (using Eq. S8), are not the incoming photon flux from the beamline, but photon fluxes that reach the electrolyte. This corresponds to the incoming photon flux from beamline ($I_0$) multiplied by the X-ray transmittance of the attenuating layer before photons reach electrolyte**. For instance, transmittance of the attenuating layer in the XANES of planar Pt|5 mol dm$^{-3}$ $H_3PO_x$ is given by 12 µm Kapton, 5 nm Ti, and 15 nm Pt electrode, which corresponds to ($0.58 \times 0.99 \times 0.91 \times I_0$ = 0.52 $I_0$), as previously discussed. #For measurements with the same photon fluxes, the ratio of radiation dose is similar to the ratio of exposure time. Note that the ratio is calculated for the same electrolyte (e.g., the ratio of irradiation dose for

planar Pt|$H_3PO_3$ with low irradiation dose is made in comparison to the planar Pt|$H_3PO_3$ with high irradiation dose).

In **Table S2**, it is shown that the mass of electrolyte absorbing irradiation in the XANES measurements for different electrodes is comparable. For instance, the percentage difference between the radiation dose absorbed by the electrolyte during XANES measurements with planar Pt and Pt black is $\left(\frac{D_{T(planar\,Pt} - D_{T\,(Pt\,black)}}{(D_{T\,(planar\,Pt)} + D_{T\,(Pt\,black)})/2}\right) \times 100\% = 10.1\%$. This similarity arises because, although the effective attenuation length of probed photons differs for various electrodes (resulting in different mass of electrolyte that is absorbing the irradiation), the intensity of photons reaching the electrolyte also varies among electrodes. For instance, despite the smaller effective attenuation length of probing photons in the XANES measurements recorded on Pt black compared to planar Pt, the intensity of photons reaching the electrolyte is also smaller in XANES measurements with Pt black. This is due to thicker Pt black attenuating more incoming X-rays than the thinner planar Pt, as reflected in the transmittance of the attenuating layer column in **Table S2**.

Additionally, please note that in these experiments, shorter time exposure for several of the XANES measurements with low dose was achieved by rapid opening and closing of a valve during the XANES experiments (to be specific: valve is closed during the deadtime of the XANES measurements). It is interesting to note that the recorded electrode $E_{OCP}$ can be used to determine the radiation exposure time during the experiment with low dose achieved with the aforementioned method. **Figure S16** shows the example $E_{OCP}$ recording of Pt black|5 mol dm$^{-3}$ during three sequential XANES experiments.

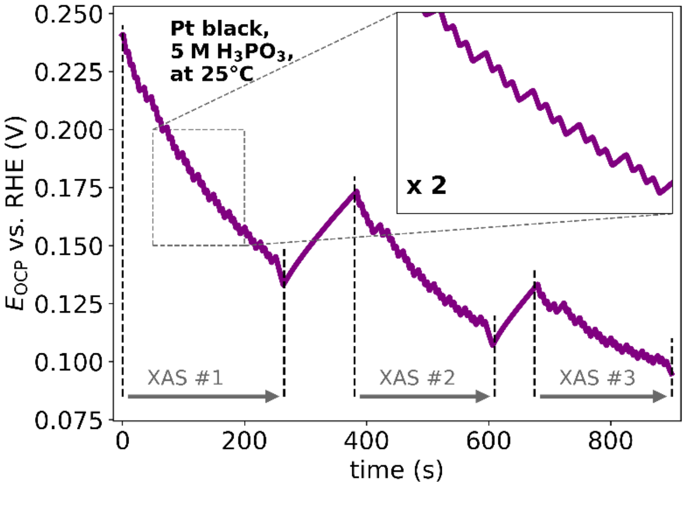


**Figure S16.** $E_{OCP}$ recording during three sequential XANES scans of Pt black|(5 mol dm$^{-3}$) $H_3PO_3$. The inset plot provides a magnified view of the $E_{OCP}$ during XANES, showing rapid fluctuations as the valve is quickly closed and opened to minimize the radiation dose. Measurements were conducted at the temperature of 25 °C.

During the XANES (i.e., synchrotron radiation illumination), there is a general drop of $E_{OCP}$, hinting at $H_2$ generation, as is detailed in the main text. In between XANES measurements, the valve was closed as the measurement spot was changed and the monochromator energy was set back to the starting energy of the XANES scan. During this time (i.e., ~265 s to 380 s and 610 s to 675 s), the $E_{OCP}$ increased, since $H_2$ was not generated anymore, while the $H_2$ previously generated during illumination, diffuse away from the Pt electrode. For a similar reason, during the XANES, $E_{OCP}$ repeatedly increases and decreases, as the valve rapidly opens and closes, as shown in the inset plot of **Figure S16**. This observation indirectly shows the time for which the sample was exposed to radiation (i.e., the time in which $E_{OCP}$ is decreasing) and the time in which radiation was blocked off the sample (the time in which $E_{OCP}$ is increasing). This $E_{OCP}$ observation was used to estimate exposure time for the estimation of the radiation dose.

In addition, a comparison of the irradiation dose between the current experiment and two previous investigations (Ref. [4] and Ref. [11]), is provided in the following.

For the XANES results of aqueous $H_3PO_3$ presented in Ref. [4], measurements were conducted at the HiKE end-station [13] located at the bending magnet beamline of KMC-1 [14], using a Si (111) double crystal monochromator. With this configuration, the incoming photon fluxes to the end station were approximately $2×10^{10}$ photons $s^{-1}$ at the excitation energy of 2.1 keV. The scan duration for XANES scans (from 2120 eV to 2200 keV) was approximately 40 minutes (and only ~15 minutes for XANES scan to reach the energy of 2160 eV, which is used for this experiment). Based on the estimation of dose using Eq. S8, the dose for the measurements in the mentioned study was approximately $1.1 × 10^5$ kGy, which is only 65% of the dose used for the "low dose" XANES experiment used in the current study.

For the *in situ* AP-HAXPES data presented in Ref. [11], experiments were performed at the SpAnTeX end-station [15], which is also located at the bending magnet KMC-1 beamline. A Si (111) crystal monochromator was used for these experiments, resulting in incoming photon fluxes of approximately $9×10^{10}$ photons $s^{-1}$ at the excitation energy of 3 keV. This flux counts for roughly 12% of the incoming fluxes used in the current XANES study using the CPMU17 EMIL undulator beamline (~$7.7 × 10^{11}$ photons $s^{-1}$). Furthermore, at this excitation energy, the X-ray transmittance of $H_2O$ and $H_3PO_3$ is much larger than the transmittance at 2.15 keV. The transmittance for 1 µm layer of $H_2O$ and $H_3PO_3$ at 3 keV are 0.98 and 0.91, respectively, whereas at 2.16 keV, the transmittance for $H_2O$ and $H_3PO_3$ are 0.95 and 0.8 respectively. Given that the photon absorbance is proportional to (1 – transmittance), the X-ray absorbance by the $H_3PO_3$ at 3 keV is approximately 9%, while the X-ray absorbance at 2.15 keV is ~20%. This indicate that lower dose of X-ray is absorbed by the electrolyte for the *in situ* AP-HAXPES experiment (i.e., at 3 keV) compared to the current XANES experiments (i.e., at ~2.15 keV). Additionally, this measurement is performed at ambient pressure between 18 mbar to 22 mbar of water vapor. Hence, some of the incoming X-rays will also be absorbed by the water vapor in the chamber, resulting in even lower photon flux to the sample. Consequently, this translates to a

lower dose of X-ray for this study. In fact, the averaged $E_{OCP}$ of Pt|aqueous $H_3PO_3$ electrolyte measured during the *in situ* AP-HAXPES experiments were ~0.4 $V_{RHE}$ and 0.47 $V_{RHE}$, for 5 mol $dm^{-3}$ and 1 mol $dm^{-3}$ electrolytes, respectively [4]. This value is very similar to the $E_{OCP}$ of the same system measured without irradiation (see the initial $E_{OCP}$ given in **Figure 2.D** in the main text). If a strong radiation-induced effect occurs, then the $E_{OCP}$ should decrease to a lower value (e.g., closer to ~0.1 $V_{RHE}$), as observed in the current experiment. Therefore, these $E_{OCP}$ values further indicate that very small radiation effects occurred during the *in situ* AP-HAXPES experiment.

## 13. Additional P *K*-edge XANES of aqueous $H_3PO_3$ with different incoming photon fluxes

To further validate the dependency of the increase in spectral weight corresponding to P(+5) compounds observed on the P *K*-edge XANES of 5 mol dm$^{-3}$ with different radiation doses $H_3PO_3$ (as shown in **Figure 2.B** in the main text), additional XANES measurements were conducted with varying incoming photon fluxes (which are proportional to photon fluxes reaching electrolyte solution, as indicated by Eq. S8), as depicted in **Figure S17**.

The incoming photon fluxes ($I_0$) were determined just before each XANES measurement using a photodiode (ODD-AXU-010, Optodiode) placed in the trajectory of the incoming X-rays near the probed Pt|aqueous electrolyte interface, using a similar method as detailed in section S12. The measurements with lower fluxes were achieved by detuning the undulator gap of the beamline.

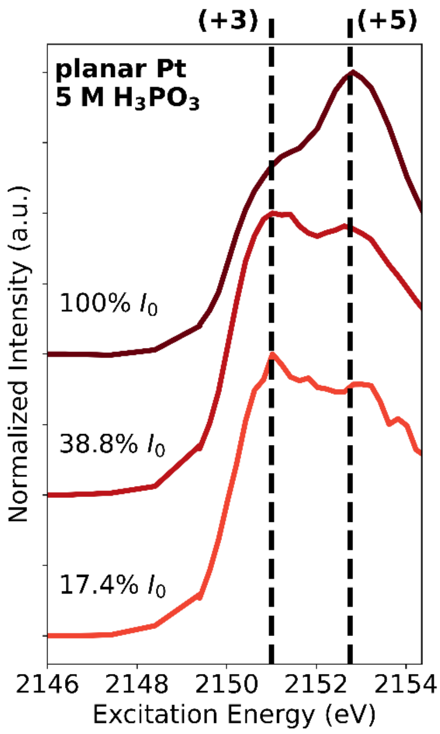


**Figure S17.** P *K*-edge XANES of planar Pt|5 mol dm$^{-3}$ (5M) $H_3PO_3$, recorded under different incoming photon fluxes ($I_0$). $I_0$ corresponds to $7.71\times10^{11}$ photon s$^{-1}$. Measurements were conducted at the temperature of 25 °C.

As observed in **Figure S17**, with an increase in photon flux, a higher spectral weight corresponding to P compounds with an oxidation state of P (+5) is evident, consistent with the trend revealed in the main text.

## 14. Additional $E_{OCP}$ recording of planar Pt|(5 mol dm$^{-3}$) $H_3PO_3$ under synchrotron irradiation with varying incoming photon fluxes and $E_{OCP}$ under repeated application of irradiation and without irradiation

To provide further confirmation that the drop in $E_{OCP}$ values of planar Pt|5 mol dm$^{-3}$ $H_3PO$, as observed in **Figure 2.D** in the main text, is indeed dependent on the radiation dose (i.e., proportional to photon fluxes, as shown by Eq. S8), additional $E_{OCP}$ recordings were conducted with a planar Pt|(5 mol dm$^{-3}$) $H_3PO_3$ sample under synchrotron irradiation (SR), using three different incoming photon fluxes (see **Figure S18**). A similar method as described in Section S13 was employed to determine the photon fluxes and to reduce the incoming photon fluxes for these experiments.

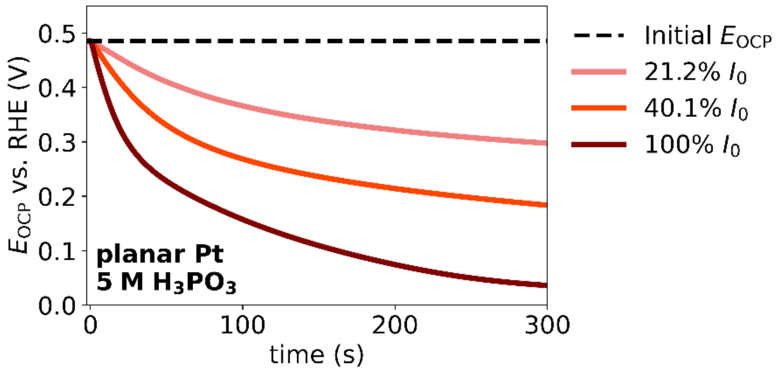


**Figure S18.** $E_{OCP}$ of planar Pt electrode|5 mol dm$^{-3}$ (5M) $H_3PO_3$ under different incoming photon fluxes ($I_0$). $I_0$ corresponds to $7.71 \times 10^{11}$ photon s$^{-1}$. Measurements were conducted at the temperature of 25 °C.

As depicted in **Figure S18,** a larger drop of $E_{OCP}$ values was observed during irradiation with higher fluxes (∝ radiation doses), similar to the trend observed in the main text.

Additionally, to confirm that $E_{OCP}$ of planar Pt|5 mol dm$^{-3}$ $H_3PO_3$ is increasing when radiation is blocked right after irradiation is applied to the system (towards the initial $E_{OCP}$ without irradiation), $E_{OCP}$ of planar Pt electrode|5 mol dm$^{-3}$ $H_3PO_3$ under repeated application of synchrotron irradiation and without irradiation, is recorded. This experiment is shown in **Figure S19**.

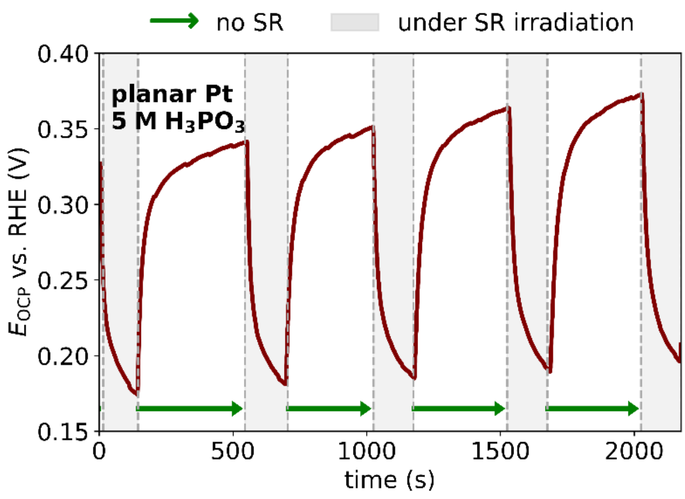


**Figure S19.** $E_{OCP}$ of planar Pt electrode|5 mol dm$^{-3}$ (5M) $H_3PO_3$ under repeated application of synchrotron irradiation (SR, region shaded in gray) and without irradiation (illustrated by green arrow). An increase of $E_{OCP}$ is observed in the period without irradiation. The experiment was performed at a temperature of 25 °C.

**Figure S19** illustrates the reproducible increase of $E_{OCP}$ (towards the initial $E_{OCP}$ of the measurement, as given in **Figure 2.D** in the main text) at the period at which radiation is blocked, right after synchrotron irradiation to the planar Pt|5 mol dm$^{-3}$ $H_3PO_3$.

## 15. *E*$_{OCP}$ drop and $H_2$ partial pressure estimation from *E*$_{OCP}$ recordings of planar Pt|(5 mol dm$^{-3}$) $H_3PO_3$ and planar Pt|(5 mol dm$^{-3}$) $H_3PO_4$ under different irradiation doses

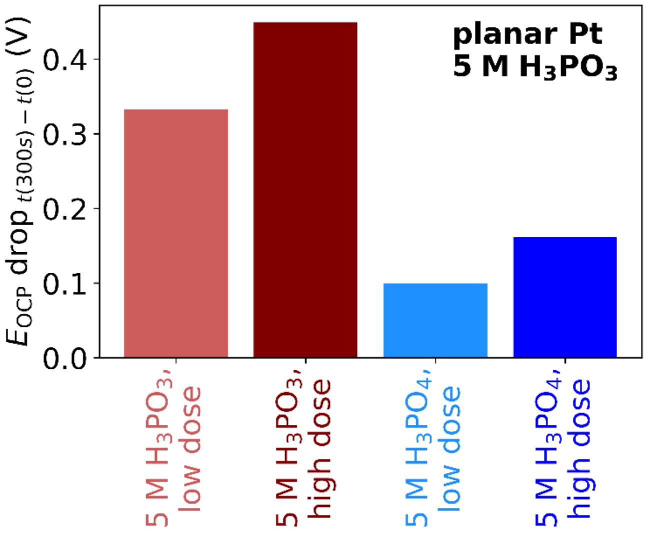


**Figure S20.** *E*$_{OCP}$ value drop derived from the *E*$_{OCP}$ recording during the *in situ* P *K*-edge XANES of planar Pt|(5 mol dm$^{-3}$) $H_3PO_3$ and planar Pt|(5 mol dm$^{-3}$) $H_3PO_4$ under different irradiation doses after 300 s, as given in **Figure 2.D** in the main text. Measurements were recorded at the temperature of 25 °C

As shown in **Figure S20**, the *E*$_{OCP}$ value drop observed in 5 mol dm$^{-3}$ $H_3PO_3$ is bigger than the decrease of *E*$_{OCP}$ in 5 mol dm$^{-3}$ $H_3PO_4$, for both high dose and low dose measurements. Assuming that this drop in *E*$_{OCP}$ value is caused by the presence of $H_2$ in the vicinity of the Pt electrode and the subsequent 2 $H^+$ + 2 $e^-$ ⇆ $H_2$ reaction on the electrode to achieve equilibrium (as detailed in the main text), the activity of $H_2$ can be approximately estimated by solving Nernst equation for the activity of $H_2$, as given in Eq. S9.

$$a_{H_2} = e^{(E^{\circ} - E_{eq \cong OCP})\frac{zF}{RT}} (a_{H^+})^2 \qquad \text{Eq. S9}$$

$a_{H2}$ corresponds to the activity of $H_2$. $E_{Eq}$ represents the equilibrium potential of the reaction, in this case, the *E*$_{OCP}$ after the drop where a quasi-steady-state is obtained (e.g., when the *E*$_{OCP}$ is almost constant, in this case, *E*$_{OCP}$ recorded at t = 300 s). $E^{\circ}$ is the standard redox potential of the $H^+/H_2$ (0 $V_{RHE}$), *R* is the universal gas constant (*R* = 8.31 J $K^{-1}$ $mol^{-1}$). *T* corresponds to the temperature (~ 297 K). *F* is the Faraday constant (F = 9.648 × $10^4$ C $mol^{-1}$) and z is the number of transferred electrons (z = 2 for HER/HOR). $a_{H+}$ corresponds to the activity of $H^+$ in the electrolyte. Given that the 5 mol dm$^{-3}$ $H_3PO_3$ possesses a pH of 0.32, the activity of $H^+$ was determined to be: $a_{H^+} = 10^{-pH} = 0.47$.

The activity of gasses is correlated to the gasses' partial pressure via Eq. S10:

$$a_{H_2} = \frac{p(H_2)}{p^{standard}} \qquad \text{Eq. S10}$$

where *p*($H_2$) is the partial pressure of $H_2$ (in Pa) and $p^{standard}$ is the standard pressure ($10^5$ Pa).

**Table S3** shows the estimation of partial pressure $H_2$ during experiments with 5 mol dm$^{-3}$ $H_3PO_3$ and 5 mol dm$^{-3}$ $H_3PO_4$, determined from Eq. S9 and S10.

**Table S3.** Estimated partial pressure of $H_2$ [$p(H_2)$] during the $E_{OCP}$ recording of 5 mol dm$^{-3}$ $H_3PO_3$ with different irradiation doses, calculated from Eq. S9 and S10.

| **Sample** | **Irradiation dose** | **$p(H_2)$ (Pa) *** |
|---|---|---|
| Planar Pt\|5 mol dm$^{-3}$ $H_3PO_3$ | low dose | $2.9 \times 10^{-1}$ |
| | high dose | $1.3 \times 10^{3}$ |
| Planar Pt\|5 mol dm$^{-3}$ $H_3PO_4$ | low dose | $3.4 \times 10^{-10}$ |
| | high dose | $9.6 \times 10^{-8}$ |

*Please note that this estimation was made given the assumption that the $E_{OCP}$ drop solely corresponds to the presence of $H_2$ and subsequent $H^+/H_2$ equilibrium at the Pt surface.

As shown in **Table S3**, the $p(H_2)$ in 5 mol dm$^{-3}$ $H_3PO_3$ under a high irradiation dose is ~ $4 \times 10^3$ times bigger than the $p(H_2)$ under a low irradiation dose. This confirms that the $H_2$ generation under high irradiation doses is indeed higher than the $H_2$ generation in low doses.

However, the $p(H_2)$ that were estimated for the experiment with 5 mol dm$^{-3}$ $H_3PO_4$ electrolyte, are significantly lower than the corresponding values in 5 mol dm$^{-3}$ $H_3PO_3$. Especially for the low dose measurement, the estimated $p(H_2)$ is very low, and as such, its physical meaning is questionable. For the measurement with 5 mol dm$^{-3}$ $H_3PO_3$ electrolyte, there is likely a high formation of $H_2$. This leads to the a strong drop of $E_{OCP}$ in 5 mol dm$^{-3}$ $H_3PO_3$ electrolyte, since the $E_{OCP}$ is then predominantly influenced by the 2 $H^+$ + 2 $e^-$ $\leftrightarrows$ $H_2$ reaction. For measurement in the 5 mol dm$^{-3}$ $H_3PO_4$ electrolyte, the effect from the aforementioned reaction is not the dominant factor, as the presence of $H_2$ is likely much lower than that in the experiment with $H_3PO_3$. Thus, the estimated $p(H_2)$ value is only "accurate" for the 5 mol dm$^{-3}$ $H_3PO_3$ electrolyte, where 2 $H^+$ + 2 $e^-$ $\leftrightarrows$ $H_2$ reaction majorly influences the $E_{OCP}$, and is less accurate for the 5 mol dm$^{-3}$ $H_3PO_4$ electrolyte.

Yet, even though the estimation of $p(H_2)$ for the aqueous $H_3PO_4$ electrolyte is imprecise, it is important to note that the high drop of $E_{OCP}$ in 5 mol dm$^{-3}$ $H_3PO_3$ compared to 5 mol dm$^{-3}$ $H_3PO_4$ (in both irradiation doses, see **Figure S20**), indicates a much stronger activity of $H_2$ in $H_3PO_3$ electrolyte (i.e. much higher $H_2$ formation). Assuming that the drop of $E_{OCP}$ in 5 mol dm$^{-3}$ $H_3PO_4$ was caused by the $H_2$ formation due to the radiolysis of water (as detailed in the main text), a similar drop of $E_{OCP}$ should be seen in 5 mol dm$^{-3}$ $H_3PO_3$, given that both solutions have the same concentration of $H_2O$. This suggests that another process is also taking place in the aqueous $H_3PO_3$ electrolyte under irradiation, which results in $H_2$ generation. This further indicates the possibility of radiation-induced oxidation of $H_3PO_3$ to $H_3PO_4$, generating $H_2$ alongside it (see details in the main text).

## 16. Stability assessment of aqueous $H_3PO_2$ with and without the presence of Pt through ion-exchange chromatography

To evaluate the stability of aqueous $H_3PO_2$, ion-exchange chromatography (IEC) experiments were conducted on a set of 10 mmol dm$^{-3}$ $H_3PO_2$ that had been aged for various durations, both in the presence and absence of Pt catalysts, as depicted in **Figure S21**. The experimental setup used for these investigations was the same as described in Section 2.3 of the main text, with the eluent of 20 mmol dm$^{-3}$ KOH being employed.

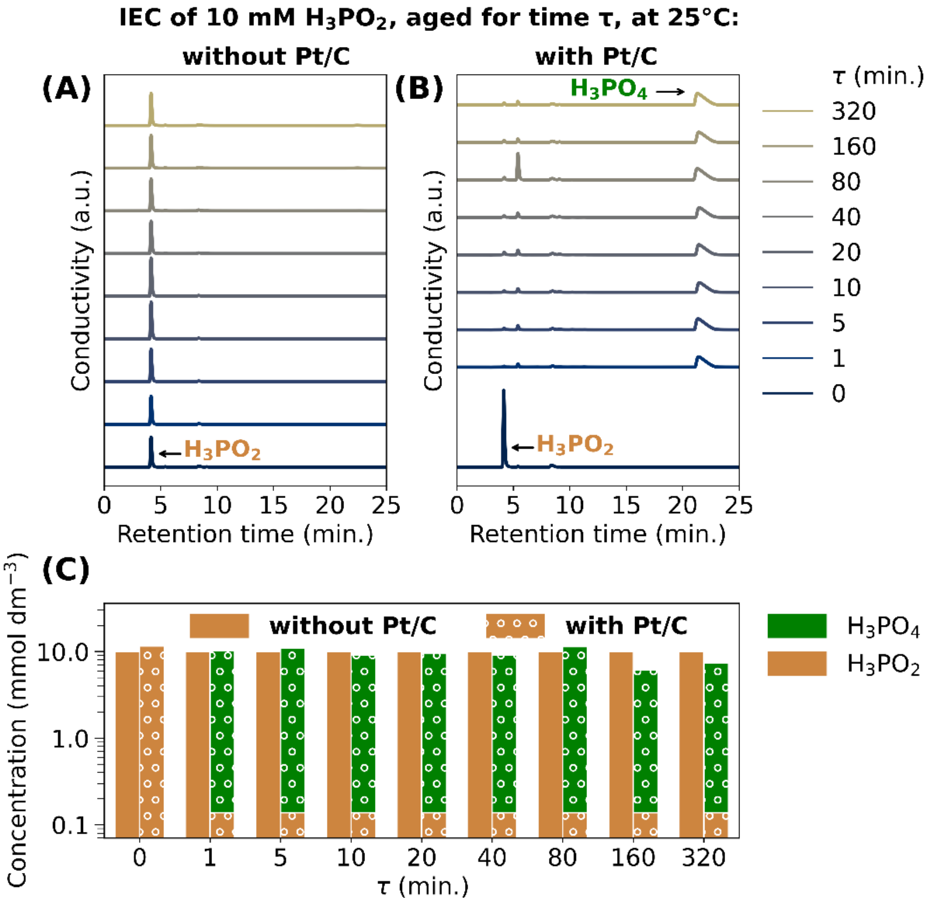


**Figure S21.** Ion exchange chromatography (IEC) performed on a 10 mmol dm$^{-3}$ (10 mM) $H_3PO_2$ electrolyte that has been aged for a specific duration ($\tau$) at 25 °C. In **(A)**, no Pt/C catalysts were dispersed in the electrolyte before aging, while in **(B)**, 50 mg of Pt/C catalysts (40 wt% Pt) were dispersed before aging. **(C)** Concentration of $H_3PO_2$ and $H_3PO_4$, obtained from the IEC results in panels (A) and (B). A pronounced oxidation of $H_3PO_2$ to $H_3PO_4$ is observed after just 1 minute of aging.

**Figure S21** illustrates a significant oxidation of $H_3PO_2$ to $H_3PO_4$ within 1 minute of aging in the aqueous 10 mmol dm$^{-3}$ $H_3PO_2$. By comparing the IEC results of 10 mmol dm$^{-3}$ $H_3PO_2$ with those of 10 mmol dm$^{-3}$ $H_3PO_3$ (see **Figure 4.E** in the main text), it becomes evident that the conversion of $H_3PO_2$ to $H_3PO_4$ occurs at a faster rate compared to the conversion of $H_3PO_3$ to $H_3PO_4$ in electrolytes of the same concentration. **Table S4** shows a comparison of the electrolyte aging time until 90% of the initial

$H_3PO_2$ or $H_3PO_3$ concentration has been converted to $H_3PO_4$, as derived from the IEC analysis of those 10 mmol dm$^{-3}$ aqueous solution in the presence of Pt/C catalysts at 25 °C (from **Figure S21.C** and Figure **Figure 4.E**).

**Table S4.** Comparison of the electrolyte aging time until 90% of the initial $H_3PO_2$ or $H_3PO_3$ electrolyte concentration has been converted to $H_3PO_4$, based on the IEC analysis of 10 mmol dm$^{-3}$ aqueous $H_3PO_2$ or $H_3PO_3$ in the presence of Pt/C catalysts at 25 °C (from **Figure S12.C** and Figure **Figure 4.D**).

| **Electrolyte** | **Aging time until IEC shows that 90% of initial $H_3PO_2$ or $H_3PO_3$ electrolyte concentration has been converted to $H_3PO_4$ (min.)** |
|---|---|
| 10 mmol dm$^{-3}$ $H_3PO_2$ with Pt/C dispersion | 1 |
| 10 mmol dm$^{-3}$ $H_3PO_3$ with Pt/C dispersion | 80 |

As shown in **Table S4**, there is a significantly faster conversion of $H_3PO_2$ to $H_3PO_4$ in an aqueous solution, compared to the conversion rate of aqueous $H_3PO_3$ to $H_3PO_4$ at the same concentration. This highlights the inherent instability of aqueous $H_3PO_2$ in contrast to $H_3PO_3$. These findings further support the observed high spectral weight corresponding to P(+5) compounds (e.g. $H_3PO_4$) in the XANES spectra of aqueous $H_3PO_2$ compared to $H_3PO_3$ (as shown in **Figure 2.B** and **2.C** in the main text).

## 17. Theoretical estimation of $H_3PO_4$ generation in 5 mol dm$^{-3}$ $H_3PO_3$ during positive potentials application on the Pt black electrodes

For further insight into the increasing XANES spectral weight corresponding to P(+5) compounds of Pt black|(5 mol dm$^{-3}$) $H_3PO_3$ at various positive electrode potentials, a rough estimation of the maximum number of $H_3PO_4$ moles generated by electrochemical oxidation of $H_3PO_3$ to $H_3PO_4$ via Eq. 5 (in the main text) during XANES experiments was made. Estimations were made by the Faraday law of electrolysis for the reaction product's number of moles, given in the following Eq. S11.

$$n = \frac{A_{geo}}{Fz} \int j_{geo}\, dt \qquad \text{Eq. S11.}$$

Here F is the Faraday constant. z is the number of electrons transferred in the reaction and is equal to z = 2 for the electrochemical oxidation of $H_3PO_3$, as given by Eq. 5 in the main text. $j_{geo}$ corresponds to the geometrical current densities recorded in this experiment (shown in the inset plot of **Figure 3.A** in the main text), and $A_{geo}$ represents the geometrical area of the electrode in contact with the electrolyte ($A_{geo} = 0.502$ cm$^2$).

**Figure S22** illustrates the number of moles of product generated during XANES with Pt black, as estimated with Eq. S11.

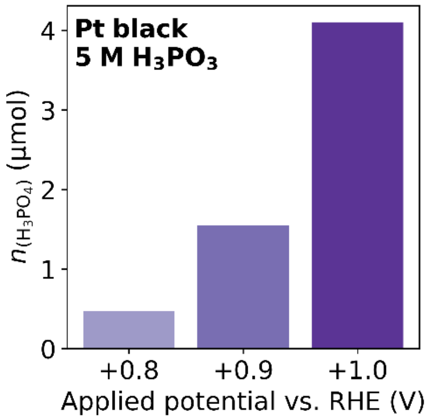


**Figure S22.** Estimation of the number of mole of oxidation products: $H_3PO_4$, generated during P *K*-edge XANES with Pt black|(5 mol dm$^{-3}$) $H_3PO_3$. The estimation assumes 100% Faradaic efficiency for the electrochemical oxidation of $H_3PO_3$ to $H_3PO_4$ (see Eq. 5 in the main text)

This estimation shows that at more positive potentials, a higher formation of $H_3PO_4$ is observed, which aligns with the increasing spectral weight corresponding to P (+5) compounds in XANES (**Figure 3.B** in the main text). Please note that this estimation was made under the assumption of 100% Faradaic efficiency of the electrochemical oxidation of $H_3PO_3$ to $H_3PO_4$, as given by Eq. S11. Additionally, it is noteworthy that the assumption was made in static condition (i.e., no diffusion). Moreover, the electrolyte was continuously flown during measurements (flow rate: 0.05 ml min$^{-1}$, in the reactor chamber of ~750 µl). Thus, this approximation shows the maximum generation of $H_3PO_4$.

## 18. P *K*-edge XANES of 5 mol dm$^{-3}$ $H_3PO_3$ on planar Pt electrode, alongside the CV and CA profile during the XANES measurements

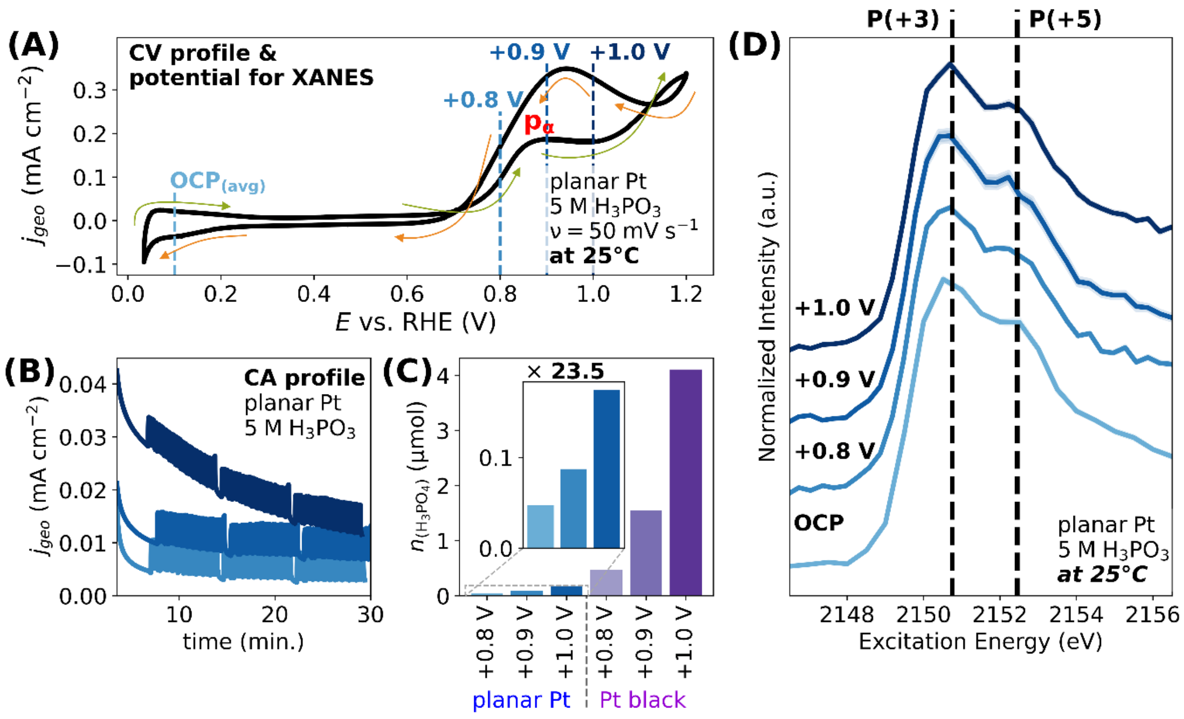


**Figure S23. (A)** CV and **(B)** Chronoamperometry (CA) profile of the planar Pt|(5 mol dm$^{-3}$) $H_3PO_3$ system at 25 °C, during the *in situ* P *K*-edge XANES measurements. pα denotes the potential corresponding to the maximum current density of electrochemical oxidation of $H_3PO_3$ to $H_3PO_4$ via Eq. 5 given in the main text. The green arrows illustrate the current density response during the positive-going potential sweep, while the orange arrows show the current density response during the negative-going potential sweep. The slight oscillation response of current density observed during the CA is attributed to the rapid valve opening/closing utilized during the XANES experiment to minimize irradiation dose. **(C)** Comparison of the amount of theoretically estimated oxidation products, i.e., $H_3PO_4$, generated during XANES with 5 mol dm$^{-3}$ $H_3PO_3$ on planar Pt *vs* Pt black. Estimations were made by the method detailed in section 12 of the SI. **(D)** Respective P *K*-edge XANES spectra for the different potential values shown in panel (A). A negligible spectral change is observed between the XANES spectra recorded with different potentials.

**Figure S23.A** and **S23.B** present the cyclic voltammetry (CV) and chronoamperometry (CA) profiles during XANES of 5 mol dm$^{-3}$ $H_3PO_3$ on a planar Pt electrode. The observed current density is much lower compared to the measurements on Pt black, primarily due to the smaller surface area of the planar Pt electrode (around 5 times smaller, as detailed in Section S2). The corresponding theoretical estimation of generated $H_3PO_4$ during these measurements indicates that the production of $H_3PO_4$ in these experiments is about 20 times lower than in experiments with Pt black. The theoretical estimation is performed using the same method detailed in Section S17. The respective P *K*-edge XANES spectra

show a negligible spectral change, suggesting that the effect arising from the planar Pt electrode is very small compared to the signal originating from the bulk of the electrolyte.

**19. P *K*-edge XANES of 5 mol dm$^{-3}$ $H_3PO_3$ on planar Pt electrode at 75 °C, under application of positive potential bias**

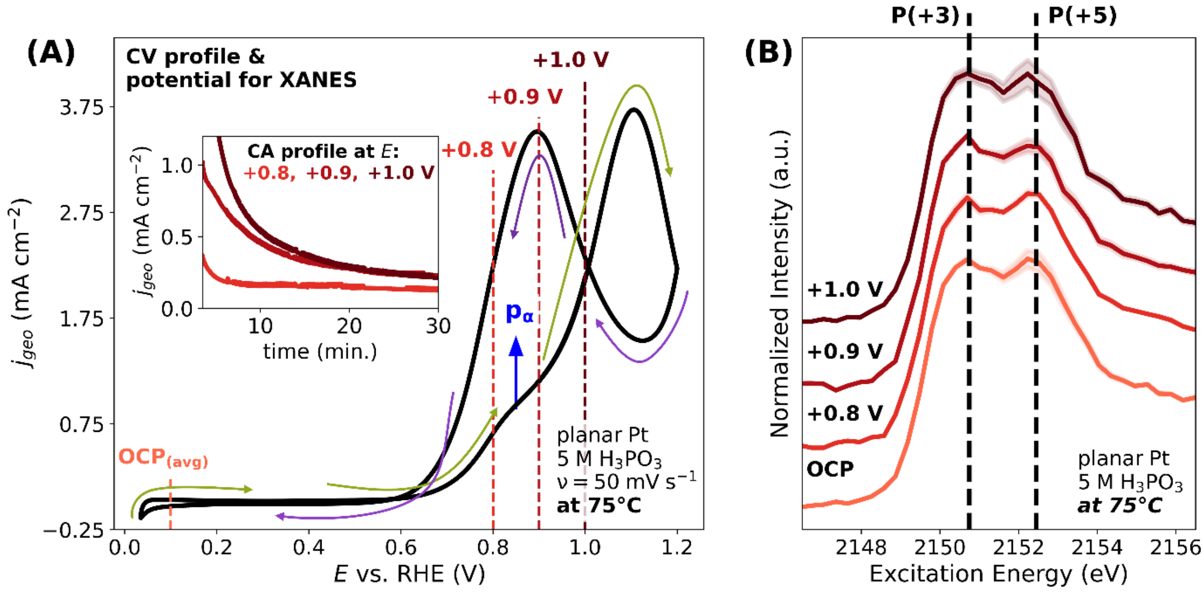


**Figure S24.** **(A)** CV recorded with a planar Pt electrode and 5 mol dm$^{-3}$ $H_3PO_3$ at 75 °C, using the scan rate of 50 mV s$^{-1}$. p$\alpha$ denotes the maximum current density of $H_3PO_3$ electrochemical oxidation to $H_3PO_4$ according to Eq. 5 in the main text. The green arrows illustrate the current density response during the positive-going potential sweep, while the purple arrows show the current density response during the negative-going potential sweep. The dashed lines correspond to the potential applied during P *K*-edge XANES experiments. The inset plot illustrates the CA profile during the potential applications. **(B)** Respective *in situ* P *K*-edge XANES during the potential applications and at $E_{OCP}$. The shaded region on the spectra represents the standard deviation between the sequential XANES measurements. A negligible spectral change was observed over different potential applications.

XANES spectra recorded under different potentials exhibit negligible spectral changes (see **Figure S24.B**). However, it is noteworthy that the magnitude of the current density drawn during potential application (see inset plot of **Figure S24.A**) is comparable to the recorded current density of the Pt black electrode (see **Figure 3** in the main text), which shows a slight spectral change upon potential application. This observation further supports the notion that at high temperatures, the combined effect of heat and radiation dominates over the influence of the planar Pt electrode and heat.

## 20. P *K*-edge XANES of aqueous $H_3PO_3$ solutions of different concentrations on planar Pt under high radiation doses

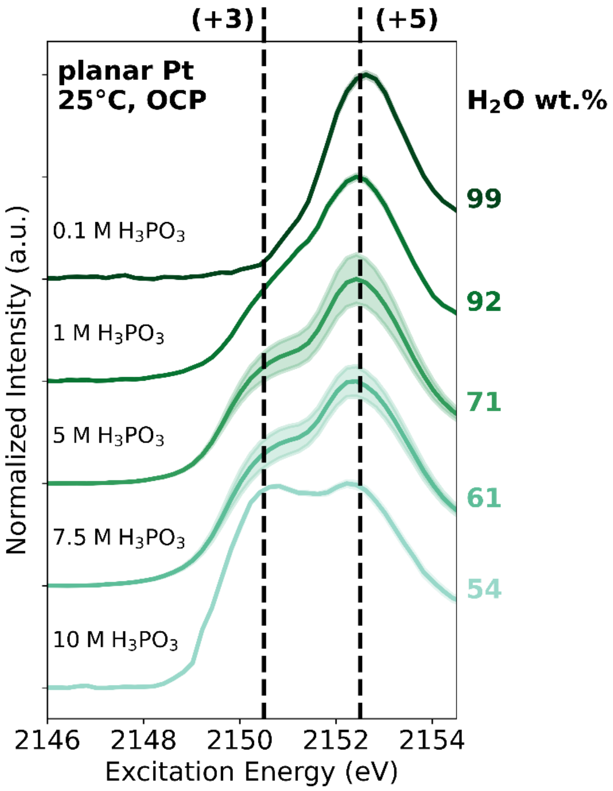


**Figure S25.** P *K*-edge XANES of planar Pt|aqueous $H_3PO_3$ with different concentrations, ranging from 0.1 mol dm$^{-3}$ (0.1 M) $H_3PO_3$ to 10 mol dm$^{-3}$ (10 M) $H_3PO_3$. The spectra of electrolytes containing higher concentrations of $H_2O$ (i.e., higher $H_2O$ wt.%), exhibit a higher spectral weight corresponding to P compounds with the oxidation state of (+5). For visualization, each spectrum is normalized to the maximum intensity of the spectrum. Measurements were performed at the temperature of 25 °C.

Although higher radiation doses led to more pronounced oxidation overall, a similar trend was observed compared to XANES spectra recorded with low radiation doses (**Figure 5** of the main text): Spectra recorded with higher water concentration (i.e., higher $H_2O$ wt.%) exhibited prominently stronger spectral weight corresponding to P(+5) compounds (e.g., $H_3PO_4$).

## 21. References


(1) Wei, C.; Sun, S.; Mandler, D.; Wang, X.; Qiao, S. Z.; Xu, Z. J. Approaches for Measuring the Surface Areas of Metal Oxide Electrocatalysts for Determining Their Intrinsic Electrocatalytic Activity. *Chem. Soc. Rev.* **2019**, *48* (9), 2518–2534. https://doi.org/10.1039/C8CS00848E.

(2) Sheng, W.; Myint, M.; Chen, J. G.; Yan, Y. Correlating the Hydrogen Evolution Reaction Activity in Alkaline Electrolytes with the Hydrogen Binding Energy on Monometallic Surfaces. *Energy Environ. Sci.* **2013**, *6* (5), 1509. https://doi.org/10.1039/c3ee00045a.

(3) Pech-Pech, I. E.; Gervasio, D. F.; Godínez-Garcia, A.; Solorza-Feria, O.; Pérez-Robles, J. F. Nanoparticles of Ag with a Pt and Pd Rich Surface Supported on Carbon as a New Catalyst for the Oxygen Electroreduction Reaction (ORR) in Acid Electrolytes: Part 1. *Journal of Power Sources* **2015**, *276*, 365–373. https://doi.org/10.1016/j.jpowsour.2014.09.112.

(4) Wibowo, R. E.; Garcia-Diez, R.; van der Merwe, M.; Duarte-Ruiz, D.; Ha, Y.; Félix, R.; Efimenko, A.; Bystron, T.; Prokop, M.; Wilks, R. G.; Bouzek, K.; Yang, W.; Cocchi, C.; Bär, M. Core-Level Spectroscopy with Hard and Soft X-Rays on Phosphorus-Containing Compounds for Energy Conversion and Storage. *J. Phys. Chem. C* **2023**, *127* (42), 20582–20593. https://doi.org/10.1021/acs.jpcc.3c04704.

(5) Krause, M. O.; Oliver, J. H. Natural Widths of Atomic *K* and *L* Levels, *K* α X-ray Lines and Several *K L L* Auger Lines. *Journal of Physical and Chemical Reference Data* **1979**, *8* (2), 329–338. https://doi.org/10.1063/1.555595.

(6) Newville, M. 2. Fundamentals of XAFS. In *Spectroscopic Methods in Mineralogy and Material Sciences*; Henderson, G., Neuville, D., Downs, R., Eds.; DE GRUYTER, 2014; pp 33–74. https://doi.org/10.1515/9781614517863.33.

(7) *X-Ray Data Booklet*; Lawrence Berkeley National Laboratory, University of California, 2001.

(8) *Filter Transmission*. https://henke.lbl.gov/optical_constants/filter2.html (accessed 2023-06-14).

(9) Henke, B. L.; Gullikson, E. M.; Davis, J. C. X-Ray Interactions: Photoabsorption, Scattering, Transmission, and Reflection at E = 50-30,000 EV, Z = 1-92. *Atomic Data and Nuclear Data Tables* **1993**, *54* (2), 181–342. https://doi.org/10.1006/adnd.1993.1013.

(10) *Polyimide*. https://www.mit.edu/~6.777/matprops/polyimide.htm (accessed 2023-12-07).

(11) Wibowo, R. E.; Garcia-Diez, R.; Bystron, T.; Prokop, M.; van der Merwe, M.; Arce, M. D.; Jiménez, C. E.; Hsieh, T.-E.; Frisch, J.; Steigert, A.; Favaro, M.; Starr, D. E.; Wilks, R. G.; Bouzek, K.; Bär, M. Oxidation of Aqueous Phosphorous Acid Electrolyte in Contact with Pt Studied by X-Ray Photoemission Spectroscopy. *ACS Appl. Mater. Interfaces* **2023**, *15* (44), 51989–51999. https://doi.org/10.1021/acsami.3c12557.

(12) Huart, L.; Nicolas, C.; Kaddissy, J. A.; Guigner, J.-M.; Touati, A.; Politis, M.-F.; Mercere, P.; Gervais, B.; Renault, J.-P.; Hervé du Penhoat, M.-A. Soft X-Ray Radiation and Monte Carlo Simulations: Good Tools to Describe the Radiation Chemistry of Sub-KeV Electrons. *J. Phys. Chem. A* **2020**, *124* (10), 1896–1902. https://doi.org/10.1021/acs.jpca.9b10539.

(13) Gorgoi, M.; Svensson, S.; Schäfers, F.; Öhrwall, G.; Mertin, M.; Bressler, P.; Karis, O.; Siegbahn, H.; Sandell, A.; Rensmo, H.; Doherty, W.; Jung, C.; Braun, W.; Eberhardt, W. The High Kinetic Energy Photoelectron Spectroscopy Facility at BESSY Progress and First Results. *Nuclear Instruments and Methods in Physics Research Section A: Accelerators, Spectrometers, Detectors and Associated Equipment* **2009**, *601* (1–2), 48–53. https://doi.org/10.1016/j.nima.2008.12.244.

(14) Schaefers, F.; Mertin, M.; Gorgoi, M. KMC-1: A High Resolution and High Flux Soft x-Ray Beamline at BESSY. *Rev. Sci. Instrum.* **2007**, *78* (12), 123102. https://doi.org/10.1063/1.2808334.

(15) Favaro, M.; Clark, P. C. J.; Sear, M. J.; Johansson, M.; Maehl, S.; van de Krol, R.; Starr, D. E. Spectroscopic Analysis with Tender X-Rays: SpAnTeX, a New AP-HAXPES End-Station at BESSY II. *Surf. Sci.* **2021**, *713*, 121903. https://doi.org/10.1016/j.susc.2021.121903.
